# CoREST complex inhibition alters RNA splicing to promote neoantigen expression and enhance tumor immunity

**DOI:** 10.1172/jci.insight.190287

**Published:** 2025-12-09

**Authors:** Robert J. Fisher, Kihyun Park, Kwangwoon Lee, Katarina Pinjusic, Allison Vanasse, Christina S. Ennis, Parisa Farokh, Scott B. Ficaro, Jarrod A. Marto, Hanjie Jiang, Eunju Nam, Stephanie Stransky, Joseph Duke-Cohan, Melis A. Akinci, Anupa Geethadevi, Eric Raabe, Ana Fiszbein, Shadmehr Demehri, Simone Sidoli, Chad W. Hicks, Derin B. Keskin, Catherine J. Wu, Philip A. Cole, Rhoda M. Alani

**Affiliations:** 1 Department of Dermatology, Boston University Chobanian and Avedisian School of Medicine, Boston, Massachusetts, USA.; 2 Division of Genetics, Departments of Medicine and Biological Chemistry and Molecular Pharmacology, Harvard Medical School and Brigham and Women’s Hospital, Boston, Massachusetts, USA.; 3 Department of Medical Oncology, Dana-Farber Cancer Institute, Harvard Medical School, Boston, Massachusetts, USA.; 4 Cancer Center, Boston University Chobanian and Avedisian School of Medicine, Boston, Massachusetts, USA.; 5 Center for Cancer Immunology, Krantz Family Center for Cancer Research, and; 6 Cutaneous Biology Research Center, Department of Dermatology, Massachusetts General Hospital and Harvard Medical School, Boston, Massachusetts, USA.; 7 Department of Cancer Biology, Center for Emergent Drug Targets, and Blais Proteomics Center, Dana-Farber Cancer Institute, Department of Pathology, Brigham and Women’s Hospital and Harvard Medical School, Boston, Massachusetts, USA.; 8 Department of Biochemistry, Albert Einstein College of Medicine, The Bronx, New York, USA.; 9 Division of Pediatric Oncology, Johns Hopkins University School of Medicine and Johns Hopkins Hospital, Bloomberg Children’s Center, Baltimore, Maryland, USA.; 10 Department of Biology, Boston University, Boston, Massachusetts, USA.; 11 Department of Pharmacology, Physiology & Biophysics, Boston University Chobanian and Avedisian School of Medicine, Boston, Massachusetts, USA.; 12 Broad Institute of MIT and Harvard, Cambridge, Massachusetts, USA.; 13 Translational Immunogenomics Laboratory, Dana-Farber Cancer Institute, Boston, Massachusetts, USA.; 14 Department of Computer Science, Metropolitan College, Boston University, Boston, Massachusetts, USA.; 15 Section for Bioinformatics, Department of Health Technology, Technical University of Denmark, Lyngby, Denmark.

**Keywords:** Dermatology, Oncology, Therapeutics, Cancer immunotherapy, Epigenetics, RNA processing

## Abstract

Epigenetic macromolecular enzyme complexes tightly regulate gene expression at the chromatin level and have recently been found to colocalize with RNA splicing machinery during active transcription; however, the precise functional consequences of these interactions are uncertain. Here, we identify unique interactions of the CoREST repressor complex (LSD1-HDAC1-CoREST) with components of the RNA splicing machinery and their functional consequences in tumorigenesis. Using mass spectrometry, in vivo binding assays, and cryo-EM, we find that CoREST complex–splicing factor interactions are direct and perturbed by the CoREST complex selective inhibitor, corin, leading to extensive changes in RNA splicing in melanoma and other malignancies. Moreover, these corin-induced splicing changes are shown to promote global effects on oncogenic and survival-associated splice variants, leading to a tumor-suppressive phenotype. Using machine learning models, MHC IP-MS, and ELISpot assays, we identify thousands of neopeptides derived from unannotated splice sites that generate corin-induced splice-neoantigens that are demonstrated to be immunogenic in vitro. Corin is further shown to reactivate the response to immune checkpoint blockade, effectively sensitizing tumors to anti–PD-1 immunotherapy. These data position CoREST complex inhibition as a unique therapeutic opportunity that perturbs oncogenic splicing programs while also creating tumor-associated neoantigens that enhance the immunogenicity of current therapeutics.

## Abstract 2

We identify novel regulatory functions of the CoREST complex in pre-mRNA splicing; moreover, CoREST inhibition induces immunoreactive splice-neoantigens that promote tumor-associated immunogenicity.

## Graphical abstract

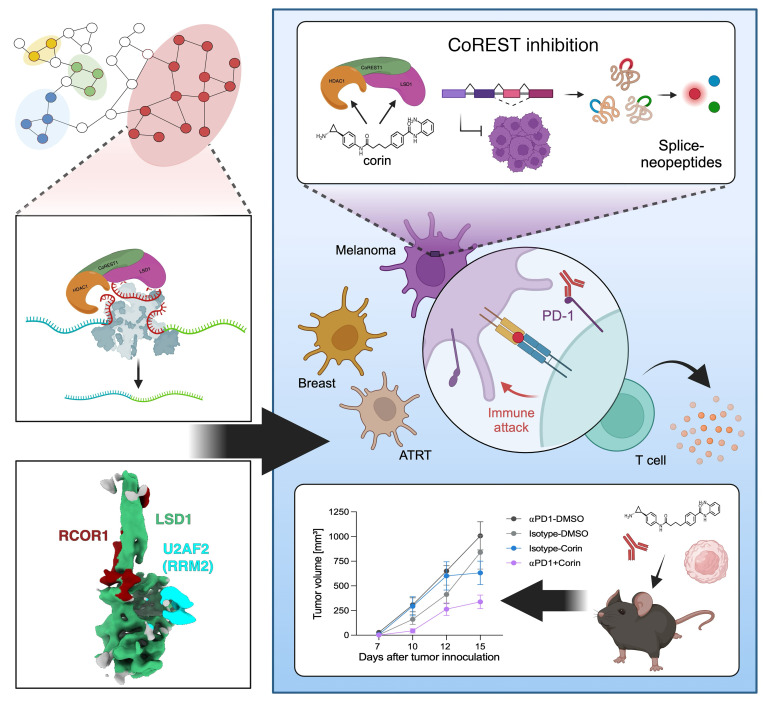

## Introduction

Pre-mRNA splicing is essential for the expression of > 95% of human genes that encode a diverse array of highly lineage and context-dependent protein variants (1, 2). Splicing changes are commonly seen in cancer, where tumors have been noted to have up to 30% more alternative splicing events (ASEs) than corresponding normal tissues, suggesting particular growth advantages of such changes (3, 4). The prevalence and effect of tumor-associated splicing changes have been recognized as a critical hallmark of cancer (5–7), and splicing mechanisms have been implicated in resistance to cancer therapies (8, 9). Additionally, alternative RNA splicing increases proteomic diversity in tumors and induces expression of potential neoantigens for tumor-specific detection. These splice-neopeptides have been leveraged as immunotherapy targets (3, 10–12) and shown to elicit antigen-specific T cell activity to trigger an antitumor response (13, 14); therefore, chemical induction of RNA splice modifications (15, 16) and therapies targeting pre-mRNA splicing in cancer are of great interest (11, 15, 17).

Epigenetic macromolecular enzyme complexes tightly control gene expression at the chromatin level and have been shown to regulate transcript diversity through direct regulation of RNA splicing (18–25). RNA splicing occurs largely cotranscriptionally, and alternative splice site choice is influenced by RNA polymerase II (Pol II) elongation rate, chromatin remodelers, and histone deacetylases (2, 22, 26); however, the precise role of epigenetic complexes in RNA splicing is uncertain. During active transcription, histone modifying enzymes, nucleosome remodelers, general transcription factors, and the splicing machinery all localize within the same chromatin environment allowing for cotranscriptional crosstalk (20). Epigenetic proteins have been found to modulate splicing by altering splicing factor gene expression (27, 28) and function (29), interacting with spliceosomal and ribonucleoprotein complexes (30), regulating the acetylation states of splicing-associated histone marks and splicing factors (30), and altering novel splice junctions with transposable elements (31).

The CoREST epigenetic repressor complex has core subunits HDAC1 (or close paralog HDAC2), LSD1 demethylase, and the scaffold protein CoREST and was originally identified as a corepressor complex for the transcription factor REST (32). CoREST functions as a gene silencing complex through its histone deacetylase roles and demethylation of H3K4me (33, 34) and has been demonstrated to deacetylate the C-terminal domain (CTD) of the catalytic subunit of RNA Pol II as part of its transcriptional repressing effects (35). We have previously described the dual LSD1/HDAC1 CoREST-selective inhibitor, corin (36), and demonstrated its antineoplastic activity in melanoma (36, 37), diffuse glioma (38), malignant peripheral nerve sheath tumor (39), and colon cancer (40). Here, we explore the role of the CoREST complex in RNA splicing regulation in melanoma. We uncover interactions between the CoREST complex and splicing factors, characterize these interactions using cryogenic electron-microscopy (cryo-EM) and define a noncanonical role for CoREST in RNA splicing regulation. We find that corin widely disrupts the CoREST complex–mediated splicing program in melanoma leading to induction of immunogenic neoantigen expression in cell lines. We further demonstrate that CoREST complex inhibition significantly reactivates the response to checkpoint blockade immunotherapy in immune cold tumors. We therefore suggest that CoREST complex inhibition represents a unique therapeutic opportunity in cancer.

## Results

### The CoREST complex interacts with RNA splicing factors.

**Figure 1:**
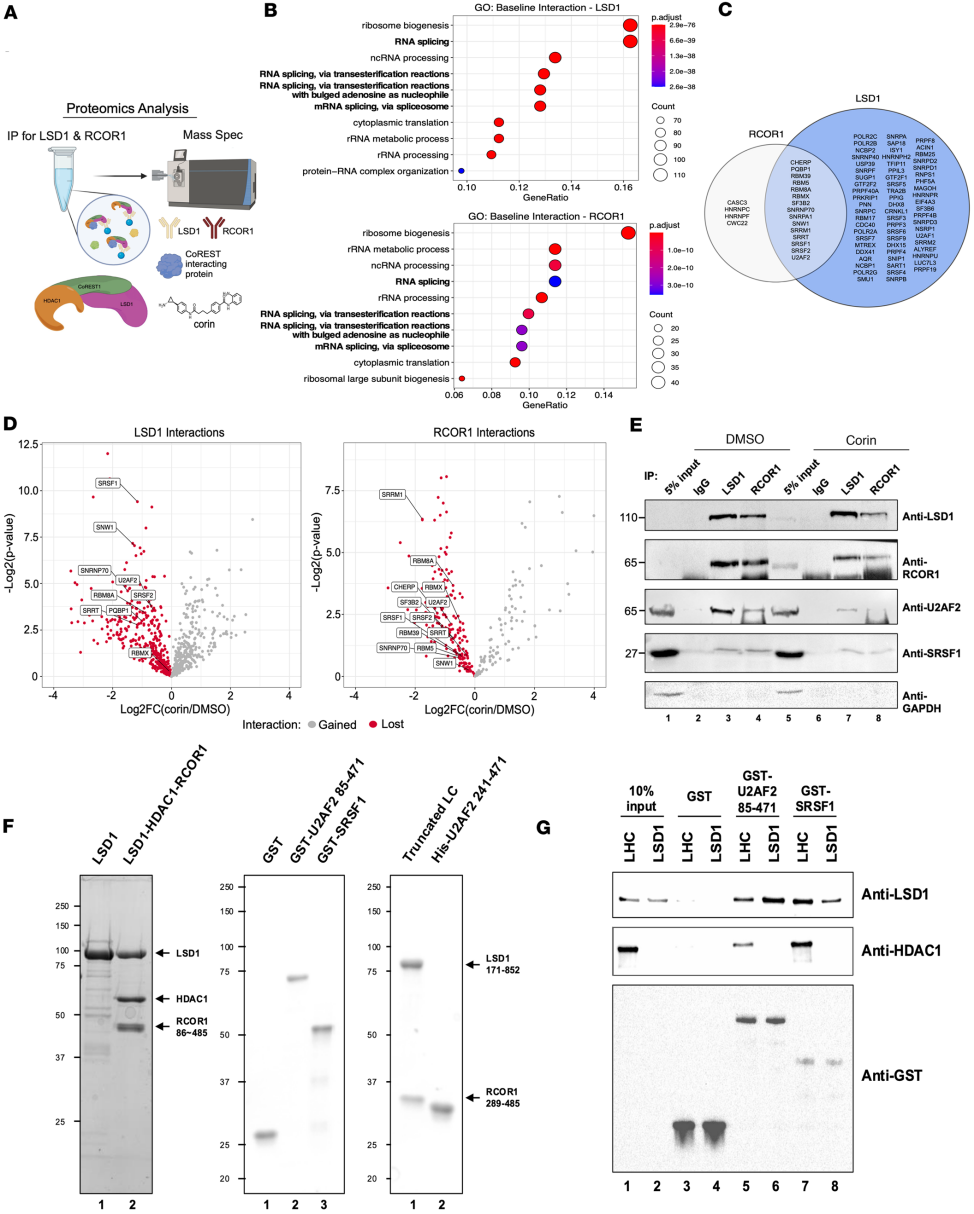
The CoREST complex interacts with splicing factors. (**A**) Overview of immunoprecipitation–mass spectrometry experiment completed in duplicate. (**B**) Gene ontology analysis for proteins found to have significant baseline interactions (Log_2_FC > 1, *P* < 0.05) with LSD1 (top) and RCOR1 (bottom). Enrichment analysis was performed using the hypergeometric test with multiple test correction by the Benjamini-Hochberg method. (**C**) Venn diagram of RNA splicing proteins found to significantly interact with LSD1 and RCOR1. (**D**) Volcano plots of LSD1 (left) and RCOR1 (right) baseline interactions lost with corin treatment (red). Labeled points are proteins from the overlap in **C**. Statistical analysis was performed using the heteroscedastic *t* test. (**E**) IP-WB analysis of CoREST complex–U2AF2 and CoREST complex–SRSF1 interactions with DMSO or corin treatment (24 hours, 2.5 μM). (**F**) SDS-PAGE and Coomassie Blue staining of purified proteins. (**G**) GST pull-down assay using purified GST-tagged U2AF2 (amino acids 85–471) or SRSF1, and purified CoREST complex (LHC) or LSD1 protein.

The CoREST complex influences gene expression and is recruited to target genes through interactions with lineage-specific transcription factors, core transcription complex components, and chromatin-associated proteins (33, 41) and has been shown by us and others to promote tumor growth in melanoma and other cancers (36–40). In order to further define the mechanism of CoREST complex effects in melanoma, we evaluated its protein interactions in the setting of the CoREST inhibitor, corin (2.5 μM, 24 hours), versus DMSO control in 2 melanoma cell lines (1205Lu and 451Lu) (Figure 1, A–D, and Supplemental Tables 1 and 2; supplemental material available online with this article; https://doi.org/10.1172/jci.insight.190287DS1). The LSD1/RCOR1 endogenous protein interactome was evaluated by liquid chromatography with tandem mass spectrometry (LC-MS/MS). LSD1 and RCOR1 were found to interact with all known CoREST complex subunits as well as the chromatin structural organizer, CTCF (24), and several members of the SWI/SNF (BAF) complex, as has been previously described (42). Interestingly, the CoREST complex–BAF interactions were disrupted by corin in both cell lines (Supplemental Table 2). Pathway analysis of proteins that showed elevated binding to LSD1 and RCOR1 over background across both cell lines identified significant enrichment for RNA splicing-related pathways, suggesting CoREST complex–splicing factor interactions in melanoma that are disrupted by corin (Figure 1B).

To identify high-confidence CoREST complex–interacting splicing factors (SFs), we overlapped SFs found in both LSD1 and RCOR1 pulldowns (Figure 1C) and then stratified the data by proteins that gain or lose interaction with the CoREST complex upon corin treatment (Figure 1D and Supplemental Table 2). We identified 15 SFs that interacted with the CoREST complex (Figure 1C) and found that corin disrupts 14 of the 15 CoREST complex–SF interactions (Figure 1D). To validate these findings, we selected 2 candidate SFs, U2 small nuclear RNA auxiliary factor 2 (U2AF2) and Serine/Arginine Splicing Factor 1 (SRSF1) based on the list of 15 CoREST complex–interacting SFs cross-referenced with an HDAC1 IP-MS dataset and their known functions in cancer (43, 44), and we performed IP-WB in an additional melanoma cell line (SKMEL5) (Figure 1E). The CoREST complex was found to interact with both U2AF2 and SRSF1 by IP-WB, and corin treatment greatly reduced CoREST-U2AF2 interactions (Figure 1E). To determine whether the interaction between CoREST and U2AF2 is direct, we performed a GST pull-down assay using purified proteins (Figure 1, F and G). Full-length LSD1 protein was purified from a bacterial expression system, whereas the recombinant CoREST complex (LHC), which included LSD1, HDAC1, and RCOR1 (amino acids 86-485) proteins, was obtained from overexpression in human 293F cells, and GST-tagged U2AF2 (amino acids 83-482) and SRSF1 proteins were generated from bacterial expression (Figure 1F). GST pull-down assays show that both LHC and LSD1 bind to U2AF2 and SRSF1, revealing direct interactions of LHC with U2AF2 and SRSF1, with LSD1 playing a key role in this interaction (Figure 1G).

### Cryo-EM structure of the CoREST complex bound to U2AF2.

In order to clarify the nature of CoREST complex interactions with the splicing machinery, we sought to develop structural details relevant to these complexes using cryo-EM analysis. Our studies were focused on the CoREST complex–U2AF2 interaction, as U2AF2 displayed tighter binding with the CoREST complex in AlphaFold (45) predictions compared with SRSF1. We determined the cryo-EM structure of U2AF2 bound to LSD1 + RCOR1 (Supplemental Figure 1A) at a global resolution of 5.14 Å (Supplemental Figure 1B).

**Figure 2:**
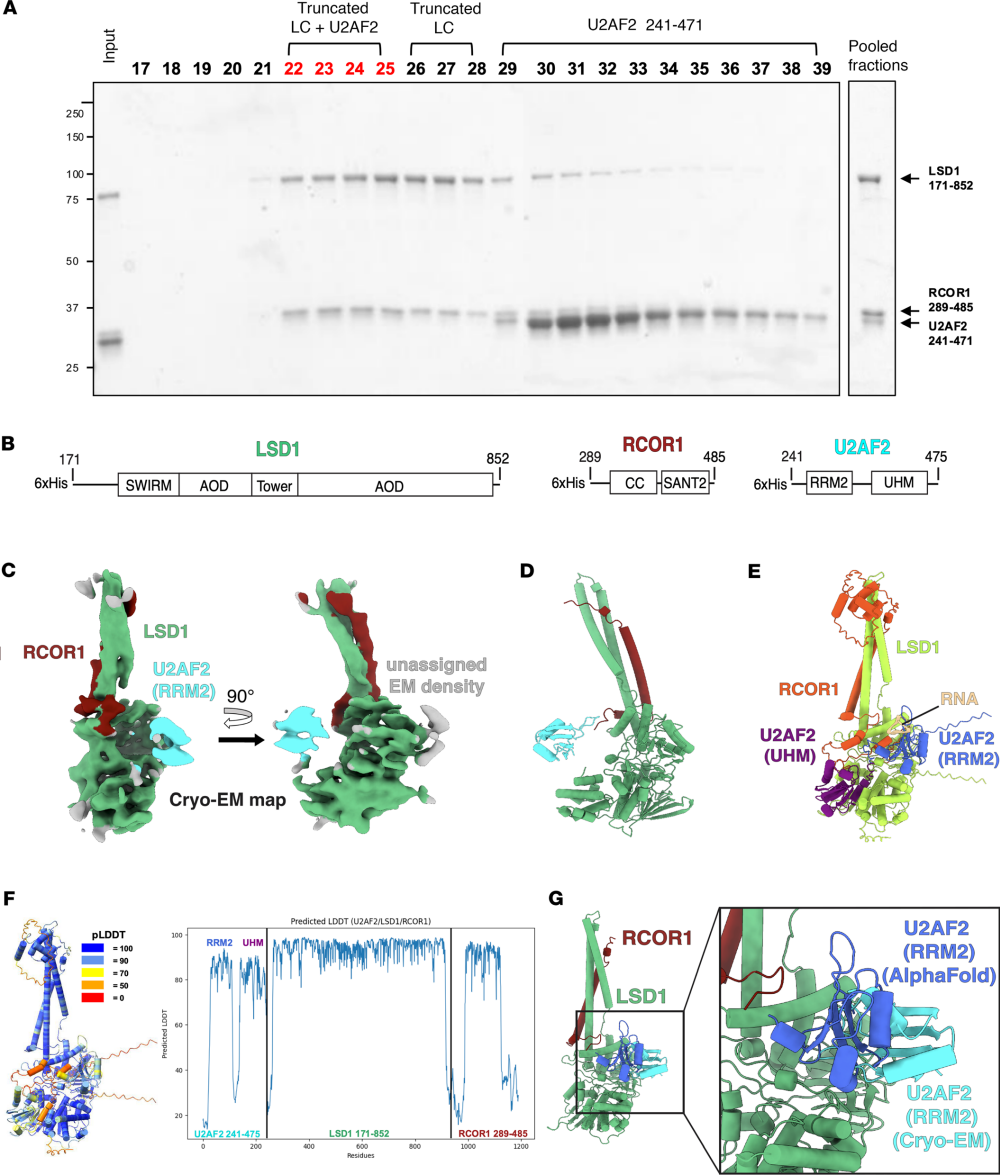
Cryo-EM structure and AlphaFold prediction of U2AF2 bound to LSD1 + RCOR1. (**A**) Size exclusion chromatography for cryo-EM sample preparation. The early fractions (fractions 22–25), containing LSD1, RCOR1, and U2AF2, were pooled, concentrated, and subsequently used for cryo-EM analysis. (**B**) Domain schematic of all protein components used for cryo-EM sample preparation. (**C**) Cryo-EM map of the RRM2 domain of U2AF2 bound to the LSD1 + RCOR1 complex. (**D**) Cryo-EM model in cartoon view representing the cryo-EM map shown in **C**. (**E**) AlphaFold model representing the prediction shown in **D**. (**F**) Predicted local distance difference test (pLDDT) plot of the AlphaFold multimer prediction incorporating LSD1, RCOR1, U2AF2, and RNA. (**G**) Superimposition of the RRM2 domain of U2AF2 from AlphaFold multimer over our cryo-EM structure of LSD1 + RCOR1 + U2AF2. Zoomed-in box shows the degree of similarity in position between the 2 RRM2 globular domain models.

To prepare cryo-EM samples, we purified the LSD1 (aa 171–852) and RCOR1 (aa 286–485) complex (truncated LC) and U2AF2 (aa 241–471) proteins separately (Figure 1F), mixed truncated LC with U2AF2 (aa 241–475) at a 1:5 ratio in the absence of crosslinker and performed size-exclusion purification to isolate the U2AF2-bound LSD1/RCOR1 complex (Figure 2A). The chromatogram showed a peak corresponding to the LSD1-RCOR1-U2AF2 complex, as well as 2 separate peaks for LSD1-RCOR1 and U2AF2 alone, confirming that the LSD1-RCOR1 complex binds U2AF2. Fractions containing the LSD1-RCOR1-U2AF2 complex were pooled (22–25) and samples were frozen onto cryo-EM grids. Of note, the U2AF2 (aa 241-475) construct used for the cryo-EM sample contains 2 structured globular domains, the RNA Recognition Motif 2 (RRM2) and U2AF2 Homology Motif (UHM) (Figure 2B).

We observed well-resolved EM density for the LSD1 + RCOR1 complex, low-resolution density for U2AF2 adjacent to the side of LSD1, and some unassigned very low–resolution density in contact with the opposite side of LSD1 (Figure 2C). The resolution of the EM density corresponding to LSD1 + RCOR1, at ~5.0–6.0 Å (Supplemental Figure 1C), was sufficient to unambiguously rigid fit the crystal structure of the LSD1 + RCOR1 complex (46) within the EM map (Supplemental Figure 1D). Therefore, our cryo-EM structure — the first cryo-EM structure, to our knowledge, of the LSD1 + RCOR1 complex — appears to adopt a similar conformation to the crystallized form of the LSD1 + RCOR1 complex (46, 47).

The resolution of the adjacent U2AF2 EM density, at 6.0–7.0 Å, was found to be lower than the LSD1 + RCOR1 complex (Supplemental Figure 1C). We therefore compared the relative size of the 2 U2AF2 globular domains within the EM map (Supplemental Figure 1, E and F) and determined that the adjacent U2AF2 density is likely to be the RRM2 domain of U2AF2, as opposed to the UHM domain. The resolution for the RRM2 EM density was not high enough to determine an accurate orientation of the model within the EM map, likely due to its extensive conformational heterogeneity; however, we were able to determine that the structured RRM2 domain does not directly contact LSD1 (Figure 2, C and D). The additional EM density contacting LSD1 opposite the RRM2 domain could not be assigned (Figure 2C), although it is possible that the unassigned density may represent the UHM domain of U2AF2.

To better understand the mechanism of RNA recognition by U2AF2 in the context of the LSD1 + RCOR1 + U2AF2 complex, we performed an AlphaFold3 (45) multimer calculation of LSD1 + RCOR1 + U2AF2 + RNA. As an input, we provided the same sequences as the constructs of the LSD1, RCOR1, and U2AF2 employed in the cryo-EM analysis plus added an additional 11 bp of RNA. The AlphaFold prediction revealed a structured LSD1 + RCOR1 at very high confidence and showed the RRM2 and UHM domains of U2AF2 bound to LSD1 at moderately high confidence (Figure 2, E and F). The predicted local distance difference test (pLDDT), representing local structural confidence calculated by AlphaFold, shows that the 2 globular domains of U2AF2 (RRM2 and UHM) have high confidence scores, while the flexible linker loop connecting them has low scores (Figure 2F) demonstrating AlphaFold prediction of U2AF2 remaining folded in globular domains and associated with LSD1 + RCOR1 as observed in the cryo-EM structure. Superimposing the AlphaFold structure of the RRM2 domain of U2AF2 over our cryo-EM structure shows the AlphaFold model of RRM2 in close proximity to the cryo-EM model of RRM2 (Figure 2G). The AlphaFold prediction also revealed RNA bound to the RRM2 domain of U2AF2 (Figure 2E), a binding mode that has been previously observed in crystal structures of RNA bound to U2AF2 (48, 49). While we did not include RNA in our cryo-EM sample, the position of the RRM2 domain of U2AF2 could also accommodate bound RNA. To characterize the RRM2-UHM domains of U2AF2 in the interaction with LSD1/RCOR1, microscale thermophoresis (MST) was performed. The His-tag U2AF2 fragments (aa 85–471 or 241–471) were labeled with a Cy5 dye at lysine residues (Fluor-U2AF2) using a reactive NHS-ester dye. The labeling was verified using a capillary scan on the MST instrument (Supplemental Figure 1, G and H). Subsequently, the labeled U2AF2 fragments were mixed with serially diluted truncated LSD1/RCOR1, and MST traces were measured to calculate the dissociation constant (*K*_D_) values (Supplemental Figure 1, I and J). We found that U2AF2 241–471, composed of RRM2 and UHM, exhibited a *K*_D_ value of 16 μM comparable with that of U2AF2 85–471 (*K*_D_ 14 μM), indicating that U2AF2 directly binds to truncated LSD1/RCOR1 with a moderate binding affinity, and that the RRM2 and UHM domains of U2AF2 are critical for this interaction. Additionally, a GST pull-down assay using the RRM2 fragment of U2AF2 confirmed that the RRM2-only fragment binds to truncated LSD1/RCOR1 (Supplemental Figure 1H), consistent with Cryo-EM and AlphaFold data, further supporting the role of the U2AF2 RRM2 domain in binding to CoREST.

### CoREST complex regulation of splicing factor gene expression.

**Figure 3:**
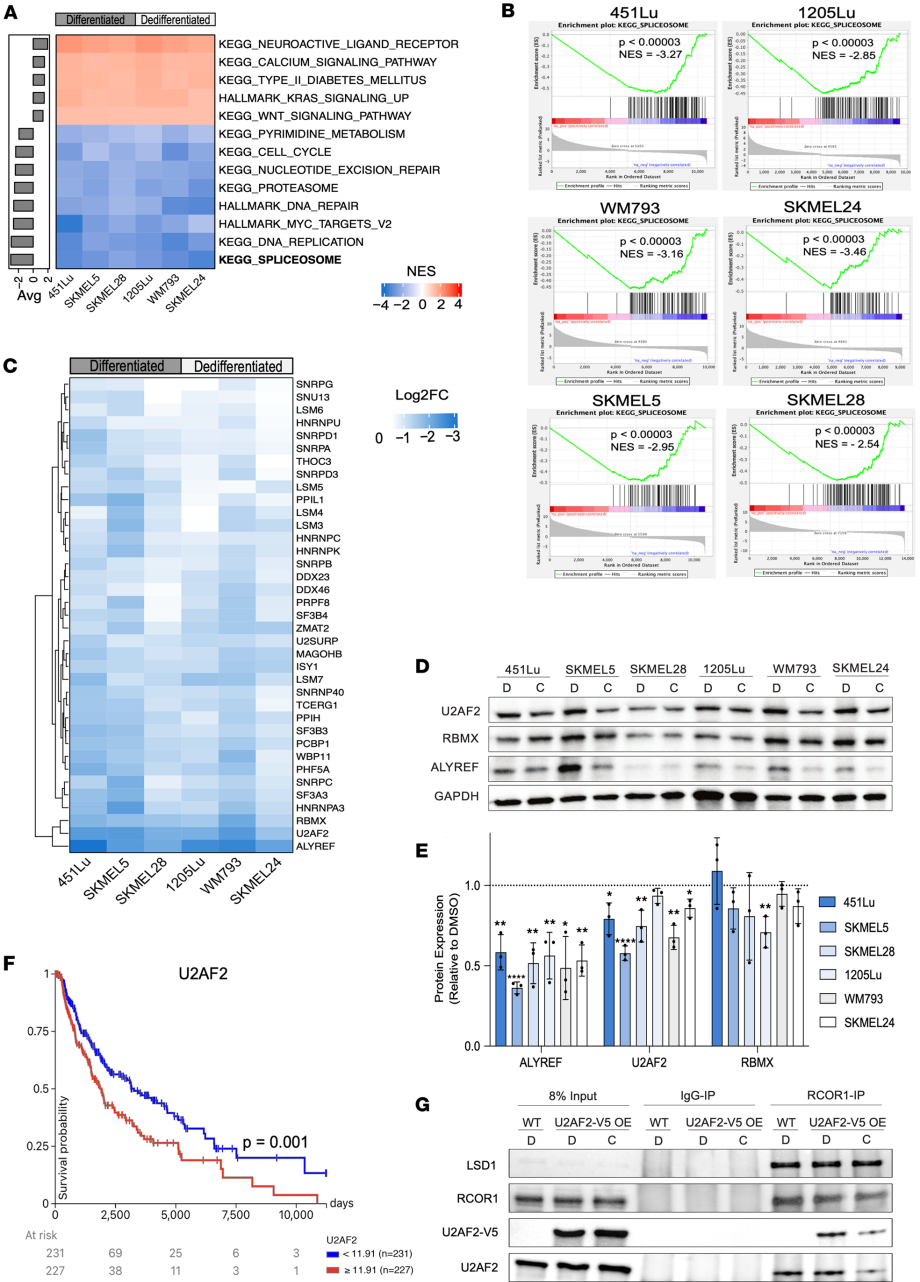
CoREST transcriptionally regulates splicing factor gene expression. (**A**) Heatmap of significant KEGG and Hallmark pathways (*P* < 0.05) across 6 melanoma cell lines treated with corin (24 hours, 2.5 μM) in duplicate. Pathways are ranked by average normalized enrichment score (NES), and cell lines are grouped based on phenotype. (**B**) Gene Set Enrichment Analysis plots for each cell line showing a significant negative enrichment for “KEGG Spliceosome.” (**C**–**E**) Heatmap of splicing factor genes significantly downregulated by corin treatment (*q* < 0.01, Log2FC < –0.5) across all 6 melanoma cell lines clustered using Euclidean distance (**C**). Representative Western blot of downregulated splicing factors across 6 melanoma cell lines treated with DMSO (**D**) or corin (**C**) and quantification of biological replicates (*n* = 3) (**E**). Data are shown as mean ± SD. (**F**) Kaplan-Meier plot of TCGA-SKCM patient survival based on median U2AF2 expression. Significance was determined using a log-rank test. (**G**) IP-WB analysis of CoREST-U2AF2 interactions following DMSO (**D**) and corin (**C**) treatment (24 hours, 2.5 μM) in V5-tagged U2AF2 overexpression SKMEL5 cells.

Given our findings of the CoREST complex binding to components of the RNA splicing machinery, which is diminished in the context of the CoREST inhibitor, corin, and the fact that expression of RNA binding proteins (RBPs) is widely dysregulated in solid tumors (50), we sought to determine whether the CoREST complex may directly affect the transcription of pre-mRNA splicing factors. We conducted RNA-Seq on 6 melanoma cell lines treated with corin (2.5 mM, 24 hours), 3 tumor cell lines designated as MITF^hi^/AXL^hi^ cell lines (differentiated phenotype) (51) and 3 tumor cell lines designated as MITF^lo^/AXL^hi^ (dedifferentiated phenotype) (51). We then performed gene set enrichment analysis (GSEA) to identify commonly enriched pathways following corin treatment across all cell lines regardless of phenotypic status. Remarkably, we identified the KEGG Spliceosome pathway as the most common downregulated pathway across all 6 cell lines (Figure 3, A and B), suggesting that CoREST inhibition downregulates splicing factor gene expression in melanoma regardless of molecular phenotype. Further analysis of the splicing factor genes affected by corin treatment revealed that, although many U2-related and early spliceosomal genes were downregulated, a broad range of spliceosome and trans-acting splicing regulators were also downregulated suggesting widespread impact on the splicing machinery (Figure 3C and Supplemental Figure 2A). Notably, 29% of genes in the KEGG Spliceosome gene set were found to be consistently downregulated by corin without correlation to melanoma phenotype, highlighting the broad effect of CoREST on splicing factor gene regulation. To validate these findings, Western blotting was performed on the 3 most downregulated genes, where U2AF2 and ALYREF were both noted to be significantly decreased in expression at the protein level following corin treatment (Figure 3, D and E, and Supplemental Figure 2B). This is particularly of interest since patients with melanoma (The Cancer Genome Atlas Skin Cutaneous Melanoma [TCGA SKCM]) with decreased U2AF2 expression have significantly prolonged survival (*P* = 0.001; Figure 3F).

To determine whether corin-associated decreases in splicing factor expression were due to direct transcriptional changes, we performed PRO-Seq on SKMEL5 cells treated with corin and used the 3’ nascent RNA reads to conduct differential gene expression analysis (Supplemental Figure 2C). We found the KEGG Spliceosome pathway to be significantly negatively enriched in the setting of corin treatment with over 50% of splicing factor genes downregulated in the RNA-Seq also downregulated in the PRO-Seq (Supplemental Figure 2D). This suggests that corin-associated splicing factor downregulation is due to direct effects on mRNA synthesis rather than transcript stability. Since CoREST is an epigenetic repressor complex and the majority of corin effects on tumor cells are associated with transcriptional upregulation (36–39), it is anticipated that these transcriptional repressive effects of corin on splicing factor genes are principally associated with indirect transcriptional changes. Since we were able to demonstrate decreased transcription of SF genes in the setting of corin in melanoma cells, we sought to determine whether the loss of CoREST complex–SF protein interactions in the setting of corin was due to decreased expression of splicing factor proteins or a disruption of CoREST complex–SF binding. Using V5-tagged U2AF2 overexpression in SKMEL5 melanoma cells, we found that binding of V5-U2AF2 to CoREST (RCOR1) was significantly inhibited in the setting of corin treatment without a change in V5-U2AF2 expression, supporting a specific inhibitory effect of corin on U2AF2 binding to CoREST independent of the transcriptional effects on U2AF2 (Figure 3G).

### Corin induces RNA splicing changes in melanoma.

**Figure 4:**
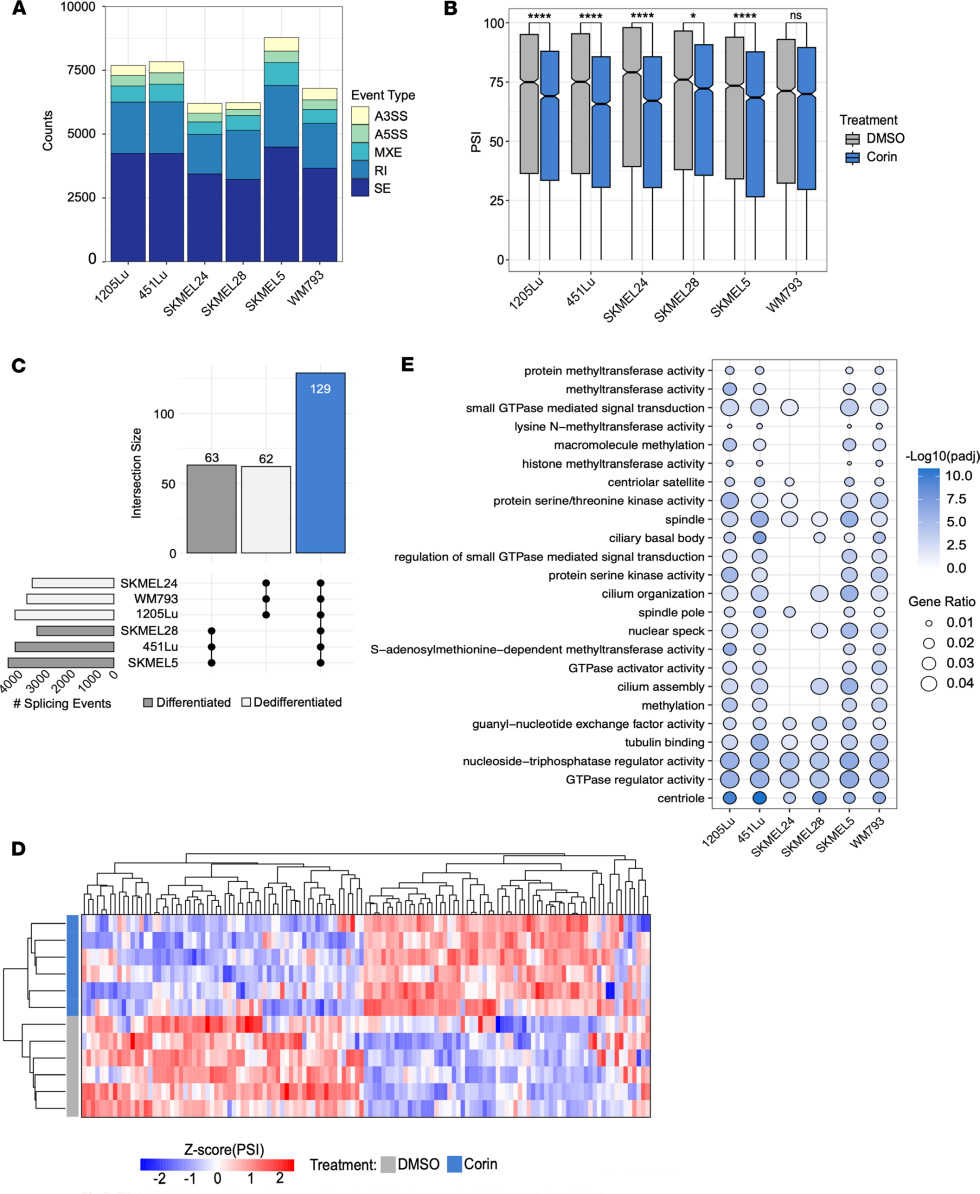
Corin induces RNA splicing changes in melanoma. (**A**) Summary of significant RNA splicing changes across 6 melanoma cell lines treated with corin (ΔPSI ≥ |0.1|, *q* < 0.05) in duplicate. (**B**) PSI levels for all significant SE events following DMSO and corin treatment. Statistical comparisons were performed using a 2-sample *t* test to assess differences in PSI value between treatment groups within each cell line. *P* values were adjusted for multiple comparisons using the Bonferroni correction (*P_adj_ < 0.05, ****P_adj_ < 0.0001). (**C**) UpSet plot of skipped exon (SE) events that are exclusive to the differentiated phenotype, dedifferentiated phenotype, or shared by all cell lines (blue). (**D**) Unsupervised hierarchical clustering heatmap based on Euclidian distance of shared skipped exon inclusion levels. Rows are melanoma cell lines clustered by treatment and columns are shared inclusion events. (**E**) Gene ontology dot plot of the top pathways affected by corin-induced differential exon inclusion across all cell lines (median *P*_adj_ < 0.01). Enrichment analysis was performed using the hypergeometric test with multiple test correction by the Benjamini-Hochberg method.

Given the observed CoREST complex–U2AF2 interactions and corin’s effect on splicing factor gene expression, we next sought to determine the functional effects of corin on RNA splicing across a panel of melanoma cell lines. Two computational tools were used to analyze our RNA-Seq data for changes in splicing events. rMATS (52) was applied to detect alterations in skipped exons (SE), 3′ splice sites (3′SS), 5′ splice sites (5′SS), and mutually exclusive exons (MXE), while AltAnalyze (53) was used to identify changes in retained introns (RI). Using these methods, we identified thousands of splicing events modified by corin treatment across the 6 melanoma cell lines tested (Figure 4A, Supplemental Figure 3, and Supplemental Table 3), with skipped exons being the most frequently affected. When we compared percent spliced in (PSI) values of SE events between DMSO and corin treatment, all cell lines except WM793 showed a significant decrease in PSI distribution in the setting of corin treatment, indicating increased exon skipping with CoREST complex inhibition (Figure 4B). Additionally, when we compared the exon architecture of skipped versus included exons, skipped exons were significantly shorter, flanked by long introns, and in lower GC content regions of the genome (Supplemental Figure 4, A and B). This suggests that the CoREST complex may be involved in promoting exon inclusion, preferentially at defined exon architecture during cotranscriptional RNA splicing.

Next, we compared the unique SE events stratified by MITF status to determine whether the CoREST complex may be responsible for driving a phenotypic splicing program (Figure 4C). We identified several phenotype-specific SE events, with 63 events associated with the differentiated (MITF^hi^) state and 62 associated with the dedifferentiated (MITF^lo^) state; however, the overlap was minimal relative to the total number of SE events across the cell lines. We did, however, find 129 SE events that were commonly induced by corin treatment across all melanoma cell lines, which may be more generalizable targets of CoREST complex–specific splicing (Figure 4D). Although, these common SE events comprise a small fraction of the total, gene ontology analysis of corin-induced differential exon inclusion conducted independently on each cell line demonstrated that many of the same biological processes were affected by corin treatment such as cell motility, cytoskeletal structure, and GTPase activity (Figure 4E).

Given the effect of CoREST complex inhibition on SF binding to the CoREST complex and SF transcription, we next sought to determine the effect of corin on intron-containing pre-mRNA splicing activity by comparing the PSI distribution of intron retention events identified by AltAnalyze and found no significant directional increase in retention. We further compared the pathways affected by intron retention to those affected by exon skipping and found that RNA splicing factors are most differentially affected by corin-induced intronic splicing (Supplemental Figure 4, C and D). In order to define the splicing factors that drive the CoREST complex’s effect on RNA splicing, we utilized RNA-SPRINT (54) to estimate individual RBP activity across all 6 cell lines under each treatment condition and correlated the activity scores to the expression level of the corresponding RBP using Spearman’s rank correlation (Supplemental Figure 5A). Four candidates (SF3B4, HNRNPC, HNRNPK, and U2AF2) had significant coefficients (ρ > 0.5) in which decreased expression was strongly correlated with decreased RBP activity (Supplemental Figure 5, B–D), suggesting that CoREST complex–induced splicing programs could be largely attributed to the functions of these particular splicing factors. Overall, these findings highlight the complexity of RNA splicing regulation, as we see the CoREST complex engaged in transcriptional, translational, and posttranslational modifications that control the splicing process as well as specific splicing of splicing-associated transcripts.

**Figure 5:**
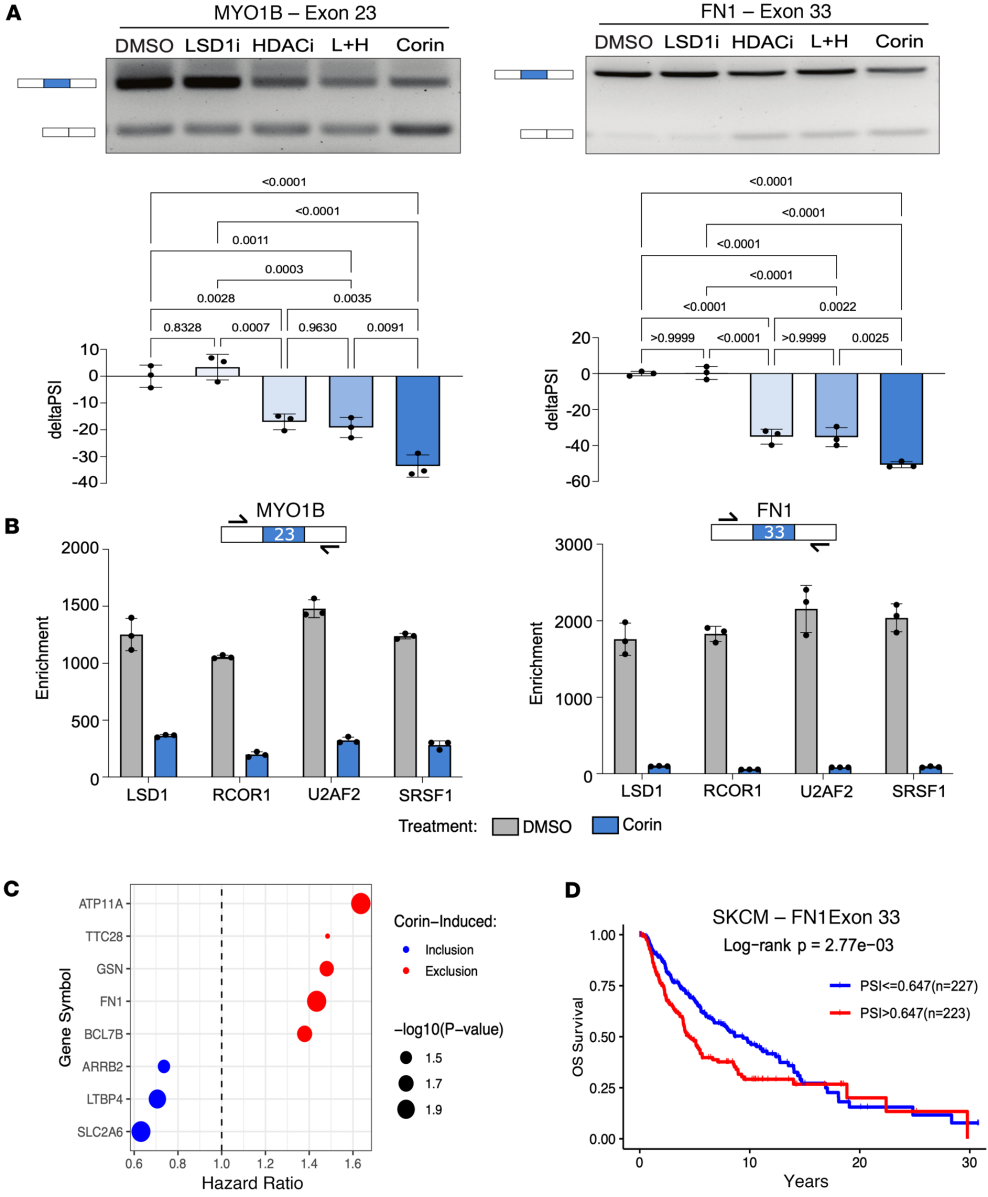
Corin induces splicing events associated with melanoma survival. (**A**) Representative RT-PCR gels and quantification comparing corin to single-agent inhibitors of HDAC and LSD1 in MYO1B and FN1 splicing. Statistical analysis of biological replicates (*n* = 3) was performed using a 1-way ANOVA with Tukey’s post hoc test for multiple comparisons. Data are shown as mean ± SD. (**B**) Representative RIP-qPCR biological replicate (*n* = 2) with 3 technical replicates of CoREST complex subunits and splicing factor occupancy at MYO1B and FN1 splice sites with corin treatment. Data are shown as mean ± SD. (**C**) Hazard ratios for corin-induced splicing events based on TCGA-SKCM survival data measured using the Cox proportional hazards regression. (**D**) Kaplan-Meier curve of FN1 exon 33 inclusion in TCGA-SKCM using the median PSI threshold. Significance was determined using a log-rank test.

Next, we validated 3 common SE events associated with cytoskeletal structure and cell motility functions with demonstrated oncogenic associations by RT-PCR (Figure 5A and Supplemental Figure 6, A–C). Myosin-1b (MYO1B) is a motor protein critical for actin filament organization and is crucial for neuronal development. *MYO1B* exon 23 exclusion has been associated with decreased cell migration and significant survival outcomes in glioblastoma mouse models (28). Fibronectin (*FN1*) exon 33 encodes extra domain A, which has been shown to increase tumor metastasis and is widely expressed in melanoma tissues compared with normal skin (55). Tight Junction Protein 1 (*TJP1* or *ZO-1*) exon 20 encodes the alpha domain whose skipped isoform is increased in breast, lung, and colon cancers (56) and has been shown to enhance actin stress fiber assembly, increase cell migration, and be induced during EMT by TGFB (56). In all instances, we found that corin significantly reversed tumor-associated splice isoforms (Figure 5A and Supplemental Figure 6, A–C); moreover, we found that corin significantly reversed CoREST complex–mediated splicing events to a greater extent than either MS-275 or GSK-LSD1 alone or the combination of inhibitors (Figure 5A and Supplemental Figure 6, A–C), suggesting targeted impacts of the dual-specificity CoREST complex inhibitor. Interestingly, we did not see any significant effect of U2AF2 on either LSD1 demethylase or LHC deacetylase activities in purified enzyme assays (Supplemental Figure 7, A–C), indicating a unidirectional effect of CoREST activity on the splicing machinery.

Based on our finding that CoREST complex–U2AF2 interactions are disrupted in the setting of corin, we hypothesized that these differential exon inclusion events may be directly influenced by CoREST-SF recruitment to RNA. Using RIP-qPCR with primers flanking *FN1* exon 33 and *MYO1B* exon 23, we found that CoREST complex subunits (LSD1 and RCOR1) and SFs (U2AF2 and SRSF1) not only bind RNA at these splice sites but also significantly lose occupancy with corin treatment (Figure 5B), suggesting that CoREST complex–SF recruitment to alternative splice sites can directly impact exon inclusion levels.

Given the effect of corin on tumor-associated splicing events, we analyzed the 129 corin-induced common SE events in the 6 melanoma cell lines for survival-association in TCGA-SKCM (human skin cutaneous melanoma) using OncoSplicing and a cox regression model (57). Eight of the common splicing events were found to be directionally beneficial for overall survival across patients with TCGA SKCM (Figure 5C) including *FN1* exon 33, where exon exclusion was found to be associated with significantly improved survival outcomes (Figure 5D).

As mRNA synthesis rates have been shown to have a significant effect on splicing, we were curious to see if CoREST could regulate RNA splicing by influencing the rate of active transcription. A previous study found that the CoREST complex can directly bind to RNA Pol II after the preinitiation complex is formed to deacetylate the CTD and effectively pause the polymerase (35). We hypothesized that this noncanonical role of the CoREST complex could alter RNA synthesis kinetics and, thus, alter exon inclusion levels. To test this, we analyzed our PRO-Seq data for changes in Pol II coverage at alternatively included exon splice sites in the setting of corin. Although we found that inhibiting the CoREST complex led to significant promoter-proximal pause release genome-wide (Supplemental Figure 8, A and B, and Supplemental Table 4), we did not note a significant difference in Pol II coverage between included or excluded exons (Supplemental Figure 8C), suggesting that these alternative splicing changes occur independent of a corin-induced kinetic effect on Pol II pause release.

### Corin induces RNA splicing changes across cancers.

**Figure 6:**
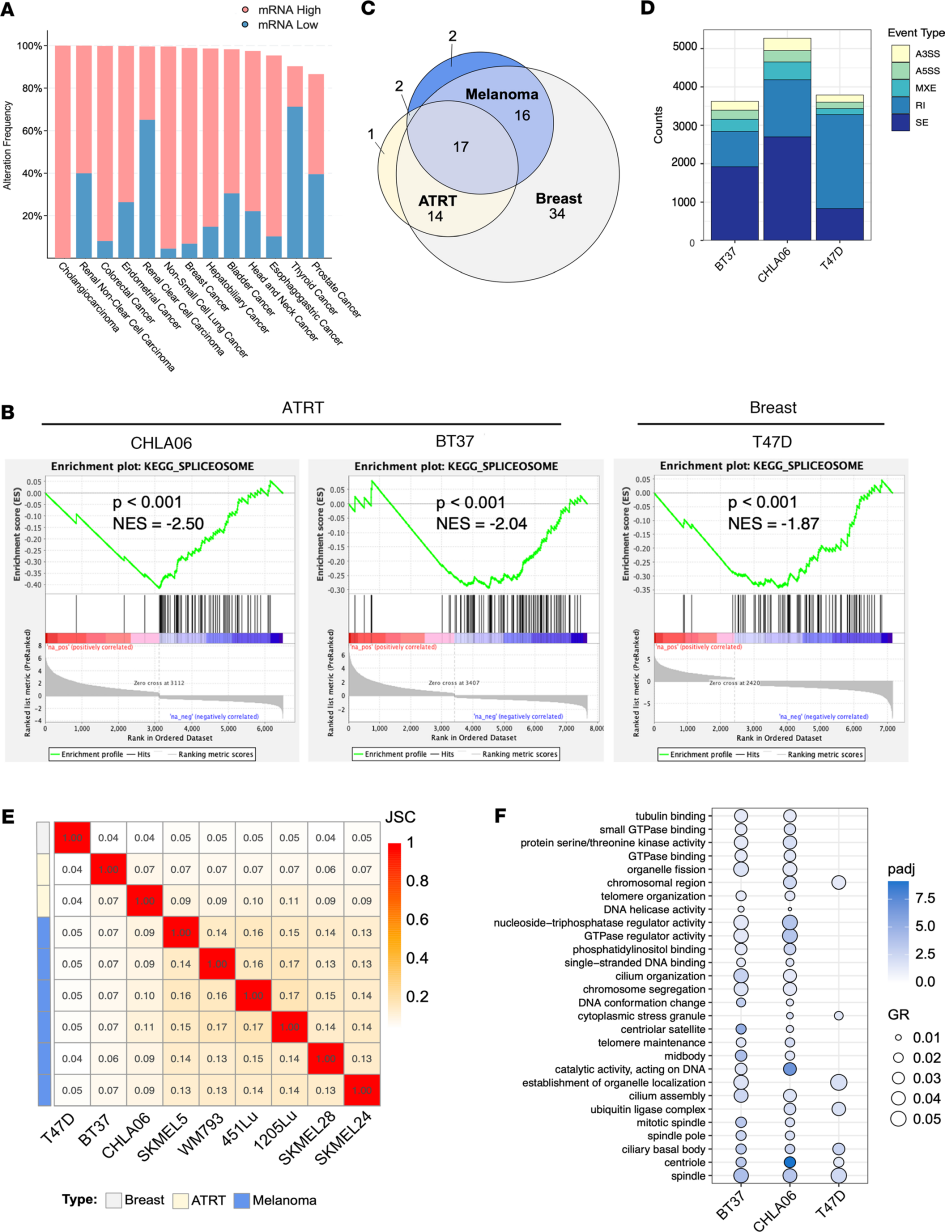
Corin affects RNA splicing across cancers. (**A**) Histogram depicting the frequency of altered mRNA expression alteration in CoREST complex–regulated RNA splicing factors between cancer and matched normal tissue from cBioPortal stratified by cancer type. Pink bars indicate the frequency of overexpression events, and blue bars indicate the frequency of downregulation events. (**B**) Gene Set Enrichment Analysis plots for “KEGG Spliceosome” in ATRT and breast cancer cell lines treated with corin. (**C**) Venn diagram of splicing factors significantly downregulated by corin in melanoma, ATRT, and breast cancer. (**D**) Summary of corin-induced RNA splicing changes in ATRT and breast cancer cells. (**E**) Jaccard similarity index comparing corin-induced splicing events across all cell lines and cancer types. (**F**) Gene ontology analysis of common significant pathways affected by corin-induced SE splicing events in ATRT and breast cancer. Enrichment analysis was performed using the hypergeometric test with multiple test correction by the Benjamini-Hochberg method.

Given the broad effect of corin on the splicing machinery in melanoma, we were interested in determining whether corin could modulate splicing across other cancers. Comparison of the expression levels of splicing factors transcriptionally downregulated by corin treatment between normal and matched tumor samples in cBioPortal revealed that many of these factors are significantly overexpressed in a diverse array of tumors (Figure 6A and Supplemental Figure 9), suggesting that this may be a driver event in cancers that could be targeted by corin. We next sought to determine whether corin-induced splicing changes could be seen in other cancers using a bioinformatic approach. Data obtained from 2 independent external RNA-Seq datasets using cancer cells treated with corin, an ER^+^ breast cancer dataset (42) and an Atypical Teratoid Rhabdoid Tumor (ATRT) dataset (58), were mined for gene expression changes and differential splicing analysis was performed using the pipeline previously described. Remarkably, we found that corin significantly affected splicing factor gene expression within these tumor cells to a similar extent to that seen in melanoma (*P* < 0.001; Figure 6, B and C, and Supplemental Table 5) and promoted thousands of RNA splicing differences across both tumor types (Figure 6D, Supplemental Figure 10A, and Supplemental Table 6). Moreover, the pathways affected by corin-induced splicing included many of the same cell motility and GTPase pathways seen in melanoma (Figure 6, E and F), suggesting not only that corin can induce splicing changes across cancer types but also that the CoREST complex may be involved in regulating splicing of specific biological processes in cancer. Notably, the differences in splicing events represented in Figure 6E are likely driven by intrinsic differences in epigenetic and transcriptional profiles rather than cell line–dependent CoREST-regulated splicing mechanisms. Concordance analysis of SE events shared between melanoma cell lines reveals strong concordance (>90%) as measured by PSI directionality (Supplemental Figure 10, A and B). Differential alternative promoter (AP) events, which most closely reflect distinct transcriptional programs, display even higher concordance (>95%) and cluster by MITF phenotypic status (Supplemental Figure 10C). Despite few phenotype-specific AP events (Supplemental Figure 10D) and diverse cell line–specific pathways (Supplemental Figure 10E), this concordance suggests CoREST-regulated splicing mechanisms are conserved but depend on intrinsic epigenetic landscapes.

### Corin-induced splice-neoantigens are presented on human MHC and are immunogenic.

**Figure 7:**
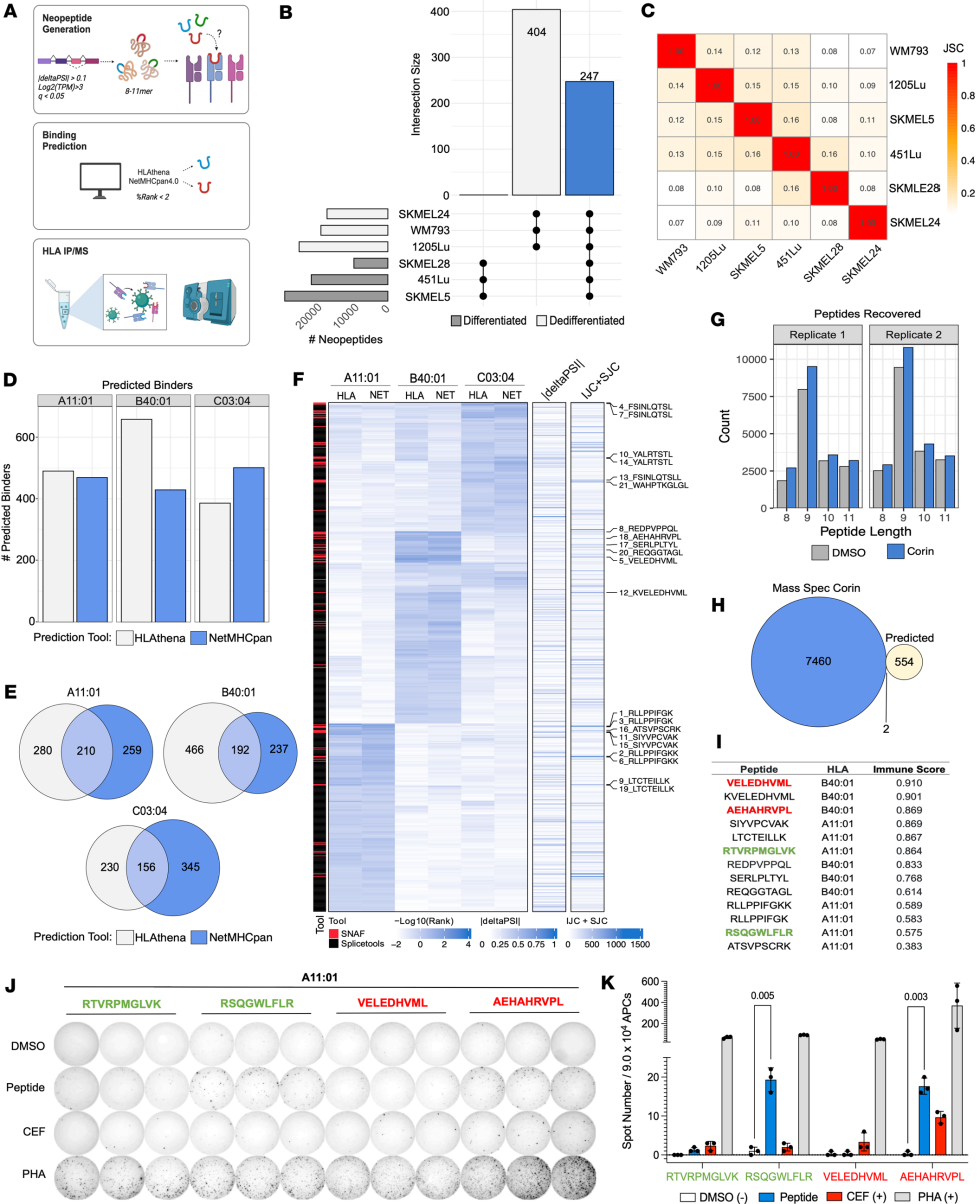
Corin-induced splicing produces neoantigens that bind human MHC and are immunogenic. (**A**) Overview of neopeptide discovery and MHC binding predictions (**B**) UpSet plot of neopeptides (8–11 mers) produced with corin treatment of melanoma cells that are exclusive to the differentiated phenotype, dedifferentiated phenotype, or shared by all cell lines (blue). (**C**) Jaccard similarity index comparing corin-induced neopeptide production across all melanoma cell lines. (**D**) Number of corin-induced neopeptides predicted to bind to SKMEL5 HLAs based on 2 prediction tools: HLAthena and NetMHCPan4.1 (%Rank < 2). (**E**) Overlap of SKMEL5 corin-induced neopeptide HLA binders for each allele predicted by both tools. (**F**) Heatmap showing binding scores, PSI values, and junction counts of predicted SKMEL5 corin-induced neopeptides identified by SNAF and Splicetools. The top 15 unique candidates are labeled. (**G**) Histogram plots of peptides recovered from MHC-IP/MS in SKMEL5 DMSO and corin-treated (72 hours, 1 μM) samples for each replicate (*n* = 2). (**H**) Identification of SKMEL5 corin-induced neopeptides recovered by MHC IP-MS. Corin-exclusive peptides are those identified from the IP-MS that appear in at least 1 corin replicate but neither DMSO replicate. Predicted peptides are those identified by binding scores in **F**. (**I**) Immunogenicity score predictions for neopeptide candidates. Green peptides are those identified from IP-MS and red peptides are additional candidates selected for immunogenicity validation assays based on immunogenic prediction and scores from **F**. (**J**) Ex vivo IFN-γ ELISpot assay for each candidate neopeptide tested with CEF and PHA positive controls. HLA-matched PBMCs were prestimulated with synthesized peptides (10 μg/mL) for 14 days in IL-2/IL-7 media. APCs were isolated from CD4 and CD8 depleted PBMCs, loaded with peptides (10 μg/mL), and seeded at a ratio of 3:1 with the prestimulated T cells in a 96 well ELISpot plate and analyzed for IFN-γ + T cells. (**K**) Quantification of ex vivo IFN-γ ELISpot assay illustrated in **J**. Statistical analysis was performed using multiple 2-tailed unpaired *t* tests. Data are shown as mean ± SD.

The splicing events discussed thus far have arisen from known annotations; however, we found that many events induced by corin were derived from unannotated splice sites and hypothesized that these unannotated events would have the potential to produce neopeptide products that may elicit an immune response in vivo (15, 16). To identify neopeptide candidates, we utilized a combination of predictive computational methods and HLA IP-MS validation (Figure 7A). SpliceTools (59) and SNAF (54) were used to identify 8–11 mer neopeptides produced from significant splicing events (*q* < 0.05, |ΔPSI| 0.1, log_2_TPM > 3). Remarkably, we identified thousands of neopeptide sequences induced by corin treatment across all melanoma cell lines and found them to be induced in a cell-line dependent manner (Figure 7, B and C) akin to the cell-line specific splicing patterns identified previously (Figure 4). In order to assess HLA binding of predicted neopeptides, we utilized 2 machine learning models trained on patient MS data: NetMHCpan4.1 (60) and HLAthena (61). We found hundreds of neopeptides predicted to bind (%Rank < 2) SKMEL5 cell HLAs by both tools (Figure 7D) and overlapped the neopeptides identified by each tool for each allele to identify the best candidate peptides (Figure 7E). Corin-associated neopeptides were then ranked based on a scoring system taking HLA binding rank, junction count, and ΔPSI value into consideration (Figure 7F and Supplemental Table 7). HLA-IP MS was used to investigate the 556 predicted neopeptide targets. Remarkably, we recovered over 10,000 8–11 mer peptides in each replicate of mass spec and found 7,462 corin-specific peptide products (Figure 7G and Supplemental Table 8). When we overlapped those products with the predicted 556 candidate neopeptides, we found 2 neopeptides that were produced from corin-specific splicing (Figure 7H). To assess if any neopeptides were missed, we also overlapped the 7,462 corin-specific peptides with the human proteome and found the 2 neopeptides already predicted. Lastly, we used the deep learning model, DeepImmuno (62), to predict the immunogenicity score of each peptide to its respective strongest binding HLA allele. Both peptides validated by mass spec were predicted to be immunogenic (immunogenicity score > 0.5) and were further tested in immunogenicity assays. Additionally, we ranked the top predicted peptides based on immunogenicity score and tested the top 2 unique peptides in our immunogenicity assay (Figure 7I). Since HLA-C alleles do not have adequate training data for accurate DeepImmuno immunogenicity predictions, we focused on HLA-A and HLA-B binding peptides.

To test the immunogenic potential of the 4 candidate neoantigens, we loaded high purity synthetic peptides on antigen presenting cells (APCs) derived from HLA-matched PBMCs (A11:01, B40:01, C03:04) and assessed prestimulated CD8^+^ T cell activation in an IFN-γ ELISpot assay (Figure 7, J and K, and Supplemental Figure 11, A and B). Two of the 4 neopeptides elicited strong T cell activation when loaded on A11:01 APCs compared with a DMSO negative control and CEF pool positive control confirming that corin treatment can induce splice-neoantigens in human melanoma cells that are immunogenic.

### Corin treatment sensitizes immune cold tumors to immunotherapy.

**Figure 8:**
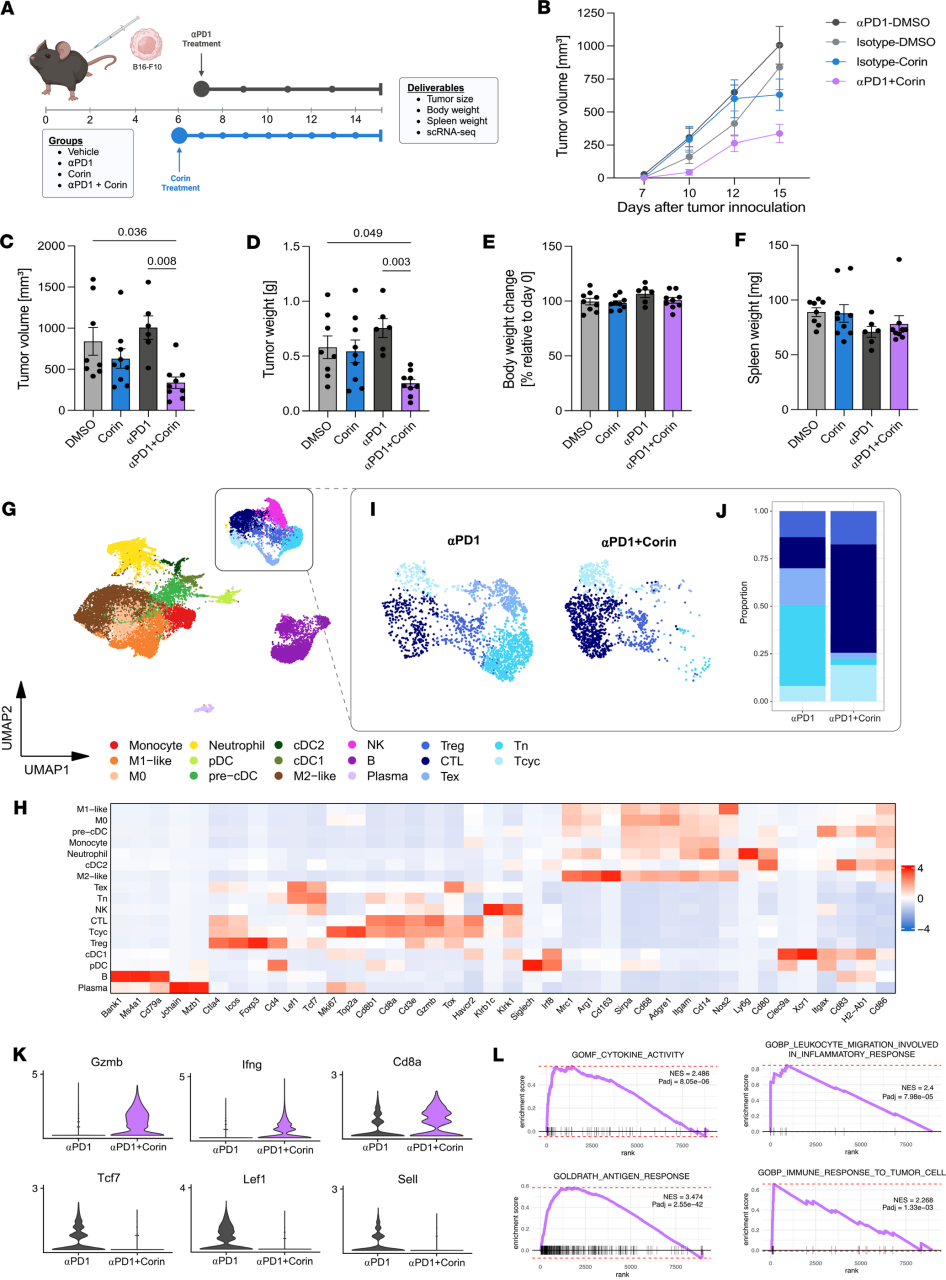
Corin sensitizes immune cold tumors to immunotherapy and promotes expansion of tumor infiltrating cytotoxic T cells. (**A**) Schematic for corin + immunotherapy combination treatment in a melanoma xenograft mouse model. Six- to 10-week-old female C57BL/6 mice were inoculated with 2.5 × 10^5^ B16-F10 cells. Mice were treated with 200 μg/mouse of corin or 200 μL vehicle control (5% DMSO/PBS) by daily i.p. injection starting from day 6 after tumor initiation. For anti–PD-1 treatment, mice were treated with 150 μg/mice anti–PD-1 or isotype control antibody 3 times/week starting from day 7 after tumor grafting. Ten mice were included in each treatment group. Tumors were measured 3 times/week and tumor volume, tumor weight, body weight change, spleen weight were measured. (**B** and **C**) Line plot and quantification of tumor volumes from day 7 to day 15 comparing DMSO, αPD-1, corin, and αPD-1 + corin treatment. (**D**) Histogram of tumor volumes depicted in **B**. (**E**) Histogram of body weight change relative to day 0 in animals treated with DMSO, αPD-1, corin, and αPD-1 + corin. (**F**) Histogram of spleen weights in animals treated with DMSO, αPD-1, corin, and αPD-1 + corin. Statistical analyses for **C**–**F** were performed using an ordinary 1-way ANOVA with Holm-Šídák’s correction for multiple comparisons. Data are shown as mean ± SD. (**G**) scRNA-Seq UMAP of the immune population (CD45^+^) isolated from B16-F10 melanomas. (**H**) Heatmap of the marker genes used to define immune subpopulations in **G**. (**I**) Subset UMAP of the T cell compartment comparing αPD-1 treatment to the combination of αPD-1 + corin. (**J**) Stacked bar plot of the T cell compartments in **I**. (**K**) Violin plots of significant DEGs (Log_2_FC > |1|, *P*_adj_ < 0.05) in T cell populations isolated from αPD-1 versus αPD-1 + corin-treated B16-F10 melanomas. (**L**) GSEA plots for T cell populations isolated from αPD-1 versus αPD-1 + corin-treated B16-F10 melanomas showing enrichment for cytokine activity, leukocyte migration in inflammation, antigen response, and immune response in the αPD-1 + corin–treated tumors.

Given our finding of corin-induced expression of immunogenic neoantigens, we sought to evaluate corin treatment of melanoma in an immune competent mouse model in conjunction with immune checkpoint blockade (ICB) in an immune “cold” melanoma model. The B16-F10 melanoma mouse model was established with treatment arms including vehicle, αPD-1, corin, and the combination of αPD-1 + corin (Figure 8A) and tumors were measured starting 7 days after inoculation. Remarkably, the combination α-PD-1 + corin treatment was found to decrease tumor growth by 66% as measured by tumor volume and tumor weight compared with αPD-1 treatment alone within 1 week of initiating therapy (Figure 8, B–D). Additionally, there were no significant changes in body weight (Figure 8E) or spleen weight (Figure 8F), suggesting that corin treatment is tolerated at the treatment dose. In order to assess the effect of corin treatment on the immune microenvironment, we isolated CD45^+^ cells from αPD-1 and αPD-1 + corin tumors and evaluated them by single-cell RNA-Seq (scRNA-Seq) (Figure 8, G–L, and Supplemental Figure 11, C–F). In total, 23,000 tumor-enriched CD45^+^ cells were profiled, analyzed by uniform manifold approximation and projection (UMAP), and segregated into 17 clusters based on the expression of leukocyte-associated genes and enrichment of gene signatures from external single-cell datasets (Figure 8, G–J) (63). This process allowed us to define major canonical T cell phenotypes such as naive T cells (Tn), exhausted T cells (Tex), CD8^+^ cytotoxic T lymphocytes (CTLs), Tregs, and cycling cells (Tcyc) as well as monocyte/macrophage lineages, B cells, neutrophils, dendritic cells, and plasma cells (Supplemental Figure 11, C and D). Strikingly, T cells from the αPD-1–treated tumors were primarily Tn cells (43%) and Tex (19%), whereas T cells from the corin + αPD-1–treated tumors underwent significant expansion of CTLs (57%), which supports induction of a CTL response by tumor-associated neoantigens (Figure 8, I and J, and Supplemental Figure 11E). Differential gene expression analysis in the T cell populations from αPD-1 versus αPD-1 + corin–treated tumors showed significant upregulation of granzyme B (*Gzmb*), IFN-γ (*Ifng*) and Cd8a along with significant downregulation of transcription factor 7 (*Tcf7*), lymphoid enhancer binding factor 1 (*Lef1*), and L-selectin (*Sell*) (Figure 8K and Supplemental Figure 11F). GSEA demonstrated enhanced cytokine activity, leukocyte migration inflammatory response, antigen response, and tumor-associated immune response in T cells isolated from αPD-1 + corin–treated tumors versus αPD-1 treatment alone (Figure 8L), consistent with T cell activation in response to tumor-associated neoantigen expression, while staining of tumors for CD3^+^ and CD8^+^ T cells showed increased CD8^+^ cells in the αPD-1 + corin–treated tumors versus αPD-1 treatment alone (Supplemental Figure 11G).

## Discussion

Regulation of RNA splicing is critical across all cell types to maintain normal biological functions including cell growth and differentiation (63, 64), while perturbations in splicing are widely seen in cancers (65). Here we establish a role for the CoREST complex in mediating tumorigenesis via direct interactions with the RNA splicing machinery as demonstrated by proteomic analysis, biochemical studies, and cryo-EM structural data. Furthermore, we define a role for corin in inhibiting these interactions and promoting alternative splicing in tumor cells. Remarkably, we find that the CoREST complex modulates pre-mRNA splicing via a variety of mechanisms including direct binding to the 3′ splice site recognition factor, U2AF2, and enhanced splicing factor transcription, both of which are inhibited by corin. Importantly, we define the region of binding of LSD1/RCOR1 to U2AF2 as being within the RRM domain of U2AF2 (66, 67), which interfaces with the DNA binding domain of LSD1 (47), suggesting potential competing affinities for LSD1 at this site for transcriptional versus splicing-associated regulatory functions. While we did not include RNA in our cryo-EM sample, the position of the RRM2 domain of U2AF2 could also accommodate bound RNA if it binds to RRM2 in a similar manner as depicted in the AlphaFold prediction. Additionally, we find both LSD1 and RCOR1 as well as splicing factors U2AF2 and SRSF1 to be located at critical splice junctions of tumor-associated splice variants and that these interactions are all inhibited in the setting of corin, further supporting functional interactions of splicing factors and the CoREST complex in cancers; conversely, U2AF2 does not significantly impact LSD1 demethylase activity or LHC deacetylase activity.

Our evaluation of CoREST complex effects on pre-mRNA splicing activity allowed us to identify significant corin-induced ASEs across melanomas regardless of tumor subtype, with the greatest influence on skipped exon and RI activities. Although such effects of chromatin remodeling enzymes, and HDACs in particular, have been identified in the past and largely been attributable to kinetic coupling of transcription to splicing changes (30), our PRO-Seq analysis of corin-treated cells revealed that there were no changes in Pol II kinetics. This suggests that corin-induced ASEs are the result of direct effects on splicing factor transcription and interactions of the CoREST complex with the splicing machinery, rather than splicing alterations related to Pol II rate changes. Remarkably, we find that corin-induced splicing changes are not unique to melanoma, as common significant pathways are also seen in ATRT and breast cancers, as identified through bioinformatic analyses, suggesting that corin-induced splicing differences may be more generally applicable to a large number of cancers. Additionally, we demonstrate that splicing events induced by corin lead to the expression of neopeptides that elicit an immune response in vitro, while corin treatment of melanoma in an immune-competent mouse model leads to significant inhibition of tumor growth in conjunction with ICB, rendering an immune-cold melanoma sensitive to checkpoint inhibition. Collectively, these data suggest the potential significant and broad application of corin to enhance immune-mediated responses to cancers.

While previous investigations have demonstrated the utility of pharmacologic induction of RNA splicing to promote neoantigen expression and anti-tumor immunity in preclinical studies (15, 16), and several drugs targeting the splicing machinery have been evaluated in clinical trials (5, 68), the vast majority of these reagents have not progressed in clinical development due to limited efficacy as single agents (5, 69, 70). Our identification of a role for the CoREST complex in binding to splicing factors and the use of the small molecule CoREST inhibitor, corin, to promote ASEs in cancer leading to neoantigen expression and T cell–mediated immunity represents a potential approach to promote immunoreactive neoantigen expression in immune-cold tumors. Indeed, studies suggest a link between tumor mutational burden (TMB), the presence of neoantigens on MHC molecules and responses to ICB therapy in cancers (71–74). We were therefore interested to see that corin induced thousands of ASEs in both a high-TMB tumor (melanoma) as well as a low-TMB tumor (ATRT), suggesting that corin treatment of ATRT may also result in significant neoantigen expression and induced tumor immunity. We also note that corin induced ASEs at critical splice junctions to a greater extent than either HDACi (MS-275) or LSDi (GSKLSD1) alone or in combination, suggesting improved efficacy of corin versus either agent not only on tumor growth (36), but also within the context of induced splicing changes. Indeed, a previous study found that MS-275 promotes induction of tumor immune editing of tumor neoantigens and tumor immune responses in combination with ICI with αPD-1 therapy in bladder cancer (75); however, the doses of MS-275 required to elicit these responses were significantly greater than those used for corin in this study. Importantly, we find that CoREST-regulated splicing factors are overexpressed across many tumor types compared with their respective normal tissues, suggesting that cancer cells are more vulnerable to corin-induced splicing changes due to tumor-associated functions of splicing factors. Given the specific targeting of the CoREST complex by corin and the lack of appreciable in vivo toxicity of corin seen in this study and others (36, 37), we suggest that this work provides a strong preclinical rationale for the use of CoREST inhibitor therapies in combination with ICIs for melanoma and other cancers. Although our findings highlight key tumor-intrinsic effects of corin, future in vivo studies should investigate its influence on tumor-extrinsic mechanisms to gain a more comprehensive understanding of how CoREST inhibition shapes tumor-autonomous effects.

**Figure 9:**
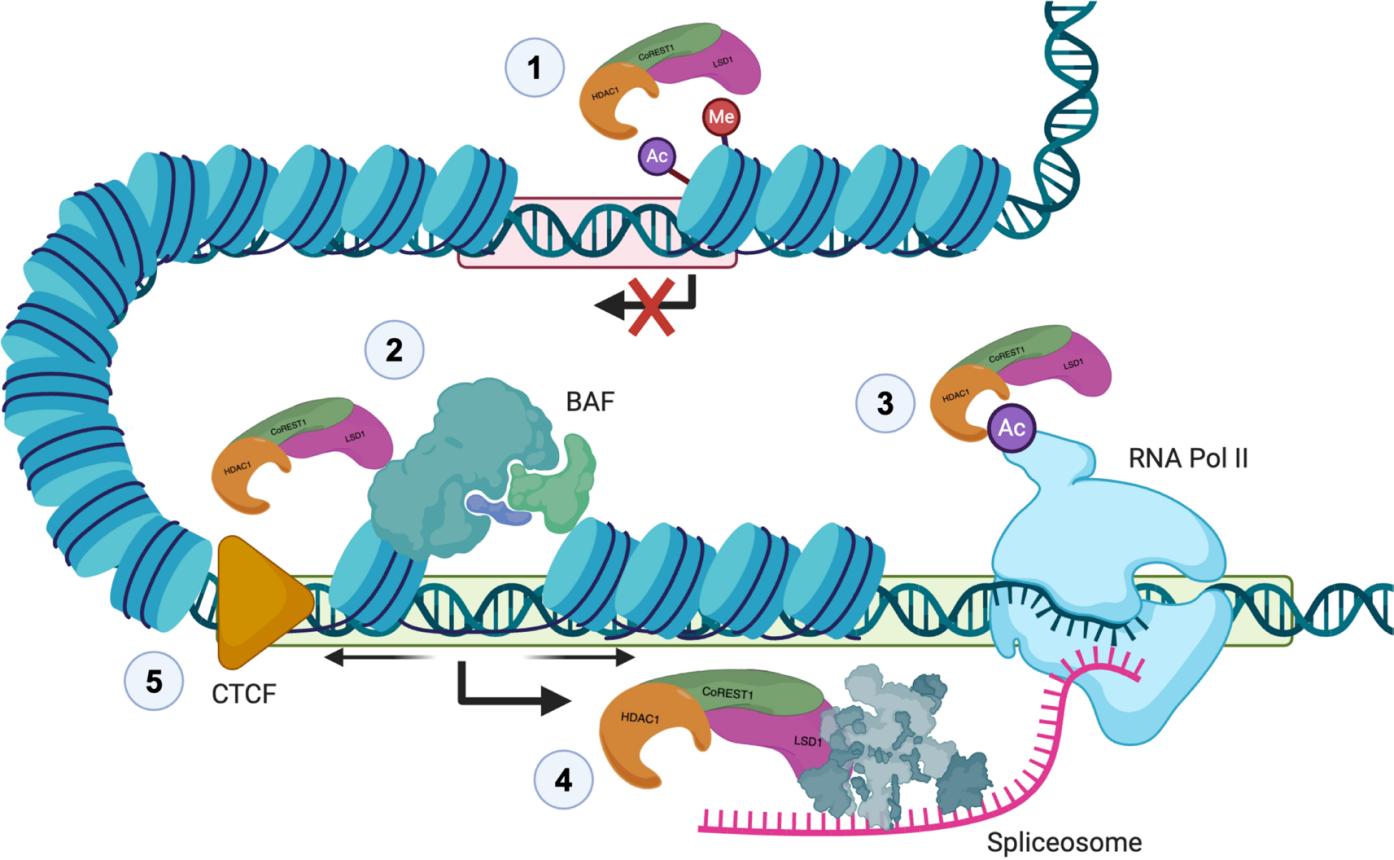
Proposed model for multilayered CoREST complex functions in epigenetic regulation, transcription, splicing, and chromatin architecture. Illustration of CoREST functions and layers of epigenetic crosstalk which ensure transcript fidelity at transcriptionally repressed (red box) and active (green box) regions. 1. Canonical function of CoREST in epigenetic repression through removal of active marks from histone tails. 2. CoREST interactions with the nucleosome-remodeling complex, BAF, promote open chromatin structure at transcriptionally active sites. 3. CoREST deacetylation of Pol II CTD affects transcriptional kinetics. 4. CoREST interactions with the pre-mRNA splicing machinery affects RNA splicing, alternative transcript expresson, and the development of neoantigens. 5. CoREST interactions with CTCF and regulation of higher-order chromatin structure.

Our finding of direct and functionally significant interactions between CoREST and the splicing machinery represents one of the first reports of direct interactions between a histone modifying complex and the splicing machinery. Furthermore, our cryo-EM studies represent, to our knowledge, the first reported structure of a histone modifying complex bound to a splicing factor. Given our findings of CoREST complex interactions with the RNA splicing machinery and its specific regulation of transcription and splicing of pre-mRNA splicing factors — along with previous findings of CoREST complex–mediated posttranslational modification of RNA Pol II (35), known interactions with transcription factors including SNAG-domain proteins (76) and REST, and the BAF complex (42) and CTCF— we propose the following model for CoREST complex regulation of transcription that allows for numerous interacting mechanisms to ensure transcript fidelity, which are disrupted in cancer (Figure 9). We suggest that the CoREST complex plays a critical role in ensuring coordinated regulation of chromatin accessibility, transcription factor binding to relevant DNA sites, transcription kinetics, and RNA splicing and that the CoREST complex provides a means of crosstalk between these systems to ensure proper transcript expression and timing. While we demonstrate the significance of these interactions here in the context of tumorigenesis, we expect these higher order regulatory functions of CoREST, and likely other epigenetic complexes, will prove significant within broader biological processes and provide a critical framework for the tightly coordinated epigenetic control of gene expression.

## Methods

### Sex as a biological variable.

This study exclusively examined female mice. It is unknown whether the findings would be similar for male mice, although we would not expect significant differences.

### Animal studies.

Six- to 10-week-old female C57BL/6 mice (Jackson Lab) were inoculated with 2.5 × 10^5^ B16F10 cells. Mice were randomly assigned treatment groups (vehicle control, anti–PD-1, corin, and corin + anti–PD-1) and treated with 200 μg/mice of corin (HY-111048, MedChemExpress) or 200 μL vehicle control (5% DMSO/PBS) by daily intraperitoneal (i.p.) injections starting from day 6 after the tumor injection. For anti–PD-1 treatment, mice were treated with 150 μg/mice anti–PD-1 (clone 29F.1A12, BioLegend, 135248) or isotype control (clone RTK2758, BioLegend, 400566) 3 times/week starting from day 7 posttumor grafts. Treatments occurred blinded and 10 mice per treatment group were used. Tumors were measured 3 times/week and volumes were calculated using the formula V = [1.58π × (length × width)3/2]/6 (77).

### Tissue processing.

Minced tumor biopsies were incubated with 200 μL 20 mg/mL collagenase, 200 μL 20mg/mL hyaluronidase, and 5 μL DNase for 20 minutes to 1 hour with agitation until the tumor fully dissociated. Single-cell suspension was passed through a 100 μm strainer and washed with 1X PBS by centrifugation. Cells were resuspended in 1ml of freezing media frozen down.

### Cell lines.

Melanoma cell lines 451Lu, 1205Lu, SKMEL24, WM793, and SKMEL28 were obtained from Meenard Heryln (The Wistar Institute). SKMEL5 cells were obtained from Deborah Lang (Boston University). Cells were maintained at 37°C at 5% CO_2_ and cultured in DMEM (Invitrogen, Thermo Fisher Scientific) supplemented with 1% penicillin/streptomycin, L-glutamine (2 mM), and 10% FBS. B16F10 cells were cultured in DMEM media (Gibco, 10566024) supplemented with 10% FBS and 100 U/mL of Pen/Strep (Gibco, 15070063). Cells were regularly tested negative for mycoplasma using MycoStrip - Mycoplasma Detection Kit (InvivoGen, rep-mysnc-50).

### Compounds.

Corin (no. HY-111048), MS275 (etinostat, no. HY-12163), and GSK-LSD1 (HY-100546A) were purchased from MedChemExpress. Stock solutions were made in DMSO and an equivalent volume of DMSO was used as a vehicle control.

### Cryo-EM data collection.

A cryo-EM dataset of truncated LSD1/RCOR1 complex bound to U2AF2 was collected at the Boston University Cryo-EM Core Facility using a 200 kV Thermo Fisher Glacios 2 cryoEM microscope equipped with a Falcon 4i direct electron detector and Selectris energy filter. A dataset of 12,845 exposures was collected in counting mode and recorded in Electron Event Representation (EER) format using a magnification of 130 kx, pixel size of 0.90 Å, nominal dose of 50 electrons (e-)/Å^2^, dose rate of 10.84 electrons per pixel per second (e-/px/s), a defocus range of –1.0 to –2.5 μm, and an energy filter slit width of 10 eV. A multishot imaging strategy was used to collect 3 shots per hole, utilizing beam image shift to move between each target.

### RNA-Seq.

Total RNA was extracted from melanoma cell lines treated with corin or vehicle control (DMSO) using the Qiagen RNeasy Mini Kit. Samples were quantified using nanodrop and sequenced by Azenta Inc.

### Melanoma splicing analysis.

Two tools were used to identify RNA splicing changes from RNA-Seq read data. rMATs-turbo (v.4.2.0) (52) was used to call skipped exon, alternative 3′SS, alternative 5′SS, and mutually exclusive events, while AltAnalyze (v.0.7.0.1) (53) was used to call intron retention events. rMATS was run using default parameters for BAM file inputs and the --allow-clipping parameter to prevent soft-clip skipping. A threshold of *q* < 0.05 and ΔPSI |0.1| was used to call significant differential events. AltAnalyze was run using default parameters for BAM file inputs. A threshold of *P* < 0.05 and ΔPSI |0.1| was used to call significant differential events. Gene ontology analysis and visualization was performed on significant SE, RI, and AP events using the R package enrichplot (v.1.22.0). Sashimi plots were generated using rmats2sashimiplot (v.2.0.4).

### RT-PCR splice gels.

Total RNA was extracted using the Qiagen RNeasy Mini Kit. cDNA was synthesized with the SuperScript cDNA Synthesis Kit (Thermo Fisher Scientific) and splice products were amplified with splice site-specific primers (Supplemental Table 10) and Taq polymerase for 35 cycles at Tm = 52.5C. DNA was run on 1.5% agarose gels and quantified using inclusion:exclusion product ratios.

### Neopeptide prediction analysis.

Two pipelines were used to predict splice-induced neopeptide candidates. First, JCEC files containing significant rMATS SE events (*q* < 0.05, ΔPSI ≥ |0.1|, TPM) ≥ 3, IJC + SJC ≥ 20) were input into SpliceTools (59) to translate and identify peptide sequences produced by frameshifted events. Then, sequences were digested into unique 8–11 mer sequences and filtered against the human proteome (University of California, Santa Cruz; Santa Cruz, California, USA) to isolate neopeptide products. Second, the SNAF (54) T-cell pipeline with default parameters was used to capture additional neopeptides produced from RI, AP, A5′SS, A3′SS, MXE, and trans-splicing events. Predicted neopeptides were validated against the human proteome and appended to the SpliceTools-identified events. Then, MHC binding predictions were performed using both HLAthena (61) and NetMHCpan (v.4.1) (60) with the following parameters “–l 8, 9, 10, 11” and %Rank ≤ 2. Neopeptides identified by both prediction tools were selected and ranked based on a score defined by the product of the -(%Rank), junction count, and |ΔPSI| value.

### Candidate neoantigen identification.

Candidate neoantigens were defined based on 2 methods: (a) a neopeptide was predicted to bind to human MHC and was identified in at least one corin-treated replicate of MS (FDR < 0.05) but not in either DMSO-treated replicate (b) a neopeptide was predicted to bind human MHC, had a high rank score (based on ΔPSI, junction count, and binding %Rank, and a high immunogenicity score from DeepImmuno (62) but was not recovered by MS as previously described (15, 16). C03:04 does not have adequate training data to accurately predict the immunogenicity of bound peptides so only highly ranked A11:01 and B40:01 were tested.

### ELISpot assay.

To expand reactive T cells, HLA-matched PBMCs were prestimulated with synthesized peptides (10 μg/mL) for 14 days in IL2/IL7 media. APCs were isolated from CD4 and CD8 depleted PBMCs, loaded with peptides (10 μg/mL), and seeded at a ratio of 3:1 with the prestimulated T cells in a 96 well ELISpot plate. IFN-γ ELISpot was performed per manufacturer’s instructions and spots were quantified using an Immunospot Analyzer.

### scRNA-Seq sample preparation.

Cryopreserved single-suspension tumor samples were thawed, and dead cells were removed using the Dead Cell Removal Kit (Miltenyi, 130-090-101) following manufacturer instructions. Live cells from the flow-though were used for CD45^+^ cell isolation using CD45 mouse MicroBeads (Miltenyi, 130-052-301) following the recommendations. CD45^+^ cells were counted and resuspended in PBS + 0.04% BSA at 1,000 cells/μL for scRNA-Seq.

### Statistics.

Results were considered significant for an adjusted *P* value of less than 0.05. Data are presented as the mean ± SD and are representative of at least 2 independent experiments. Tests used to determine statistical significance are noted in the figure legends and include an unpaired, 2-tailed *t* test, a Mann-Whitney *U* test, 1-way ANOVA with Tukey’s test, Holm-Šídák’s multiple-comparison test, or a 2-way ANOVA with Tukey’s test.

### Study approval.

Mice were maintained under pathogen-free conditions in an American Association for Accreditation of Laboratory Animal Care–accredited (AAALAC-accredited) facility at the Dana Farber Cancer Institute, under the supervision of the Laboratory Animal Science Center (LASC) and its staff of veterinarians and support personnel (IACUC protocol no. 17-014).

### Data availability.

Raw data, processed data, and metadata from the bulk RNA-Seq (GSE280449), scRNA-Seq (GSE280450), and PRO-Seq (GSE280448) experiments have been deposited in the NCBI’s Gene Expression Omnibus (GEO) database. Raw MS data for the CoREST IP-MS are available on PRIDE (project no. PXD056700, Token: MxX17vDulsT2) and neopeptide IP-MS (MHC associated peptides) are available on the MassIVE public repository (https://massive.ucsd.edu/) under accession code MSV000096071. All code used for analysis of sequencing were deposited on GitHub (https://github.com/robertfisher002/CoREST_Splicing; branch main; commit ID 92ac17b). Cryo-EM map and model were deposited in the Protein Data Bank (PDB), EMPAIR database, and Electron Microscopy Data Bank (EMDB) under the following accession codes: PDB, 9DWU; EMPAIR, 12854; EMDB, 47265). Raw cryo-EM movies will be uploaded to the EMPIAR database. All raw data values can be found in the Supporting Data Values files. Additional details are available in the Supplemental Methods.

## Author contributions

Conceptualization was contributed by RMA and RJF. Methodology was contributed by RMA, RJF, K Park, KL, K Pinjusic, AV, CSE, PF, SBF, JAM, HJ, EN, S Stranksy, AF, JDC, MAA, CWH, DBK, S Sidoli, CJW, and PAC. Investigation was contributed by RJF, K Park, KL, K Pinjusic, HJ, EN, AF, AG, ER, CWH, SD, and S Sidoli. Visualization was contributed by RJF, KPark, CWH, and SSidoli. Funding acquisition was contributed by RMA and PAC. Project administration was contributed by RMA. Supervision was contributed by RMA, PAC, CJW, S Sidoli, and DBK. Writing of the original draft was contributed by RMA and RJF. Review and editing were contributed by RJF, K Park, KL, K Pinjusic, AV, CSE, SBF, JAM, JDC, AG, ER, HJ, EN, AF, CWH, DBK, S Sidoli, CJW, PAC, and RMA

## Funding support

This work is the result of NIH funding, in whole or in part, and is subject to the NIH Public Access Policy. Through acceptance of this federal funding, the NIH has been given a right to make the work publicly available in PubMed Central.

- Department of Defense Grant W81XWH2110980 (RMA, KP)
- Melanoma Research Alliance Grant #1045461 (RMA, RJF)
- American Heart Association (Postdoctoral Fellowship Award 826614 (KL)
- NIH MIRA grant R35-GM147254 (AF)
- The Hevolution Foundation (AFAR), the Einstein-Mount Sinai Diabetes Center, and the NIH Office of the Director (S10OD030286) (SS)
- U01 CA243004 and T32 AI007309-33 (CSE)
- The Swiss National Science Foundation (Grant 225660) and NIH 1R01CA279391-01A1 (KP)
- National Institutes of Health grant (R01 CA233800) and the Masschusetts Life Science Center (JAM)
- National Institutes of Health grant (GM149229) (PAC)
- National Institutes of Health grants (U54-CA272688, RO1-HL157174, RO1-CA285308 & RO1-CA279391) (DBK)
- NIH U24CA224331 (to CJW)
- NIH grant S10OD032253 to the Boston University CryoEM Core Facility

## Supplementary Material

Supplemental data

Unedited blot and gel images

Supplemental tables 1-10

Supporting data values

## Version 1 12/09/2025

In-Press Preview

## Version 2 01/23/2026

Electronic publication
