# Supplementary material for: CoREST complex inhibition alters RNA splicing to promote neoantigen expression and enhance tumor immunity
Source: JCI Insight. 2025 Dec 9;11(2):e190287. doi: 10.1172/jci.insight.190287 (PMC12892918; doi:10.1172/jci.insight.190287)
Supplement: Supplemental data [file jciinsight-11-190287-s073.pdf]

Supplementary Methods

Histology and immunofluorescence

Tissue samples were embedded in optimal cutting temperature (OCT) compound. Sections of 5 μm thickness were prepared and mounted onto glass slides. Sections were fixed in ice-cold 100% methanol for five minutes and subsequently washed three times for three minutes each in 1× DPBS containing 0.1% Tween-20. Sections were incubated for 1 hour in a blocking buffer made up of 5% goat serum at room temperature. Slides were then incubated with primary antibodies, including anti-CD3 (Origene, SM1754P, Rockville, MD, USA) and anti-CD8 (Bio X cell, BE0117, Lebanon, NH, USA) overnight at 4°C. The following day, the slides were washed three times in 1x DPBS with 0.1% Tween-20 and incubated with fluorochrome-conjugated secondary antibodies at room temperature for two hours. Slides were mounted using one drop of Fluoroshield with DAPI mounting medium (F6057, Sigma-Aldrich, USA). Images were acquired using the Axio Scanner 7 (Zeiss, Jena, Germany) and analyzed with Zen image processing software (Zeiss).

Protein purification

The CoREST complex was purified as described (Jay H. Kalin et al., 2018) (1). Briefly, the constructs of full-length LSD1, full-length HDAC1 and FLAG-tagged NΔ85-CoREST1 were cloned into pcDNA3.0-based vectors and transiently transfected into HEK293F suspension cells using branched polyethylenimine. After 48 hours of incubation, cells were harvested, lysed in cold lysis buffer (50 mM Tris–HCl [pH 7.5], 50 mM KCl, 5% glycerol, 0.4% Triton X-100, 1X Roche EDTA-free Complete Protease Inhibitor cocktail), and sonicated. Insoluble fractions were removed by centrifugation, and the complex was purified via FLAG affinity chromatography. After washing, the complex was cleaved with TEV protease and further purified by Size Exclusion Chromatography using a Superose 6 10/300 GL column (GE Healthcare). For GST-tagged U2AF2 and SRSF1, constructs were cloned into pGEX6P1 vector, expressed in *Escherichia coli*. Cells were harvested and resuspended in GST binding buffer (20mM Tris [pH8.0], 0.2mM EDTA,

1M NaCl, 10% Glycerol, 0.1% NP-40, 1mM PMSF), and lysed using a french press. The lysate was cleared by centrifugation, and the supernatant was incubated with glutathione-Sepharose 4B resin (Cytiva) for 2 hours at 4°C. The resin was extensively washed with GST binding buffer, and the GST-tagged proteins were eluted with GST binding buffer containing 30 mM glutathione. The eluted proteins were dialyzed against dialysis buffer (20mM Tris [pH8.0], 0.2mM EDTA, 150mM NaCl, 10% Glycerol), then stored at -80°C. For His-tagged fl-LSD1, truncated LSD1/RCOR1 complex (2) and U2AF2 2 a.a. 41-471, constructs were cloned into a pET15b vector (His-tagged fl-LSD1, truncated His-tagged LSD1, and U2AF2) and pET-28b (His-tagged truncated RCOR1). The plasmid encoding His-tagged fl-LSD1 was a kind gift from Dr. Yang Shi (University of Oxford). For the purification of fl-LSD1, truncated LSD1/RCOR1 complex and U2AF2 (aa241-471), His binding buffer (20mM Tris [pH 8.0], 300mM NaCl, 1mM TCEP) was used for cell resuspension, while the resin was washed with His binding buffer containing 40 mM imidazole, and elution was performed using His binding buffer containing 200 mM imidazole. The eluants were dialyzed against the His binding buffer containing 1 mM EDTA. For His-LSD1, additional purification was performed using MonoQ (Cytiva) and Superdex 200 increase 10/300 GL columns (Cytiva). Fluorescent dye labeling of His-U2AF2 fragments was performed using the MO-L011 Protein Labeling Kit RED-NHS 2nd Generation (Nanotemper) according to the manufacturer's instructions.

Co-Immunoprecipitation

Cells were treated with DMSO or Corin to a final concentration of 2.5μM and incubated for 24 hours. After harvesting in PBS, cell extracts were prepared by resuspending the cell pellet in IP buffer (20 mM Tris [pH 7.5], 137 mM NaCl, 1 mM $CaCl_2$, 1% NP-40, 10% glycerol, 1 mM $MgCl_2$, 1X protease and phosphatase inhibitor cocktail; ThermoFisher Scientific, and 0.0125 U/ml Benzonase; Sigma-Aldrich) and rotating at 4°C for 1 hour. Protein concentration was measured using a BCA assay. ProteinA-Dynabeads (Invitrogen, 30 μl per sample) were incubated with

antibodies (**Supplementary Table 9**) at 4°C for 1 hour and washed with blocking buffer (0.5% BSA in PBS). 1mg of cell extract were added to antibody-bound beads and incubated overnight at 4°C. After extensive washing with IP buffer lacking $MgCl_2$, protease and phosphatase inhibitor cocktail, and Benzonase, immunoprecipitated proteins were eluted, resolved by SDS-PAGE and subjected to immunoblot analyses.

***In vitro* pull-down assay**

GST-tagged splicing factors (final concentration 300 nM) were mixed with the CoREST complex or LSD1 (final concentration 15 nM), 15 µl of glutathione-Sepharose 4B resin (Cytiva), and BSA (final concentration 0.3 mg/ml) in binding buffer (20 mM Tris [pH 8.0], 0.2 mM EDTA, 150 mM NaCl, 10% glycerol, 0.1% NP-40, 1 mM PMSF) to a total volume of 300 µl. The mixtures were incubated with rotation at 4°C for 2 hours, after which the resin was washed four times with binding buffer. The resin-bound proteins were eluted, resolved by SDS-PAGE and subjected to immunoblot analyses.

S-trap Protein Digestion

Immunoprecipitates from two biological replicates were eluted in a buffer containing 5% SDS, 5 mM DTT and 50 mM ammonium bicarbonate (pH = 8), and left on the bench for about 1 hour for disulfide bond reduction. Samples were then alkylated with 20 mM iodoacetamide in the dark for 30 minutes. Afterward, phosphoric acid was added to the sample at a final concentration of 1.2%. Samples were diluted in six volumes of binding buffer (90% methanol and 10 mM ammonium bicarbonate, pH 8.0). After gentle mixing, the protein solution was loaded to an S-trap filter (Protifi) and spun at 500 g for 30 sec. The sample was washed twice with binding buffer. Finally, 1 µg of sequencing grade trypsin (Promega), diluted in 50 mM ammonium bicarbonate, was added into the S-trap filter and samples were digested at 37°C for 18 h. Peptides were eluted in three steps: (i) 40 µl of 50 mM ammonium bicarbonate, (ii) 40 µl of 0.1% TFA and (iii) 40 µl of 60% acetonitrile

and 0.1% TFA. The peptide solution was pooled, spun at 1,000 g for 30 sec and dried in a vacuum centrifuge. Prior to mass spectrometry analysis, samples were desalted using a 96-well plate filter (Orochem) packed with 1 mg of Oasis HLB C-18 resin (Waters). Briefly, the samples were resuspended in 100 µl of 0.1% TFA and loaded onto the HLB resin, which was previously equilibrated using 100 µl of the same buffer. After washing with 100 µl of 0.1% TFA, the samples were eluted with a buffer containing 70 µl of 60% acetonitrile and 0.1% TFA and then dried in a vacuum centrifuge.

Microscale thermophoresis

Fluorophore-labeled U2AF2 fragments (10 nM) were titrated with the truncated LSD1/RCOR1 complex in a 1:1 mixture of ligand buffer (20 mM Tris [pH 8.0], 300 mM NaCl, 1 mM TCEP) and U2AF2 buffer (20 mM Tris [pH 8.0], 200 mM NaCl, 1 mM TCEP, 2 mg/ml BSA, 0.1% Tween-20) at 25°C. The truncated LC was prepared at concentrations up to 44 µM using a twofold serial dilution. Fluorophore-labeled U2AF2 was then added, thoroughly mixed, and loaded into Monolith NT.115 Premium Capillaries (NanoTemper). MST assays were conducted using a Monolith NT.115 pico (NanoTemper) in pico-RED mode. The excitation laser power was set to 8% for U2AF2 85-471 and 10% for U2AF2 241-471, while the MST power was set to Medium. K_D values were determined using MO.analysis (v2.3) software with a quadratic equation binding K_D model.

GST-LSD1 for demethylase assay

GST-tagged LSD1 (aa 171–852) was subcloned in a pGEX6P-1 vector and bacterially expressed and purified as previously reported (2,3). BL21-CodonPlus (DE3)-RIPL competent cells (Agilent) were transformed with the GST-LSD1 plasmid, followed by the inoculation in LB media containing 100 mg/L ampicillin and 35 mg/L chloramphenicol. When A_{600} reached 0.6 at 37 °C, 0.5 mM IPTG was added to the media to induce protein production at 16 °C for 20 h. Cells were harvested via centrifugation at 2,702*g* and resuspended in the lysis buffer (20 mM

Na_2HPO_4, 3.6 mM KH_2PO_4, 5.4 mM KCl, 280 mM NaCl, 1 mM EDTA, 10 mM DTT and 10% glycerol at pH 7.4). Resuspended cells were lysed by french press and were further centrifuged at 20,853*g* for 30 min at 4 °C. The supernatant was then incubated with glutathione agarose resin (MCLAB) and washed with the lysis buffer. GST-LSD1 protein was eluted with the elution buffer (lysis buffer containing 20 mM glutathione). The eluant was then concentrated and buffer-exchanged against the lysis buffer containing 1 mM β-mercaptoethanol (BME) instead of 10 mM DTT, using a 50-kDa MWCO concentrator (Amicon, Millipore Sigma). Final GST-LSD1 was approximately 70% pure, as assessed by SDS-PAGE and Coomassie staining. GST-LSD1 protein solutions were aliquoted, flash-frozen, and stored at −80 °C.

His_6-tagged U2AF2 (aa 85-471 and 241-471) for enzymology

His_6-tagged U2AF2 (aa 85-471 and 241-471) was subcloned in a pET-15b vector and bacterially expressed and purified. LOBSTR Rosetta DE3 competent cells were transformed with the His_6-tagged U2AF2 plasmids, followed by inoculation in LB media containing 100 mg/L ampicillin and 35 mg/L chloramphenicol. When A_{600} reached 0.6 at 37 °C, 0.5 mM IPTG was added to induce protein production at 16 °C for 20 h. Cells were harvested via centrifugation at 2,702*g* and resuspended in the lysis buffer (20 mM Tris at pH 7.8, 200 mM NaCl, and 0.5 mM TCEP). Resuspended cells were lysed by french press and centrifuged at 20,853*g* for 30 min at 4 °C. The supernatant was then incubated with the nickel agarose beads (MCLAB) and the beads were washed with the lysis buffer supplemented with 20 mM imidazole. His_6-U2AF2 (aa 85-471 and 241-471) was eluted with the elution buffer (lysis buffer containing 200 mM imidazole). The eluant was then concentrated down using a 10 kDa MWCO concentrator (Amicon, Millipore Sigma) and further purified by size exclusion chromatography on a Superdex 200 Increase 10/300 GL column (Cytiva) pre-equilibrated with lysis buffer. Fractions corresponding to the monomeric peak were >90% pure, as determined by SDS-PAGE and Coomassie staining.

Purified protein was pooled, concentrated using a 10 kDa MWCO concentrator, aliquoted, flash-frozen in liquid nitrogen, and stored at −80 °C.

H3 peptide demethylase assays

Demethylation of the H3 peptide substrate by GST-LSD1 was measured using a horseradish peroxidase (HRP)-coupled assay, as previously described (1,2). Briefly, 35 μM H3K4me2 peptide (aa 1–21) was added to demethylase assay buffer containing 50 mM HEPES (pH 7.5), 0.1 mM 4-aminoantipyrine, 1 mM 3,5-dichloro-2-hydroxybenzenesulfonic acid, 0.04 mg/mL HRP (Worthington Biochemical), 15 μM $U2AF2_{241\text{-}471}$ (when included), and 200 nM GST-LSD1. Reactions were carried out at 23 °C. Absorbance at 515 nm was recorded every 15 seconds over a 20 minute time course, and product formation was quantified using an extinction coefficient of 26,000 M^{-1} cm^{-1}. The steady-state region between 2 and 4 minutes was used for rate calculations. Data were analyzed using GraphPad Prism 10 with an unpaired, two-tailed Student's t-test, testing the null hypothesis that LSD1 activity does not differ in the presence or absence of $U2AF2_{241–471}$. Three technical replicates were performed for each condition.

Nucleosome deacetylation assays

H3K9ac 147-bp nucleosomes (100 nM) were treated with the LHC complex (40 nM) with or without U2AF2 (10 μM) in a reaction buffer [50 mM HEPES, pH 7.5, 100 mM KCl, 100 μM inositol hexaphosphate (IP6), 0.2 mg/mL BSA]. The protein samples were incubated for 0, 30, 60, 90, and 120 min at 37 ˚C, and at each time point, 6.5 μL aliquots were quenched with an equivalent amount of a quenching buffer [20 mM EDTA, 2X SDS loading dye]. The samples were boiled at 95 ˚C for 3 min and resolved on 4-20% SDS-PAGE pre-cast Tris-glycine gels (Thermo Fisher Scientific). Proteins were then transferred to nitrocellulose membranes (Transfer Stack, Invitrogen, IB301031) using the iBlot (Invitrogen) transfer system with P3 (20 V)

for 5 min, followed by blocking in 5% BSA in TBST buffer [20 mM Tris, 150 mM NaCl, 0.1% Tween, pH 7.5] for 1 h at room temperature. The membrane was incubated overnight at 4 ˚C with primary antibodies [anti-H3K9ac (1:2,000, Abcam, ab32129); anti-H3 (1:2000, Abcam, ab1791)]. After washing with TBST five times (2 min each), the horseradish peroxidase (HRP)-conjugated anti-rabbit IgG secondary antibody (1:2000) in TBST was added to the membrane and incubated for 1 h at room temperature. The membrane was visualized by ECL substrate (Bio-Rad) and G: BOX mini gel imager (Syngene). The bands on the membrane were quantified using Image J software, and the intensities were normalized by the intensity at T=0. Data analyses were conducted with GraphPad Prism 10, and the data were fit to a single-phase exponential decay curve (GraphPad Prism 10, with constrains Y0 = 1 and plateau = 0).

LC-MS/MS Acquisition and Analysis

Samples were resuspended in 10 µl of 0.1% TFA and loaded onto a Dionex RSLC Ultimate 300 (Thermo Scientific), coupled online with an Orbitrap Fusion Lumos (Thermo Scientific). Chromatographic separation was performed with a two-column system, consisting of a C-18 trap cartridge (300 µm ID, 5 mm length) and a picofrit analytical column (75 µm ID, 25 cm length) packed in-house with reversed-phase Repro-Sil Pur C18-AQ 3 µm resin. Peptides were separated using a 90 min gradient from 4-30% buffer B (buffer A: 0.1% formic acid, buffer B: 80% acetonitrile + 0.1% formic acid) at a flow rate of 300 nl/min. The mass spectrometer was set to acquire spectra in a data-dependent acquisition (DDA) mode. Briefly, the full MS scan was set to 300-1200 m/z in the orbitrap with a resolution of 120,000 (at 200 m/z) and an AGC target of 5x10e5. MS/MS was performed in the ion trap using the top speed mode (2 secs), an AGC target of 1x10e4 and an HCD collision energy of 35. Proteome raw files were searched using Proteome Discoverer software (v2.5, Thermo Scientific) using SEQUEST search engine and the SwissProt human database. The search for total proteome included variable modification of N-terminal acetylation,

and fixed modification of carbamidomethyl cysteine. Trypsin was specified as the digestive enzyme with up to 2 missed cleavages allowed. Mass tolerance was set to 10 pm for precursor ions and 0.2 Da for product ions. Peptide and protein false discovery rate was set to 1%. Following the search, data was processed as described (4). Briefly, proteins were log2 transformed, normalized by the average value of each sample and missing values were imputed using a normal distribution 2 standard deviations lower than the mean. Statistical regulation was assessed using heteroscedastic T-test (if p-value < 0.05). Data distribution was assumed to be normal but this was not formally tested.

Western blot

Whole-cell protein lysates were extracted in MPER buffer supplemented with Halt Protease Inhibitor. Samples were run on 10% SDS-PAGE gels and transferred to polyvinylidene difluoride membranes. Membranes were blocked with 5% nonfat dry milk diluted in a 0.05% Tween 20 PBS solution and incubated overnight at 4C with primary antibody (**Supplementary Table 9**). Membranes were washed 3x with Tris Buffered Saline with Tween 20 (TBST) then incubated with HRP-conjugated secondary antibodies (**Supplementary Table 9**) for 1 hour, then washed again 3x with TBST before visualized with ECL Western Blot Substrate (ThermoFisher Scientific). Chemiluminescent blots were imaged with the Chemi-doc XP Imager (Bio-Rad) and analyzed using ImageJ densitometry quantification. All bands were normalized against GAPDH for quantification.

Cryo-EM sample preparation

Frozen samples of truncated LSD1/RCOR1 complex (6xHis-LSD1 (aa171-852) + 6xHis-RCOR1 (aa289-485)) and 6xHis-U2AF2 (aa241-475) were thawed on ice, mixed at a molar ratio of 1:5. The protein mixture was dialyzed overnight at 4°C against a dialysis buffer (20 mM Tris [pH 8.0], 150 mM NaCl, and 1 mM TCEP). Following dialysis, the sample was separated using a Superdex

200 Increase 10/300 GL column (GE Healthcare). The peaks were analyzed by SDS-PAGE, and fractions containing LSD1, RCOR1, and U2AF2 were pooled and concentrated for grid freezing. Ultrafoil R1.2/1.3 Au 300 mesh grids were glow-discharged for 45 seconds at 15mA using a PELCO easiGLOW Glow Discharge System to apply a negative charge to their surface. Then, 3 µL of sample mixture at a concentration of 0.75 mg/mL was applied to the grid, immediately blotted for 3 seconds with a blot force of 3, and plunge-frozen in liquid ethane using a Vitrobot Mark IV apparatus (Thermo Fisher) set to 100% humidity and 4°C.

Cryo-EM data processing

The cryoEM dataset was processed in cryoSPARC v4.5.1 (5). Exposures were imported with an EER upsampling factor of 2 and cropped to one-half their original resolution using Patch Motion Correction. The CTF was corrected using Patch CTF Estimation, and poor-quality micrographs were removed yielding 6,118 high-quality micrographs. An initial set of blob picks from a small subset of micrographs was classified using 2D Classification to create a set of high quality 2D templates. The 2D templates were used to perform particle picking using Template Picker and Inspect Picks, then extracted at a box size of 360 pixels using Extract from Micrographs to yield an uncleaned particle stack (3,973,676 particles). Two rounds of 2D Classification with a circular mask of 150 Å, initial classification uncertainty factor of 4, number of online-EM iterations of 30, and Batchsize per class of 300, were performed to remove junk particles by discarding poor-quality 2D classes to yield a partially cleaned particle stack (1,183,288 particles). Two rounds of parallel multi-structure Ab-initio reconstruction jobs were performed where particles from distinctly good volumes were selected yielding a cleaned particle stack (961,750 particles). Homogenous Refinement with Adaptive Marginalization, Non-Uniform Refinement (6), and Local Refinement with pose/shift gaussian prior were performed consecutively to align the cleaned particle stack. Rebalance Orientations was performed on the aligned cleaned particle stack to remove particles from oversampled preferred orientations, yielding a balanced aligned cleaned particle stack

(695,785 particles). Focused 3D classification at a target resolution of 15 Å and 4 classes was performed using a spherical focus mask centered on the density corresponding to U2AF2. Homogenous Reconstruction Only was performed on all four particle stack corresponding to the 3D volume outputs. The reconstruction with the strongest U2AF2 density was selected as the final reconstruction of truncated LSD1/RCOR1 complex bound to U2AF2 (172,429 particles, 5.14 Å).

Cryo-EM model building

Initial model of truncated LSD1/RCOR1 complex bound to U2AF2 was built by rigid body fitting models of LSD1+CoREST (PDB: 2IW5) (7) and U2AF2 (PDB: 5W0H) (8) into the EM density map using ChimeraX (9). Hydrogen atoms and alternate side-chain rotamers were removed from the initial model using PyMOL. To account for missing EM density in the region of the RCOR1 protein, residues 377-440 were removed from RCOR1. Individual Ramachandran and rotamer outliers were corrected where applicable using Coot 0.9.6 (10), and the final model was validated using cryo-EM Comprehensive Validation module in PHENIX (11) running MolProbity (12). Figures were generated using ChimeraX (9).

RNA-seq data analysis

Paired-end RNA-seq reads (2x150bp) were quality and adaptor-trimmed using Trimmomatic (v.0.36) (13). Trimmed reads were mapped to the ENSEMBL reference human genome (GRCh38) using STAR (v.2.5.2b) (14). BAM files were generated for downstream splicing and differential gene expression analysis. Unique gene counts were calculated using featureCounts from Subread (v.1.5.2) (15) and differential expression analysis was performed using DESeq2 (v.1.44.0)(16) based on hit counts. The Wald test was used to produce log2 fold change and p-values between comparisons. A threshold of $p < 0.01$ and $LF > |0.5|$ was used to call significant

changes in gene expression. Gene set enrichment analysis was performed using GSEA (v.4.3.2) (17) with KEGG (18) and Hallmark (19) curated gene sets.

PRO-seq library construction

SKMEL5 cells were treated for 24h with 2.5μM corin or DMSO and permeabilized as described. All sample preparation was conducted on ice (4°C). Cells were washed in ice cold 1x PBS and resuspended in wash buffer (10 mM Tris-HCl pH 8.0, 10% glycerol, 250 mM sucrose, 10 mM KCl, 5 mM MgCl2, 0.5 mM DTT, 1mM EGTA, Halt protease inhibitor cocktail (Thermo Scientific), and 4 u/mL RNase inhibitor [SUPERaseIN, Invitrogen]). Then cells (2x10^7) were gently permeabilized in permeabilization buffer (10 mM Tris-HCl pH 8.0, 10% glycerol, 250 mM sucrose, 10 mM KCl, 5 mM MgCl2, 0.5 mM DTT, 0.1% Igepal, protease inhibitors cocktail (Roche), 4μ/mL RNase inhibitor [SUPERaseIN, Invitrogen]) for 5 minutes. Cells were recovered by centrifugation (400 x g for 8 minutes) and the supernatant was carefully removed. Cells were washed with 10 mL of wash buffer and then centrifuged again under the same conditions. Finally, cells were resuspended in 400μL of freeze buffer (50 mM Tris-HCl pH 8.0, 40% glycerol, 5 mM MgCl2, 0.5 mM DTT, 4 u/mL RNase inhibitor [SUPERaseIN, Invitrogen]) and stored at -80°C.

Aliquots of frozen (-80°C) permeabilized cells were thawed on ice and pipetted gently to fully resuspend. Aliquots were removed and permeabilized cells were counted using a Luna II, Logos Biosystems instrument. For each sample, 1 million permeabilized cells were used for nuclear run-on, with 50,000 permeabilized *Drosophila* S2 cells added to each sample for normalization. Nuclear run on assays and library preparation were performed essentially as described in Reimer et al. [K. A. Reimer, C. A. Mimoso, K. Adelman, K. M. Neugebauer, Molecular Cell (2021)] with modifications noted: 2X nuclear run-on buffer consisted of (10 mM Tris (pH 8), 10 mM MgCl2, 1 mM DTT, 300mM KCl, 20uM/ea biotin-11-NTPs (Perkin Elmer), 0.8U/μl SuperaseIN (Thermo),

1% sarkosyl). Run-on reactions were performed at 37°C. Random hexamer extensions (UMIs) were added to the 3' end of the 5' adapter and 5' end of the 3' adapter. Adenylated 3' adapter was prepared using the 5' DNA adenylation kit (NEB) and ligated using T4 RNA ligase 2, truncated KQ (NEB, per manufacturer's instructions with 15% PEG-8000 final) and incubated at 16°C overnight. 180µl of betaine buffer (1.42g of betaine brought to 10mL) was mixed with ligations and incubated 5 min at 65°C and 2 min on ice prior to addition of streptavidin beads. After T4 polynucleotide kinase (NEB) treatment, beads were washed once each with high salt, low salt, and 0.25X T4 RNA ligase buffer (NEB) and resuspended in 5' adapter mix (10 pmol 5' adapter, 30 pmol blocking oligo, water). 5' adapter ligation was per Reimer but with 15% PEG-8000 final. Eluted cDNA was amplified 5-cycles (NEBNext Ultra II Q5 master mix (NEB) with Illumina TruSeq PCR primers RP-1 and RPI-X) following the manufacturer's suggested cycling protocol for library construction. A portion of preCR was serially diluted and for test amplification to determine optimal amplification of final libraries. Pooled libraries were sequenced using the Illumina NovaSeq platform

PRO-sequencing data analysis

All custom scripts described herein are available on the AdelmanLab GitHub (https://github.com/AdelmanLab/NIH_scripts). Dual, 6nt Unique Molecular Identifiers (UMIs) were extracted from read pairs using UMI-tools [10.1101/gr.209601.116]. Read pairs were trimmed using cutadapt 1.14 to remove adapter sequences (-O 1 --match-read-wildcards -m {20,26}). The UMI length was trimmed off the end of both reads to prevent read-through into the mate's UMI, which will happen for shorter fragments. An additional nucleotide was removed from the end of read 1 (R1), using seqtk trimfq (https://github.com/lh3/seqtk), to preserve a single mate orientation during alignment. The paired end reads were then mapped to a combined genome index, including both the spike (dm6) and primary (hg38) genomes, using bowtie2 [10.1038/nmeth.1923]. Properly paired reads were retained. These read pairs were then

separated based on the genome (i.e. spike-in vs primary) to which they mapped, and both these spike and primary reads were independently deduplicated, again using UMI-tools. Paired-end RNA-seq reads were mapped to the hg38 reference genome via HISAT2 v2.2.1 (--known-splicesite-infile). To select gene-level features for differential expression analysis, and for pairing with PRO-seq data, we assigned a single, dominant TSS and transcription end site (TES) to each active gene. This was accomplished using a custom script, get_gene_annotations.sh (available at https://github.com/AdelmanLab/GetGeneAnnotation_GGA), which uses RNA-seq read abundance and PRO-seq R2 reads (RNA 5' ends) to identify dominant TSSs, and RNA-seq profiles to define most commonly used TESs. RNA-seq and PRO-seq data from all conditions were used for this analysis, to comprehensively capture gene activity in these samples.Reads were summed within the TSS to TES window for each active gene using the make_heatmap script (https://github.com/AdelmanLab/NIH_scripts), which counts each read one time, at the exact 3' end location of the nascent RNA. DEseq2, using the Wald test, was used to determine statistically significant differentially expressed genes. Unless otherwise noted, the default size factors determined by DEseq2 were used.

RNA immunoprecipitation coupled with quantitative PCR (RIP qPCR)

SKMEL5 cells were treated with DMSO or Corin to a final concentration of 2.5μM and incubated for 24 hours. Cells were harvested PBS, lysed in RNA lysis buffer (25 mM Tris [pH 7.5], 150 mM NaCl, 5 mM EDTA, 5 mM MgCl2, 1% NP-40, 0.5 mM DTT, 1X protease and phosphatase inhibitor cocktail; ThermoFisher Scientific, 40 U/ml RNase inhibitor; Applied Biosystems), and incubated for 30 minutes at 4°C with rotation. Chromatin was sheared by passing through a 22-gauge needle, and lysates were centrifuged. The supernatant was mixed with 1.5 mg of cell extract and specific antibodies (**Supplementary Table 9**) and incubated for 2 hours at 4°C. ProteinA Dynabeads (Invitrogen, 30 μl per sample) were washed and added to each sample for 2.5 hours, followed by three washes with RNA lysis buffer. The beads were then treated with TRIzol for RNA

extraction. RNA was purified by chloroform extraction and isopropanol precipitation, followed by DNase treatment and cDNA synthesis using the random hexamer and SuperScript cDNA Synthesis Kit (ThermoFisher Scientific). Quantitative PCR (qPCR) was performed using SYBR® Green Quantitative RT-qPCR kit (Sigma Aldrich) and splice site-specific primers (**Supplementary Table 10**). The data analysis was performed by calculating ΔCq normalized to β-actin expression by the StepOnePlus.

ATRT and breast cancer splicing analysis

Corin-treated breast cancer FASTQ files were downloaded with SRAtoolkit from GEO (Series GSE168644). BAM files were generated using STAR (2.5.2b) (14) against the hg38 reference genome. ATRT FASTQ samples were acquired from collaborators at Johns Hopkins University and aligned to the hg38 reference genome using STAR (2.5.2b) (14). Differential splicing analysis was conducted using rMATS-turbo (v.4.2.0) (20) and AltAnalyze (v.0.7.0.1) (21). Significant events were called using $q < 0.05$ and $|deltaPSI| \geq 0.1$.

TCGA splicing data analysis

OncoSplicing (22) was used to identify survival-associated splicing events ($p < 0.05$) in TCGA-SKCM. Hazard ratios and Kaplan-Meier plots were stored if an event appeared in the list of significant corin-induced splicing events and in the OncoSplicing database. cBioPortal (23) was used to identify the alteration frequency of corin-affected RBP expression across cancers with matched normal tissue data (TCGA pancancer dataset) and to generate the oncoprint.

Peptide synthesis

Candidate neopeptides (10mg) were synthesized by RS Synthesis for ELISpot assays (Louisville, KY, USA) and shown to have > 90% purity by HPLC.

MHC I Immunoprecipitation, peptide purification, and mass spectrometry

Immunoprecipitation with W6/32 MHC Class I antibody (Santa Cruz Biotechnology #SC-32235) and LC-MS/MS analysis were performed following previously established protocols (Klaeger et al., 2021; Sarkizova et al., 2020). HLA peptides were extracted by reconstituting washed beads in 3% acetonitrile/5% formic acid and shaking for 3 minutes. Beads and supernatant were transferred to spin columns (Pierce). Original tubes were washed 2x with 0.1% formic acid and washings added to the spin column. Spin column elutions were applied to the well of a previously activated (80% acetonitrile, 0.1% formic acid) and equilibrated (0.1% formic acid) 40 mg tC18 plate and vacuum applied to pull liquid through. Beads (still on the spin column) were further eluted 3x with 10% acetic acid for 3 minutes with shaking. Each time, after diluting to 1% acetic acid, spin column elutions were applied to the same well of the tC18 plate and vacuum applied. The well was then washed 3x with 0.1% formic acid. Desalted peptides were eluted with 15% acetontrile/1% formic acid, then with 50% acetonitrile, 1% formic acid (into the same tube) and dried by vacuum centrifugation. Peptides were reconstituted in 3% acetonitrile, 0.5% formic acid and applied to EVO tips prepared according to the manufacturer's instructions. EVO tips were eluted twice with 25 µL of 35% acetonitrile/0.1% formic acid, dried by vacuum centrifugation, and stored at -80 °C. For analysis, peptides were reconstituted in 3% acetronitrile/0.5% formic acid, transferred to total recovery vials (Waters, Milford, MA) and analyzed by nanoLC-MS using a NanoElute HPLC system (column=25 cm x 75 µm I.D. packed with 1.9 µm Dr. Maisch C18 with integrated emitted tip (24) interfaced to a timsTOF SCP mass spectrometer (Bruker, Billerica, MA). Peptides were eluted with an HPLC gradient (0-15% B in 60 minutes, 15-23% B in 90 minutes, 23-35% B in 10 minutes, and 35-80% B in 10 minutes; A=0.1% formic acid, B=0.1% formic acid in acetonitrile) at a flow rate of 200 nL/min. Peptides were analyzed by 10 cycles of DDA-PASEF using a 200 ms ramp from 1/k0 0.6-1.6 and *m/z* 200-1700. Active exclusion was enabled with a release time of 0.4 minutes, threshold was 750 counts, and target was 20000 counts. Peptides derived from SE events and identified by SpliceTools were searched using

MSFragger (25) version 20 installed on Bridges2 (Pittsburgh Supercomputing Facility). Additionally, peptides derived from RI, 3'SS, 5'SS, MXE, or trans-splicing events and identified by the SNAF (26) pipeline were searched using SNAF's MaxQuant (27) wrapper function following default parameters. Peptides with FDR < 0.05 from either analysis were considered. Peptides were classified as neopeptides if the peptide sequence did not appear in the UCSC database containing the human proteome appended with virus and reverse decoy sequences.

10x scRNA-seq

About 23,000 cells were loaded onto a 10× Genomics ChromiumTM X instrument (10x Genomics) according to the manufacturer's recommendations. The scRNAseq libraries were processed using Chromium GEM-X Single Cell 5' Kit v3 (10x Genomics). Quality controls for amplified cDNA libraries and final sequencing libraries were performed using Bioanalyzer High Sensitivity DNA Kit (Agilent). The sequencing libraries for scRNAseq were normalized to 4nM concentration and pooled. The pooled sequencing libraries were sequenced on the Illumina NovaSeq S4 300 cycle platform. The sequencing parameters were: Read 1 of 28bp, Read 2 of 90bp, Index 1 of 10bp and Index 2 of 10bp.

Single-cell data processing and cluster annotation

Transcriptomic data were mapped to the Mus musculus reference genome (GRCm39) and assigned to individual cells of origin using Cell Ranger software 8.0 (10X Genomics). Subsequent count matrices were read into Seurat (v5.0.11) (28) for downstream analysis. Doublets were identified and removed for each sample individually using the DoubletFinder (v2.0.4) (29) pipeline. The data were further filtered to exclude cells with high mitochondrial gene expression (>10%) and aberrant unique feature counts (<600, >4000) or contaminating non-immune cells. Samples were then merged together for normalization and identification of highly variable features using

scTransform (v0.4.13)(30). Dimensionality reduction was carried out via principal component analysis using 20 dimensions. The optimal number of clusters was identified using clustree (v0.5.1)(31) and each cluster was annotated based on the top genes determined by FindAllMarkers function in Seurat and SingleR (32) annotations. The T cell population was further subset based on Cd3e expression to determine effects of corin treatment.

Single cell differential gene expression and pathway enrichment

Differential expression analysis was conducted via the FindMarkers function in Seurat, using the statistical framework introduced by MAST (33). To further explore the biological processes and pathways associated with these differentially expressed genes, gene set enrichment analysis was performed using the FGSEA (v1.26.0) (34) package with 50,000 permutations, drawing on gene sets from the Molecular Signatures Database (MSigDB).

Single cell differential cell type proportion analysis

A binomial generalized linear model (GLM) framework was employed using the lmerTest (v3.1.3) (35) package to compare the abundance of different cell types between experimental conditions. Differential cell type proportions were quantified as log odds ratios, indicating enrichment or depletion of cell types across conditions. Estimated marginal means and pairwise contrasts were obtained using the emmeans (v1.10.1) package.

RBP activity prediction

RBP activity prediction was performed using RNA-SPRINT from the SNAF (26) pipeline with default parameters. RBP activity scores and matched RBP gene expression (TPM) for each sample were used to calculate Spearman's statistic using the stats R package (v.3.6.2). U2AF2 motif enrichment coverage plots were generated for significant SKMEL5 SE events using rMAPS2 (36).

Supplementary References

1. Kalin JH, Wu M, Gomez AV, et al. Targeting the CoREST complex with dual histone deacetylase and demethylase inhibitors. *Nat Commun*. 2018;9(1):53. doi:10.1038/s41467-017-02242-4

2. Lee K, Barone M, Waterbury AL, et al. Uncoupling histone modification crosstalk by engineering lysine demethylase LSD1. *Nat Chem Biol*. Published online July 4, 2024. doi:10.1038/s41589-024-01671-9

3. Szewczuk LM, Culhane JC, Yang M, Majumdar A, Yu H, Cole PA. Mechanistic analysis of a suicide inactivator of histone demethylase LSD1. *Biochemistry*. 2007;46(23):6892-6902. doi:10.1021/bi700414b

4. Aguilan JT, Kulej K, Sidoli S. Guide for protein fold change and *p* -value calculation for non-experts in proteomics. *Mol Omics*. 2020;16(6):573-582. doi:10.1039/D0MO00087F

5. Punjani A, Rubinstein JL, Fleet DJ, Brubaker MA. cryoSPARC: algorithms for rapid unsupervised cryo-EM structure determination. *Nat Methods*. 2017;14(3):290-296. doi:10.1038/nmeth.4169

6. Punjani A, Zhang H, Fleet DJ. Non-uniform refinement: adaptive regularization improves single-particle cryo-EM reconstruction. *Nat Methods*. 2020;17(12):1214-1221. doi:10.1038/s41592-020-00990-8

7. Forneris F, Binda C, Adamo A, Battaglioli E, Mattevi A. Structural basis of LSD1-CoREST selectivity in histone H3 recognition. *J Biol Chem*. 2007;282(28):20070-20074. doi:10.1074/jbc.C700100200

8. Glasser E, Agrawal AA, Jenkins JL, Kielkopf CL. Cancer-Associated Mutations Mapped on High-Resolution Structures of the U2AF2 RNA Recognition Motifs. *Biochemistry*. 2017;56(36):4757-4761. doi:10.1021/acs.biochem.7b00551

9. Pettersen EF, Goddard TD, Huang CC, et al. UCSF ChimeraX: Structure visualization for researchers, educators, and developers. *Protein Sci Publ Protein Soc*. 2021;30(1):70-82. doi:10.1002/pro.3943

10. Emsley P, Lohkamp B, Scott WG, Cowtan K. Features and development of Coot. *Acta Crystallogr D Biol Crystallogr*. 2010;66(Pt 4):486-501. doi:10.1107/S0907444910007493

11. Liebschner D, Afonine PV, Baker ML, et al. Macromolecular structure determination using X-rays, neutrons and electrons: recent developments in Phenix. *Acta Crystallogr Sect Struct Biol*. 2019;75(Pt 10):861-877. doi:10.1107/S2059798319011471

12. Williams CJ, Headd JJ, Moriarty NW, et al. MolProbity: More and better reference data for improved all-atom structure validation. *Protein Sci Publ Protein Soc*. 2018;27(1):293-315. doi:10.1002/pro.3330

13. Bolger AM, Lohse M, Usadel B. Trimmomatic: a flexible trimmer for Illumina sequence data. *Bioinformatics*. 2014;30(15):2114-2120. doi:10.1093/bioinformatics/btu170

14. Dobin A, Davis CA, Schlesinger F, et al. STAR: ultrafast universal RNA-seq aligner. *Bioinformatics*. 2013;29(1):15-21. doi:10.1093/bioinformatics/bts635

15. Liao Y, Smyth GK, Shi W. The Subread aligner: fast, accurate and scalable read mapping by seed-and-vote. *Nucleic Acids Res*. 2013;41(10):e108-e108. doi:10.1093/nar/gkt214

16. Love MI, Huber W, Anders S. Moderated estimation of fold change and dispersion for RNA-seq data with DESeq2. *Genome Biol*. 2014;15(12):550. doi:10.1186/s13059-014-0550-8

17. Subramanian A, Tamayo P, Mootha VK, et al. Gene set enrichment analysis: A knowledge-based approach for interpreting genome-wide expression profiles. *Proc Natl Acad Sci*. 2005;102(43):15545-15550. doi:10.1073/pnas.0506580102

18. Kanehisa M, Furumichi M, Tanabe M, Sato Y, Morishima K. KEGG: new perspectives on genomes, pathways, diseases and drugs. *Nucleic Acids Res*. 2017;45(D1):D353-D361. doi:10.1093/nar/gkw1092

19. Liberzon A, Birger C, Thorvaldsdóttir H, Ghandi M, Mesirov JP, Tamayo P. The Molecular Signatures Database Hallmark Gene Set Collection. *Cell Syst*. 2015;1(6):417-425. doi:10.1016/j.cels.2015.12.004

20. Wang Y, Xie Z, Kutschera E, Adams JI, Kadash-Edmondson KE, Xing Y. rMATS-turbo: an efficient and flexible computational tool for alternative splicing analysis of large-scale RNA-seq data. *Nat Protoc*. 2024;19(4):1083-1104. doi:10.1038/s41596-023-00944-2

21. Emig D, Salomonis N, Baumbach J, Lengauer T, Conklin BR, Albrecht M. AltAnalyze and DomainGraph: analyzing and visualizing exon expression data. *Nucleic Acids Res*. 2010;38(Web Server issue):W755-762. doi:10.1093/nar/gkq405

22. Zhang Y, Yao X, Zhou H, et al. OncoSplicing: an updated database for clinically relevant alternative splicing in 33 human cancers. *Nucleic Acids Res*. 2022;50(D1):D1340-D1347. doi:10.1093/nar/gkab851

23. Gao J, Aksoy BA, Dogrusoz U, et al. Integrative analysis of complex cancer genomics and clinical profiles using the cBioPortal. *Sci Signal*. 2013;6(269):pl1. doi:10.1126/scisignal.2004088

24. Ficarro SB, Zhang Y, Lu Y, et al. Improved electrospray ionization efficiency compensates for diminished chromatographic resolution and enables proteomics analysis of tyrosine signaling in embryonic stem cells. *Anal Chem*. 2009;81(9):3440-3447. doi:10.1021/ac802720e

25. Kong AT, Leprevost FV, Avtonomov DM, Mellacheruvu D, Nesvizhskii AI. MSFragger: ultrafast and comprehensive peptide identification in mass spectrometry-based proteomics. *Nat Methods*. 2017;14(5):513-520. doi:10.1038/nmeth.4256

26. Li G, Mahajan S, Ma S, et al. Splicing neoantigen discovery with SNAF reveals shared targets for cancer immunotherapy. *Sci Transl Med*. 2024;16(730):eade2886. doi:10.1126/scitranslmed.ade2886

27. Tyanova S, Temu T, Cox J. The MaxQuant computational platform for mass spectrometry-based shotgun proteomics. *Nat Protoc*. 2016;11(12):2301-2319. doi:10.1038/nprot.2016.136

28. Hao Y, Stuart T, Kowalski MH, et al. Dictionary learning for integrative, multimodal and scalable single-cell analysis. *Nat Biotechnol*. 2024;42(2):293-304. doi:10.1038/s41587-023-01767-y

29. McGinnis CS, Murrow LM, Gartner ZJ. DoubletFinder: Doublet Detection in Single-Cell RNA Sequencing Data Using Artificial Nearest Neighbors. *Cell Syst*. 2019;8(4):329-337.e4. doi:10.1016/j.cels.2019.03.003

30. Hafemeister C, Satija R. Normalization and variance stabilization of single-cell RNA-seq data using regularized negative binomial regression. *Genome Biol*. 2019;20(1):296. doi:10.1186/s13059-019-1874-1

31. Zappia L, Oshlack A. Clustering trees: a visualization for evaluating clusterings at multiple resolutions. *GigaScience*. 2018;7(7):giy083. doi:10.1093/gigascience/giy083

32. Aran D, Looney AP, Liu L, et al. Reference-based analysis of lung single-cell sequencing reveals a transitional profibrotic macrophage. *Nat Immunol*. 2019;20(2):163-172. doi:10.1038/s41590-018-0276-y

33. Finak G, McDavid A, Yajima M, et al. MAST: a flexible statistical framework for assessing transcriptional changes and characterizing heterogeneity in single-cell RNA sequencing data. *Genome Biol*. 2015;16(1):278. doi:10.1186/s13059-015-0844-5

34. Korotkevich G, Sukhov V, Budin N, Shpak B, Artyomov MN, Sergushichev A. Fast gene set enrichment analysis. Preprint posted online February 1, 2021. doi:10.1101/060012

35. Kuznetsova A, Brockhoff PB, Christensen RHB. lmerTest Package: Tests in Linear Mixed Effects Models. *J Stat Softw*. 2017;82(13). doi:10.18637/jss.v082.i13

36. Hwang JY, Jung S, Kook TL, Rouchka EC, Bok J, Park JW. rMAPS2: an update of the RNA map analysis and plotting server for alternative splicing regulation. *Nucleic Acids Res*. 2020;48(W1):W300-W306. doi:10.1093/nar/gkaa237

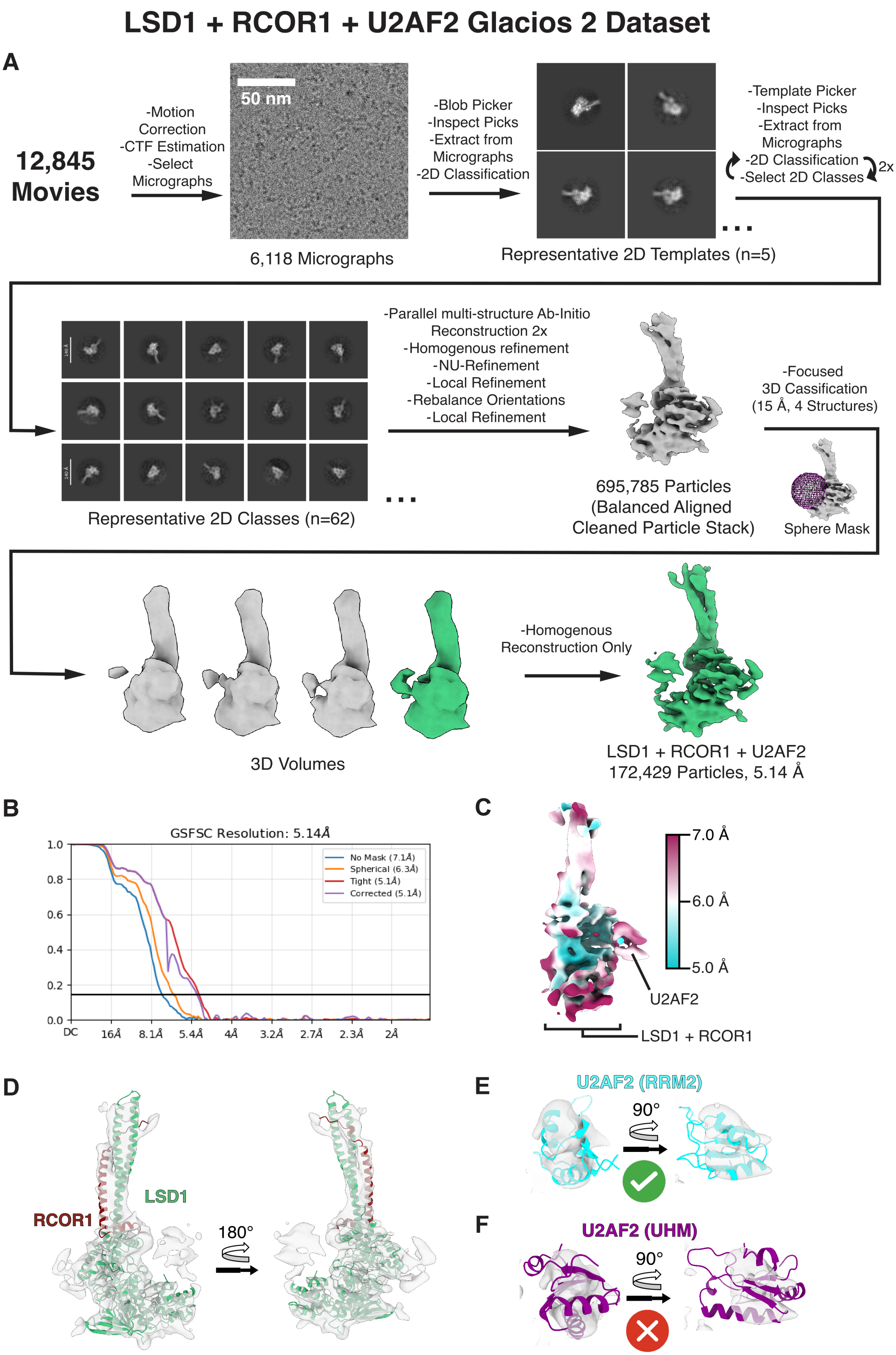

LSD1 + RCOR1 + U2AF2 Glacios 2 Dataset
A
12,845 Movies
-Motion Correction
-CTF Estimation
-Select Micrographs
50 nm
6,118 Micrographs
-Blob Picker
-Inspect Picks
-Extract from Micrographs
-2D Classification
Representative 2D Templates (n=5)
-Template Picker
-Inspect Picks
-Extract from Micrographs
-2D Classification
-Select 2D Classes
2x
Representative 2D Classes (n=62)
-Parallel multi-structure Ab-Initio Reconstruction 2x
-Homogenous refinement
-NU-Refinement
-Local Refinement
-Rebalance Orientations
-Local Refinement
695,785 Particles
(Balanced Aligned Cleaned Particle Stack)
-Focused 3D Cassification
(15 Å, 4 Structures)
Sphere Mask
3D Volumes
-Homogenous Reconstruction Only
LSD1 + RCOR1 + U2AF2
172,429 Particles, 5.14 Å
B
GSFSC Resolution: 5.14Å
No Mask (7.1Å)
Spherical (6.3Å)
Tight (5.1Å)
Corrected (5.1Å)
1.0
0.8
0.6
0.4
0.2
0.0
DC
16Å
8.1Å
5.4Å
4Å
3.2Å
2.7Å
2.3Å
2Å
C
7.0 Å
6.0 Å
5.0 Å
U2AF2
LSD1 + RCOR1
D
RCOR1
LSD1
180°
E
U2AF2 (RRM2)
90°
F
U2AF2 (UHM)
90°

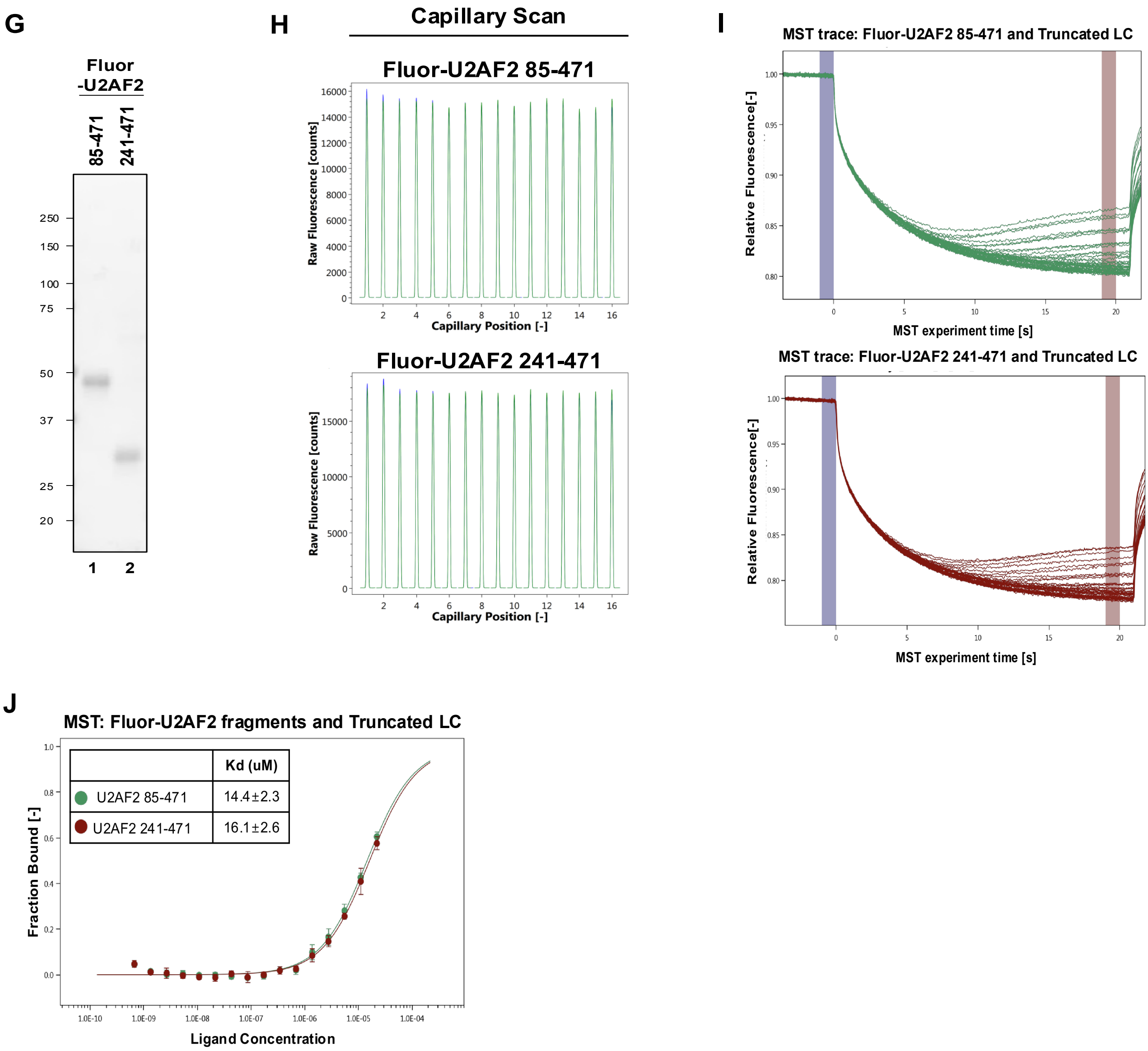
G
Fluor -U2AF2
85-471
241-471
250
150
100
75
50
37
25
20
1
2
H
Capillary Scan
Fluor-U2AF2 85-471
Raw Fluorescence [counts]
Capillary Position [-]
Fluor-U2AF2 241-471
Raw Fluorescence [counts]
Capillary Position [-]
I
MST trace: Fluor-U2AF2 85-471 and Truncated LC
Relative Fluorescence[-]
MST experiment time [s]
MST trace: Fluor-U2AF2 241-471 and Truncated LC
Relative Fluorescence[-]
MST experiment time [s]
J
MST: Fluor-U2AF2 fragments and Truncated LC
Kd (uM)
U2AF2 85-471 14.4±2.3
U2AF2 241-471 16.1±2.6
Fraction Bound [-]
Ligand Concentration

Supplementary Figure 1. Data processing pipeline for cryo-EM analysis and structural superimpositions. (A) Data processing pipeline to determine the cryo-EM structure of U2AF2 bound to the LSD1+RCOR1 complex. **(B)** Fourier Shell correlation plot showing a global resolution of 5.14 Å. **(C)** Local resolution estimation showing a cryo-EM map resolution range from 5.0 Å to 7.0 Å. **(D)** Superimposition of LSD1+RCOR1 over the EM density showing an excellent fit. **(E)** Superimposition of the RRM2 domain of U2AF2 over the U2AF2 EM density showing a good fit. **(F)** Superimposition of the UHM domain of U2AF2 over the U2AF2 EM density showing a poor fit. **(G)** SDS-PAGE and Coomassie staining of purified and fluorophore (Cy5)-labeled U2AF2 fragments. **(H)** Capillary scan data demonstrating successful fluorophore labeling (Right). This data was collected before and after the MST measurement to assess the robustness of fluorophore-labeled U2AF2 fragments binding with truncated LC. Sixteen capillaries were scanned and analyzed, revealing symmetrical peaks with appropriate intensities. **(I)** MST traces measuring the binding affinity between the U2AF2 fragment and truncated LC. A 20-s time window, highlighted in red, has been selected due to its favorable signal-to-noise ratio. The time prior to the IR laser activation is marked in blue. **(J)** Dose-response curve of the MST assay measuring the binding affinity between the U2AF2 fragment and truncated LC. Three replicates were applied.

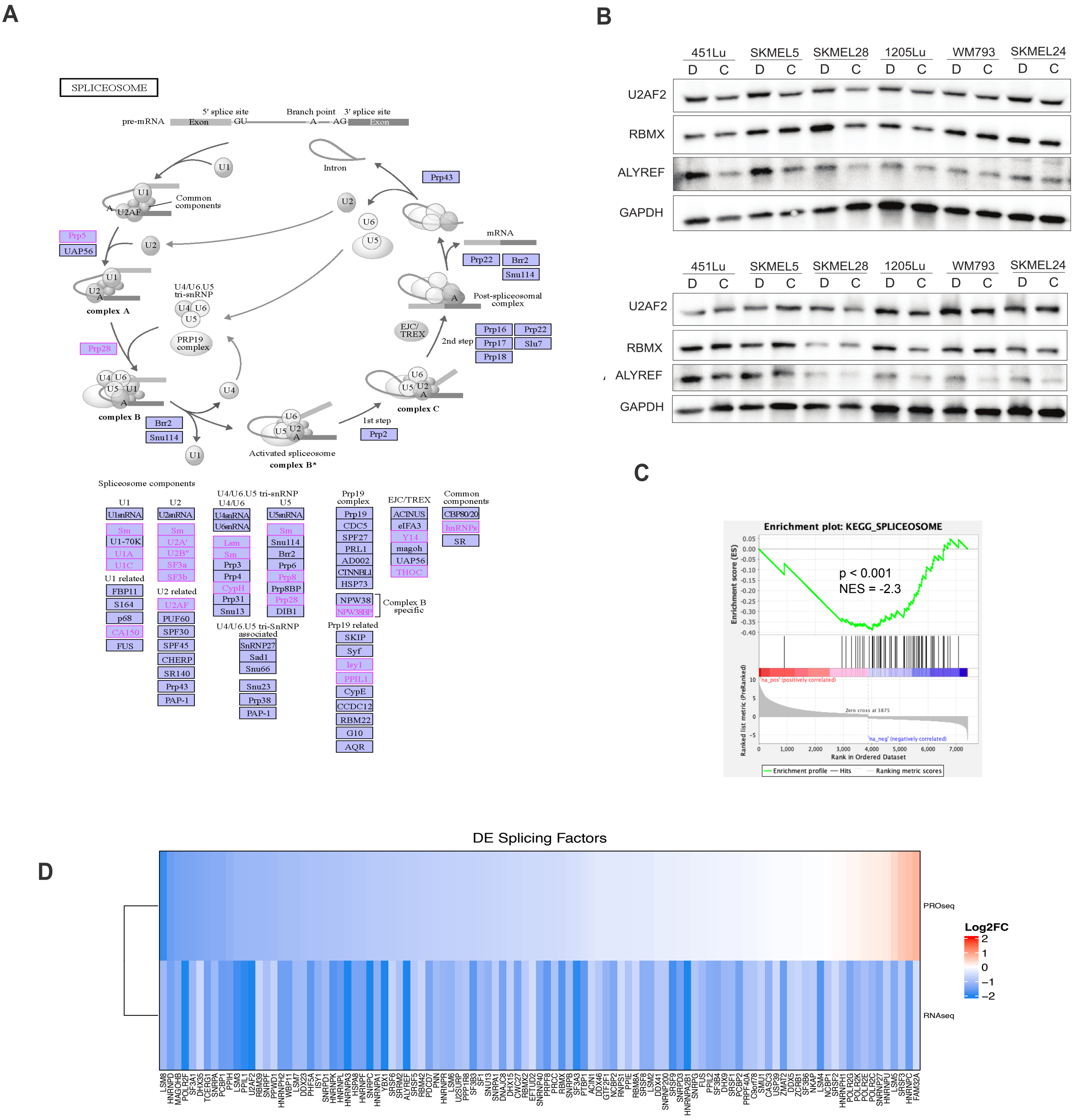
A
SPLICEOSOME
pre-mRNA
5' splice site
Branch point
3' splice site
Exon
GU
A
AG
Intron
U1
U2
U6
U5
U4
Common components
U2AF
Prp5
UAP56
complex A
U4/U6.U5 tri-snRNP
PRP19 complex
Prp28
complex B
Brr2
Snu114
Activated spliceosome
complex B*
Prp2
1st step
complex C
2nd step
Prp16
Prp22
Prp17
Slu7
Prp18
EJC/TREX
Post-spliceosomal complex
mRNA
Prp43
Spliceosome components
U1
U2
U4/U6.U5 tri-snRNP
U4/U6
U5
Prp19 complex
EJC/TREX
Common components
U1snRNA
Sm
U1-70K
U1A
U1C
U1 related
FBP11
S164
p68
CA150
FUS
U2snRNA
U2A'
U2B"
SF3a
SF3b
U2 related
U2AF
PUF60
SPF30
SPF45
CHERP
SR140
Prp43
PAP-1
U4snRNA
U6snRNA
Lsm
Prp3
Prp4
CypH
Prp31
Snu13
U5snRNA
Snu114
Brr2
Prp6
Prp8
Prp8BP
Prp28
DIB1
U4/U6.U5 tri-SnRNP associated
SnRNP27
Sad1
Snu66
Snu23
Prp38
Prp19
CDC5
SPF27
PRL1
AD002
CTNNBL1
HSP73
NPW38
NPW38BP
Complex B specific
Prp19 related
SKIP
Syf
Isy1
PPIL1
CypE
CCDC12
RBM22
G10
AQR
ACINUS
eIFA3
Y14
magoh
THOC
CBP80/20
hnRNPs
SR
B
451Lu
SKMEL5
SKMEL28
1205Lu
WM793
SKMEL24
D
C
U2AF2
RBMX
ALYREF
GAPDH
C
Enrichment plot: KEGG_SPLICEOSOME
p < 0.001
NES = -2.3
Enrichment score (ES)
Ranked list metric (PreRanked)
'na_pos' (positively correlated)
Zero cross at 3875
'na_neg' (negatively correlated)
Rank in Ordered Dataset
Enrichment profile
Hits
Ranking metric scores
D
DE Splicing Factors
PROseq
RNAseq
Log2FC
2
1
0
−1
−2

Supplementary Figure 2. CoREST inhibition does not impact splicing factor transcript steady state. (A) Diagram of KEGG Spliceosome pathway with genes downregulated by corin treatment highlighted in pink. **(B)** Western blot biological replicates used in the quantification of Fig. 3E. **(C)** Gene set enrichment analysis for the KEGG Spliceosome gene set using expression data derived from PRO-seq ($q < 0.05$). **(D)** Heatmap comparing PRO-seq to RNA-seq Log2FC values across the significant downregulated splicing factors.

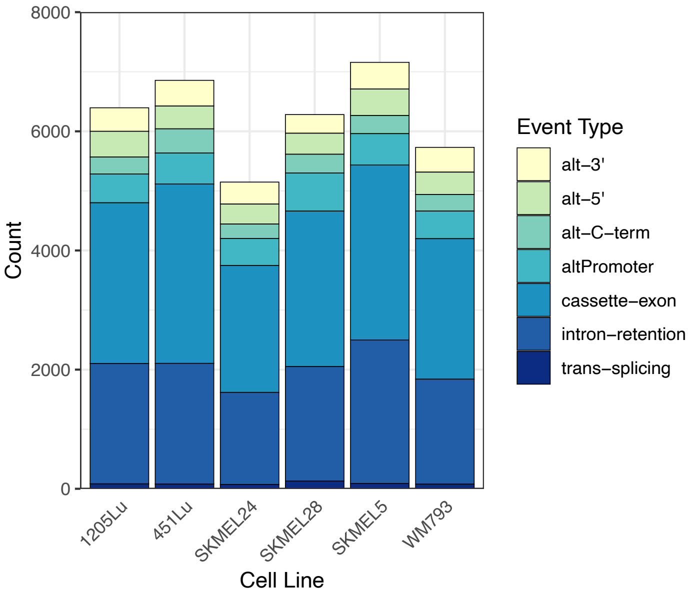

8000
6000
4000
2000
0
Count
1205Lu
451Lu
SKMEL24
SKMEL28
SKMEL5
WM793
Cell Line
Event Type
alt−3'
alt−5'
alt−C−term
altPromoter
cassette−exon
intron−retention
trans−splicing

Supplementary Figure 3. AltAnalyze results of differential RNA splicing in melanoma cell lines. Summary of significant RNA splicing changes across six melanoma cell lines treated with corin (deltaPSI ≥ |0.1|, $p < 0.05$) in duplicate detected by AltAnalyze.

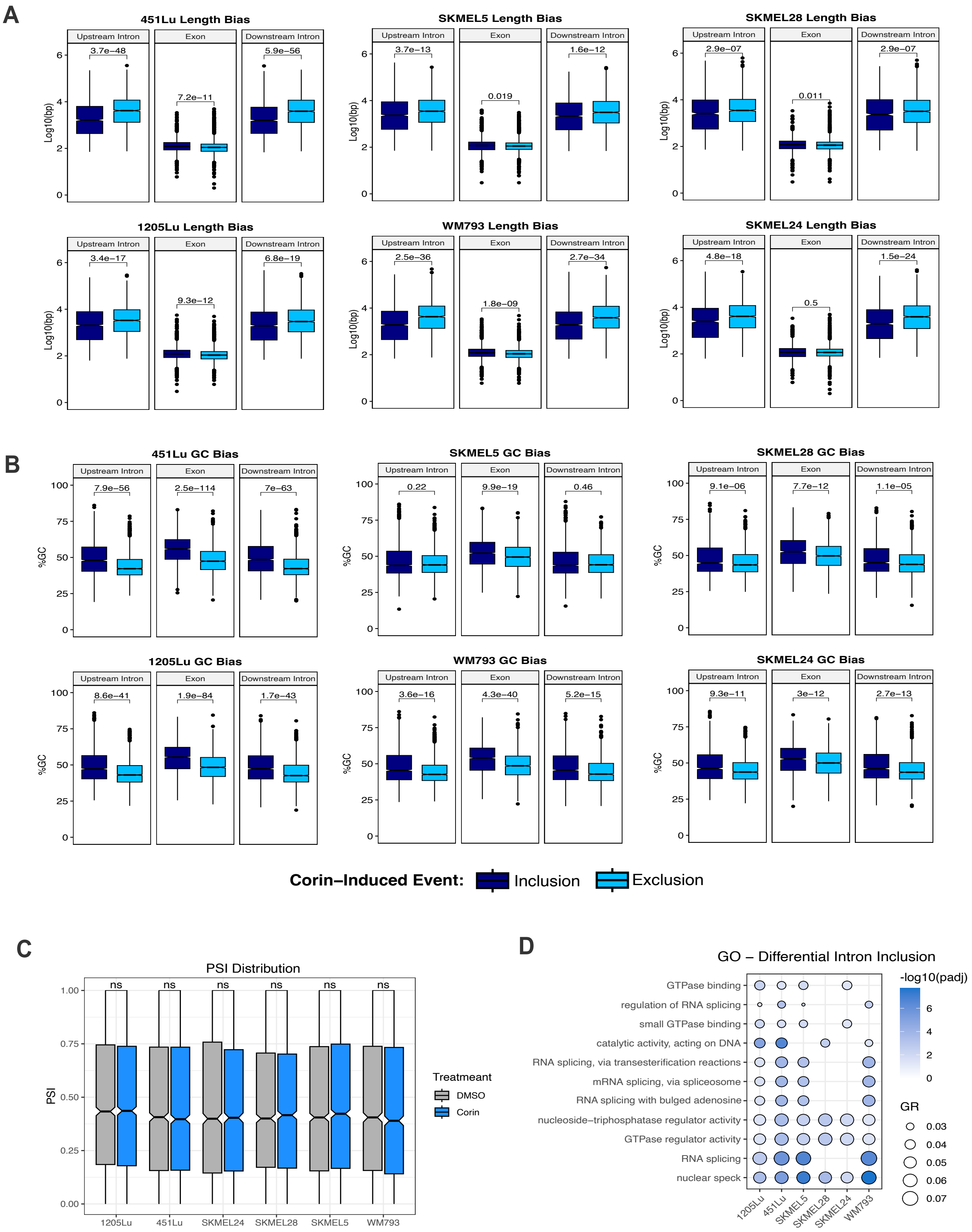
A
451Lu Length Bias
Upstream Intron
Exon
Downstream Intron
3.7e−48
7.2e−11
5.9e−56
Log10(bp)
SKMEL5 Length Bias
3.7e−13
0.019
1.6e−12
SKMEL28 Length Bias
2.9e−07
0.011
2.9e−07
1205Lu Length Bias
3.4e−17
9.3e−12
6.8e−19
WM793 Length Bias
2.5e−36
1.8e−09
2.7e−34
SKMEL24 Length Bias
4.8e−18
0.5
1.5e−24
B
451Lu GC Bias
7.9e−56
2.5e−114
7e−63
%GC
SKMEL5 GC Bias
0.22
9.9e−19
0.46
SKMEL28 GC Bias
9.1e−06
7.7e−12
1.1e−05
1205Lu GC Bias
8.6e−41
1.9e−84
1.7e−43
WM793 GC Bias
3.6e−16
4.3e−40
5.2e−15
SKMEL24 GC Bias
9.3e−11
3e−12
2.7e−13
Corin−Induced Event:
Inclusion
Exclusion
C
PSI Distribution
ns
PSI
1205Lu
451Lu
SKMEL24
SKMEL28
SKMEL5
WM793
Treatmeant
DMSO
Corin
D
GO − Differential Intron Inclusion
GTPase binding
regulation of RNA splicing
small GTPase binding
catalytic activity, acting on DNA
RNA splicing, via transesterification reactions
mRNA splicing, via spliceosome
RNA splicing with bulged adenosine
nucleoside−triphosphatase regulator activity
GTPase regulator activity
RNA splicing
nuclear speck
-log10(padj)
GR
0.03
0.04
0.05
0.06
0.07
1205Lu
451Lu
SKMEL5
SKMEL28
SKMEL24
WM793

Supplementary Figure 4. CoREST inhibition impacts RNA splicing factor intron retention and selectively skips short exons. (A) Exon and proximal intron length bias between differentially included and excluded exons. Statistical analysis was performed using ANOVA to compare lengths across treatment groups, grouped by type of splicing event. P-values were adjusted for multiple comparisons using the Holm-Sidak's correction. **(B)** Exon and proximal GC bias between differentially included and excluded exons. Statistical analysis was performed using ANOVA to compare GC content across treatment groups, grouped by type of splicing event. P-values were adjusted for multiple comparisons using the Holm-Sidak's correction. **(C)** Percent Spliced In (PSI) distribution across 6 melanoma cells lines for significant Intron Retention (RI) events detected by AltAnalyze (deltaPSI $\geq |0.1|$, $p < 0.05$). Statistical comparisons were performed using a two-sample t-test to assess differences in PSI value between treatment groups within each cell line. **(D)** Pathway analysis for significant RI events across the 6 melanoma cell lines ($p < 0.05$). Enrichment analysis was performed using the hypergeometric test with multiple test correction by the Benjamini-Hochberg method.

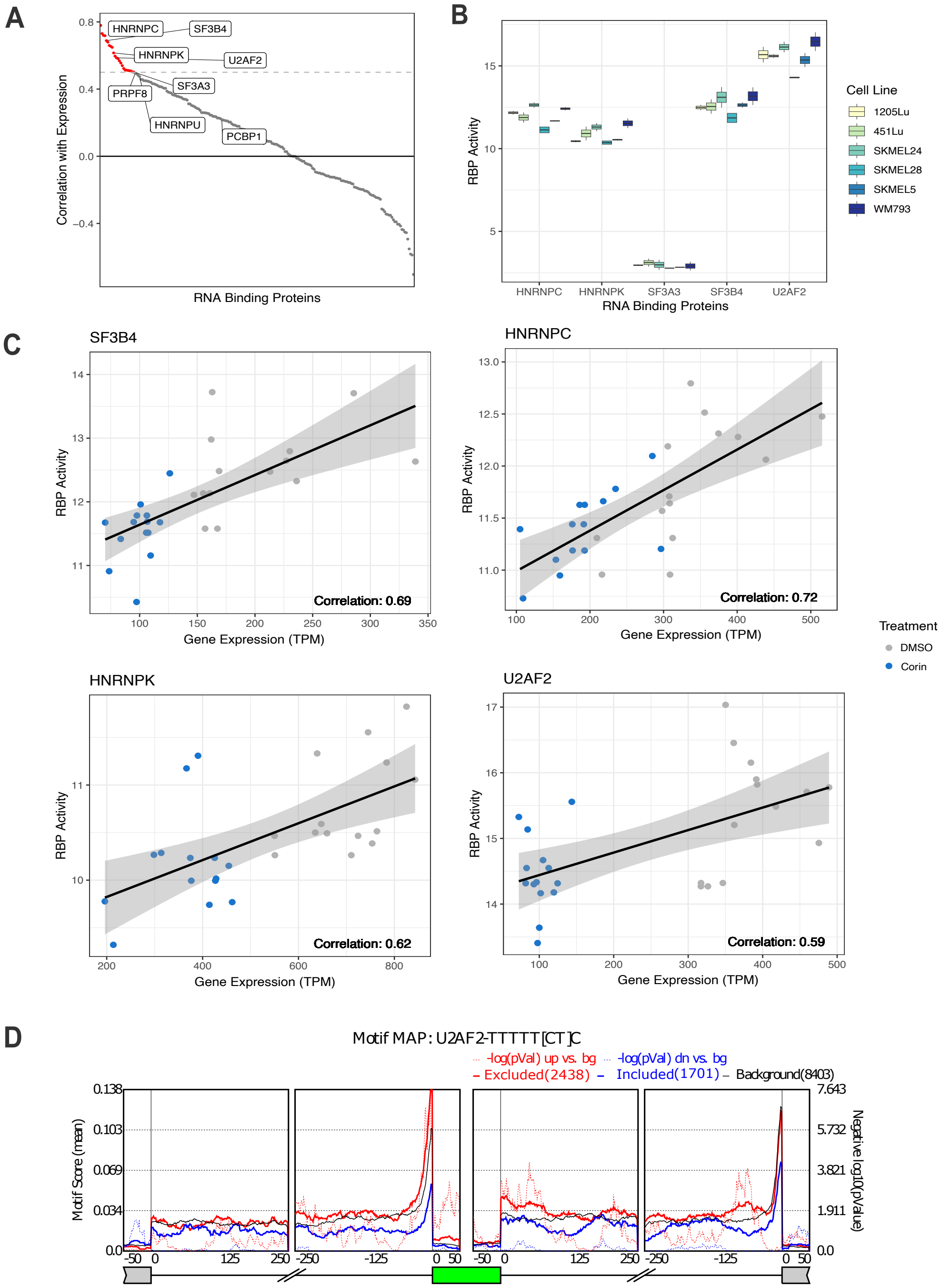
A
Correlation with Expression
RNA Binding Proteins
HNRNPC
SF3B4
HNRNPK
U2AF2
SF3A3
PRPF8
HNRNPU
PCBP1
B
RBP Activity
RNA Binding Proteins
Cell Line
1205Lu
451Lu
SKMEL24
SKMEL28
SKMEL5
WM793
C
SF3B4
HNRNPC
HNRNPK
U2AF2
Correlation: 0.69
Correlation: 0.72
Correlation: 0.62
Correlation: 0.59
Gene Expression (TPM)
Treatment
DMSO
Corin
D
Motif MAP: U2AF2-TTTTT[CT]C
-log(pVal) up vs. bg
-log(pVal) dn vs. bg
Excluded(2438)
Included(1701)
Background(8403)
Motif Score (mean)
Negative log10(pValue)

Supplementary Figure 5. RNA-SPRINT analysis of RBP activity correlated with corin-induced expression changes. (A) Rank plot of Spearman's rank correlation between RBP activity and expression with corin treatment. Red points denote RBPs with a correlation value > 0.5. **(B)** RBP activity scores for each candidate RBP determined in (A) across all 6 melanoma cell lines. **(C)** Individual correlation plots for each candidate RBP. **(D)** rMAPS2 coverage plots for U2AF2 binding scores in significantly included (blue) and excluded (red) exons in SKMEL5 cells treated with corin compared to a randomized background list of exons (black). Motif scores are represented by solid lines and p-values are represented by dashed lines. The window is centered at the expons of interest (green box).

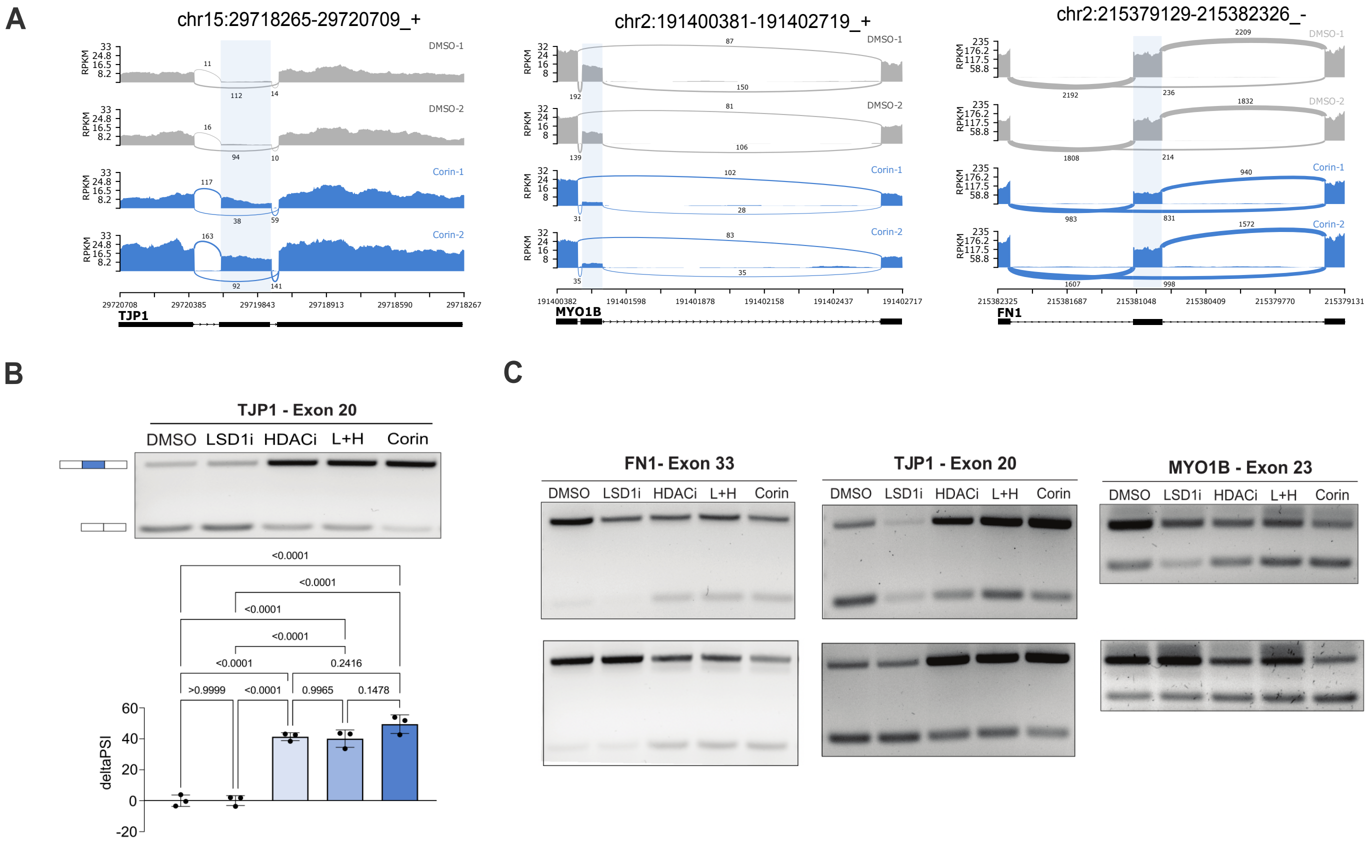
A
chr15:29718265-29720709_+
chr2:191400381-191402719_+
chr2:215379129-215382326_-
DMSO-1
DMSO-2
Corin-1
Corin-2
RPKM
TJP1
MYO1B
FN1
B
TJP1 - Exon 20
DMSO
LSD1i
HDACi
L+H
Corin
deltaPSI
<0.0001
0.2416
>0.9999
0.9965
0.1478
C
FN1- Exon 33
TJP1 - Exon 20
MYO1B - Exon 23

Supplementary Figure 6. Validation of corin-induced RNA splicing changes. (A) Sashimi plots of MYO1B, TJP1, and FN1 splicing based on RNA-seq data. **(B)** TJP1 exon 20 RT-PCR gel comparing DMSO, LSD1i, HDACi, LSD1i + HDACi, and corin's impact on exon inclusion. Statistical analysis of biological replicates (n=3) was performed using an unpaired, two-tailed t-test. Error bars represent the standard deviation (SD). **(C)** RT-PCR gel biological replicates used for quantification in Fig. 5A and S5B.

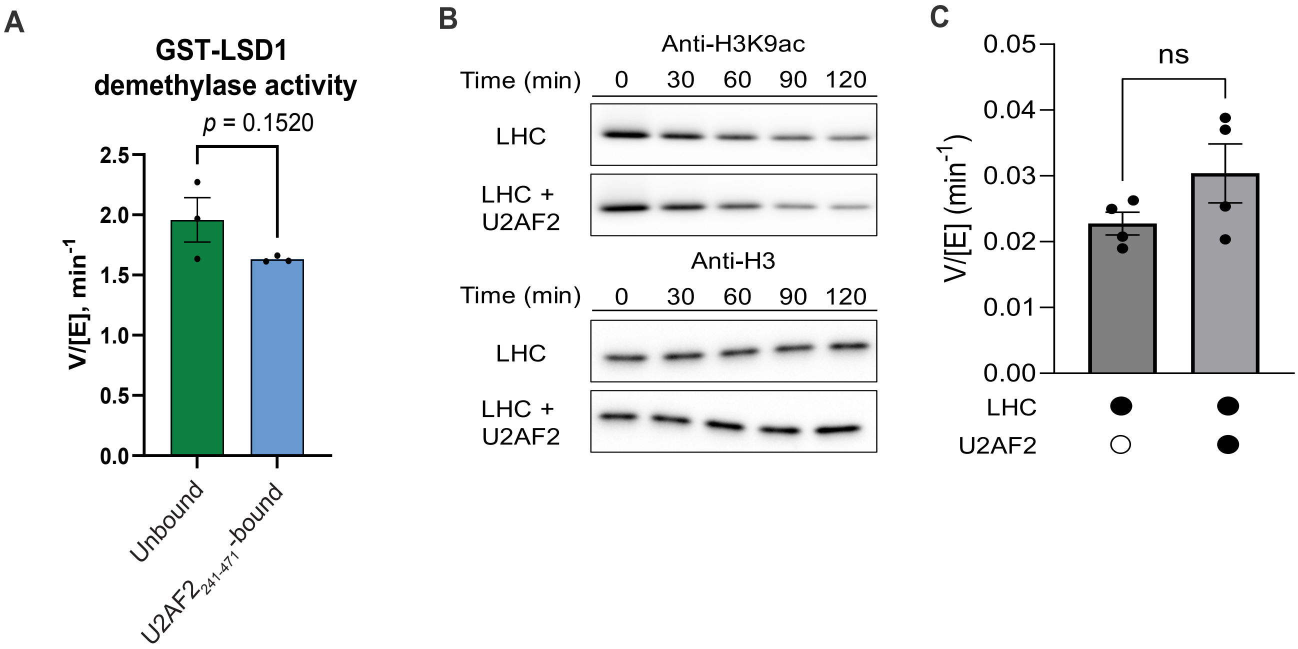
A
GST-LSD1
demethylase activity
p = 0.1520
2.5
2.0
1.5
1.0
0.5
0.0
V/[E], min-1
Unbound
U2AF2241-471-bound
B
Anti-H3K9ac
Time (min)
0
30
60
90
120
LHC
LHC +
U2AF2
Anti-H3
Time (min)
0
30
60
90
120
LHC
LHC +
U2AF2
C
0.05
0.04
0.03
0.02
0.01
0.00
ns
V/[E] (min-1)
LHC
U2AF2

Supplementary Figure 7. U2AF2-binding does not impact LSD1's demethylase or LHC's deacetylase activity. (A) The effect of U2AF2 on LSD1 demethylase activity was evaluated using a horse radish peroxidase (HRP)-coupled peptide demethylase assay. GST-LSD1$_{171\text{-}852}$ and H3K4me2 peptide (aa 1-21) were used as the enzyme and the substrate, respectively, in the assay with and without U2AF2$_{241\text{-}471}$. H_2O_2 generated from the demethylation reaction was coupled with HRP to catalyze a chromogenic reaction between 4-aminoantipyrine and 3, 5-dichloro-2-hydroxybenzenesulfonate (DHBS) that allows detection at 515 nm Initial rates of product formation (V/[E], min^{-1}) were calculated from three technical replicates, based on measurements taken between 2–4 minutes of the reaction. Data were analyzed using Graphpad Prism 10 with an unpaired, two-tailed Student's t-test, testing the null hypothesis that LSD1 activity does not differ with and without U2AF2$_{241\text{-}471}$. **(B)** Representative western-blot images describing deacetylation of H3K9ac nucleosome by the LHC complex with or without U2AF2. **(C)** Enzymatic activity (V/[E]) of the LHC complex toward the H3K9ac site on nucleosomes in the absence and presence of U2AF2. Conditions: [Nucleosome] = 100 nM; [LHC] = 40 nM; [U2AF2] = 10 μM; All values denote mean ± s.e.m; n = 4; Student's *t*-test.

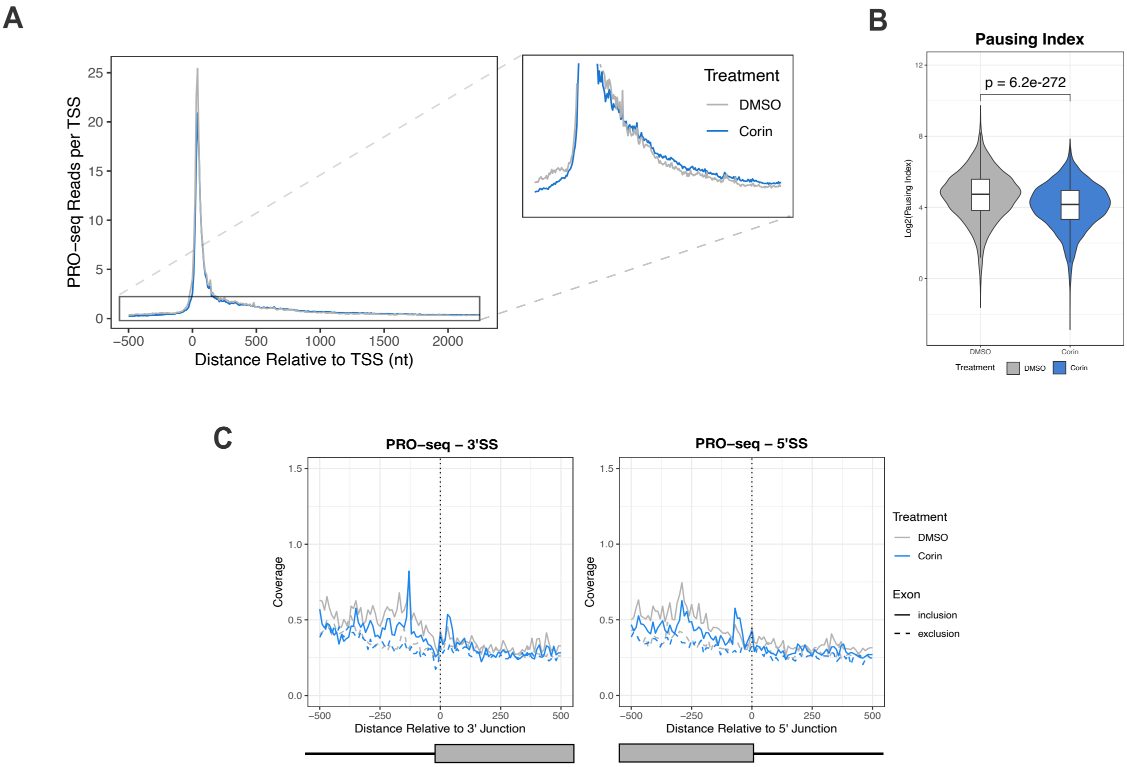
A
PRO−seq Reads per TSS
25
20
15
10
5
0
−500
0
500
1000
1500
2000
Distance Relative to TSS (nt)
Treatment
DMSO
Corin
B
Pausing Index
p = 6.2e-272
Log2(Pausing Index)
12
8
4
0
DMSO
Corin
Treatment
DMSO
Corin
C
PRO−seq − 3'SS
PRO−seq − 5'SS
Coverage
1.5
1.0
0.5
0.0
−500
−250
0
250
500
Distance Relative to 3' Junction
Distance Relative to 5' Junction
Treatment
DMSO
Corin
Exon
inclusion
exclusion

Supplementary Figure 8. Corin promotes promoter pause release without impacting transcriptional kinetics at splice sites. **(A)** PRO-seq coverage metagene plot centered at TSS's under DMSO or corin conditions. **(B)** Pausing index calculated between treatments. Statisical analysis performed using a two-sided two-sample t-test. **(C)** PRO-seq coverage metagene plots at 3'SS and 5'SS for significantly included and excluded skipped exon events (deltaPSI $\geq |0.1|$, $q < 0.05$).

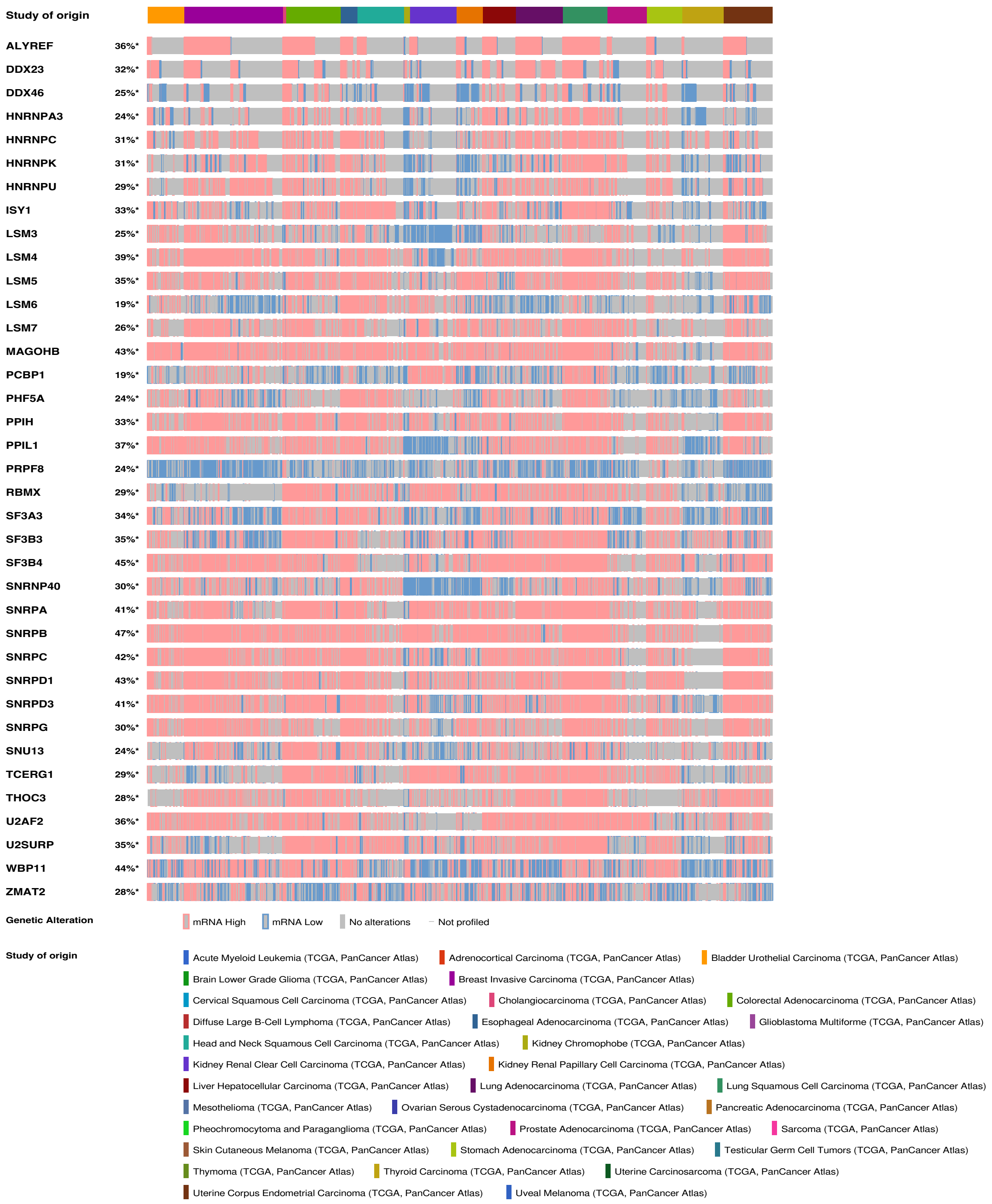
Study of origin
ALYREF 36%*
DDX23 32%*
DDX46 25%*
HNRNPA3 24%*
HNRNPC 31%*
HNRNPK 31%*
HNRNPU 29%*
ISY1 33%*
LSM3 25%*
LSM4 39%*
LSM5 35%*
LSM6 19%*
LSM7 26%*
MAGOHB 43%*
PCBP1 19%*
PHF5A 24%*
PPIH 33%*
PPIL1 37%*
PRPF8 24%*
RBMX 29%*
SF3A3 34%*
SF3B3 35%*
SF3B4 45%*
SNRNP40 30%*
SNRPA 41%*
SNRPB 47%*
SNRPC 42%*
SNRPD1 43%*
SNRPD3 41%*
SNRPG 30%*
SNU13 24%*
TCERG1 29%*
THOC3 28%*
U2AF2 36%*
U2SURP 35%*
WBP11 44%*
ZMAT2 28%*
Genetic Alteration
mRNA High
mRNA Low
No alterations
Not profiled
Study of origin
Acute Myeloid Leukemia (TCGA, PanCancer Atlas)
Adrenocortical Carcinoma (TCGA, PanCancer Atlas)
Bladder Urothelial Carcinoma (TCGA, PanCancer Atlas)
Brain Lower Grade Glioma (TCGA, PanCancer Atlas)
Breast Invasive Carcinoma (TCGA, PanCancer Atlas)
Cervical Squamous Cell Carcinoma (TCGA, PanCancer Atlas)
Cholangiocarcinoma (TCGA, PanCancer Atlas)
Colorectal Adenocarcinoma (TCGA, PanCancer Atlas)
Diffuse Large B-Cell Lymphoma (TCGA, PanCancer Atlas)
Esophageal Adenocarcinoma (TCGA, PanCancer Atlas)
Glioblastoma Multiforme (TCGA, PanCancer Atlas)
Head and Neck Squamous Cell Carcinoma (TCGA, PanCancer Atlas)
Kidney Chromophobe (TCGA, PanCancer Atlas)
Kidney Renal Clear Cell Carcinoma (TCGA, PanCancer Atlas)
Kidney Renal Papillary Cell Carcinoma (TCGA, PanCancer Atlas)
Liver Hepatocellular Carcinoma (TCGA, PanCancer Atlas)
Lung Adenocarcinoma (TCGA, PanCancer Atlas)
Lung Squamous Cell Carcinoma (TCGA, PanCancer Atlas)
Mesothelioma (TCGA, PanCancer Atlas)
Ovarian Serous Cystadenocarcinoma (TCGA, PanCancer Atlas)
Pancreatic Adenocarcinoma (TCGA, PanCancer Atlas)
Pheochromocytoma and Paraganglioma (TCGA, PanCancer Atlas)
Prostate Adenocarcinoma (TCGA, PanCancer Atlas)
Sarcoma (TCGA, PanCancer Atlas)
Skin Cutaneous Melanoma (TCGA, PanCancer Atlas)
Stomach Adenocarcinoma (TCGA, PanCancer Atlas)
Testicular Germ Cell Tumors (TCGA, PanCancer Atlas)
Thymoma (TCGA, PanCancer Atlas)
Thyroid Carcinoma (TCGA, PanCancer Atlas)
Uterine Carcinosarcoma (TCGA, PanCancer Atlas)
Uterine Corpus Endometrial Carcinoma (TCGA, PanCancer Atlas)
Uveal Melanoma (TCGA, PanCancer Atlas)

Supplementary Figure 9. CoREST-regulated RNA splicing factors are frequently overexpressed across cancers. Oncoprint plot depicting the frequency of altered mRNA expression alteration in CoREST complex-regulated RNA splicing factors between cancer and matched normal tissue from cBioPortal stratified by cancer type. Pink bars indicate the frequency of overexpression events and blue bars indicate the frequency of downregulation events.

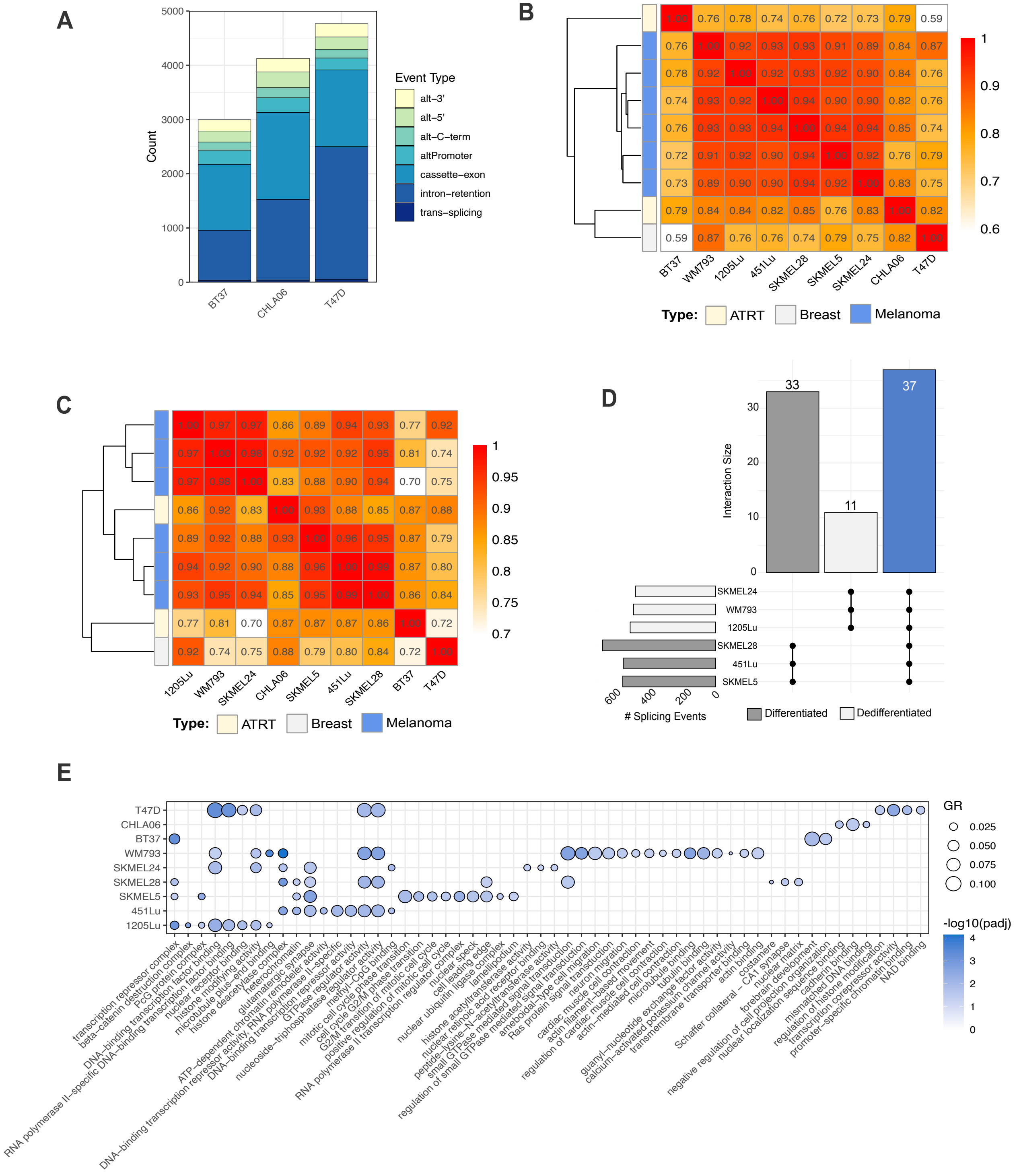
A
Count
Event Type
alt−3'
alt−5'
alt−C−term
altPromoter
cassette−exon
intron−retention
trans−splicing
BT37
CHLA06
T47D
B
1.00 0.76 0.78 0.74 0.76 0.72 0.73 0.79 0.59
0.76 1.00 0.92 0.93 0.93 0.91 0.89 0.84 0.87
0.78 0.92 1.00 0.92 0.93 0.92 0.90 0.84 0.76
0.74 0.93 0.92 1.00 0.94 0.90 0.90 0.82 0.76
0.76 0.93 0.93 0.94 1.00 0.94 0.94 0.85 0.74
0.72 0.91 0.92 0.90 0.94 1.00 0.92 0.76 0.79
0.73 0.89 0.90 0.90 0.94 0.92 1.00 0.83 0.75
0.79 0.84 0.84 0.82 0.85 0.76 0.83 1.00 0.82
0.59 0.87 0.76 0.76 0.74 0.79 0.75 0.82 1.00
BT37 WM793 1205Lu 451Lu SKMEL28 SKMEL5 SKMEL24 CHLA06 T47D
Type: ATRT Breast Melanoma
C
1.00 0.97 0.97 0.86 0.89 0.94 0.93 0.77 0.92
0.97 1.00 0.98 0.92 0.92 0.92 0.95 0.81 0.74
0.97 0.98 1.00 0.83 0.88 0.90 0.94 0.70 0.75
0.86 0.92 0.83 1.00 0.93 0.88 0.85 0.87 0.88
0.89 0.92 0.88 0.93 1.00 0.96 0.95 0.87 0.79
0.94 0.92 0.90 0.88 0.96 1.00 0.99 0.87 0.80
0.93 0.95 0.94 0.85 0.95 0.99 1.00 0.86 0.84
0.77 0.81 0.70 0.87 0.87 0.87 0.86 1.00 0.72
0.92 0.74 0.75 0.88 0.79 0.80 0.84 0.72 1.00
1205Lu WM793 SKMEL24 CHLA06 SKMEL5 451Lu SKMEL28 BT37 T47D
Type: ATRT Breast Melanoma
D
Interaction Size
33
11
37
SKMEL24
WM793
1205Lu
SKMEL28
451Lu
SKMEL5
Splicing Events
Differentiated
Dedifferentiated
E
T47D
CHLA06
BT37
WM793
SKMEL24
SKMEL28
SKMEL5
451Lu
1205Lu
GR
-log10(padj)
transcription repressor complex
beta−catenin destruction complex
PcG protein complex
DNA−binding transcription factor binding
RNA polymerase II−specific DNA−binding transcription factor binding
nuclear receptor binding
histone modifying activity
microtubule plus−end binding
histone deacetylase complex
heterochromatin
glutamatergic synapse
ATP−dependent chromatin remodeler activity
DNA−binding transcription repressor activity, RNA polymerase II−specific
DNA−binding transcription repressor activity
GTPase regulator activity
nucleoside−triphosphatase regulator activity
methyl−CpG binding
mitotic cell cycle phase transition
cell cycle G2/M phase transition
G2/M transition of mitotic cell cycle
positive regulation of mitotic cell cycle
RNA polymerase II transcription regulator complex
nuclear speck
cell leading edge
nuclear ubiquitin ligase complex
lamellipodium
histone acetyltransferase activity
nuclear retinoic acid receptor binding
peptide−lysine−N−acetyltransferase activity
small GTPase mediated signal transduction
regulation of small GTPase mediated signal transduction
ameboidal−type cell migration
Ras protein signal transduction
neuron migration
cardiac muscle cell contraction
actin filament−based movement
regulation of cardiac muscle cell contraction
actin−mediated cell contraction
microtubule binding
tubulin binding
guanyl−nucleotide exchange factor activity
calcium−activated potassium channel activity
transmembrane transporter binding
actin binding
costamere
Schaffer collateral − CA1 synapse
nuclear matrix
forebrain development
negative regulation of cell projection organization
nuclear localization sequence binding
cadherin binding
mismatched DNA binding
regulation of histone modification
transcription corepressor activity
promoter−specific chromatin binding
NAD binding

Supplementary Figure 10. CoREST-regulated splicing is concordant across cancers. (A) Summary of significant RNA splicing changes across ATRT and breast cancer cell lines treated with corin (deltaPSI ≥ |0.1|, $p < 0.05$) in duplicate detected by AltAnalyze. **(B)** Heatmap showing concordance between cell lines based on common SE splicing events. **(C)** Heatmap showing concordance between cell lines based on common AP splicing events. **(D)** UpSert plot comparing the total number of phenotype specific AP events across melanoma cell lines **(E)** GO dotplot of significant pathways impacted by differential AP inclusion across melanoma cell lines.

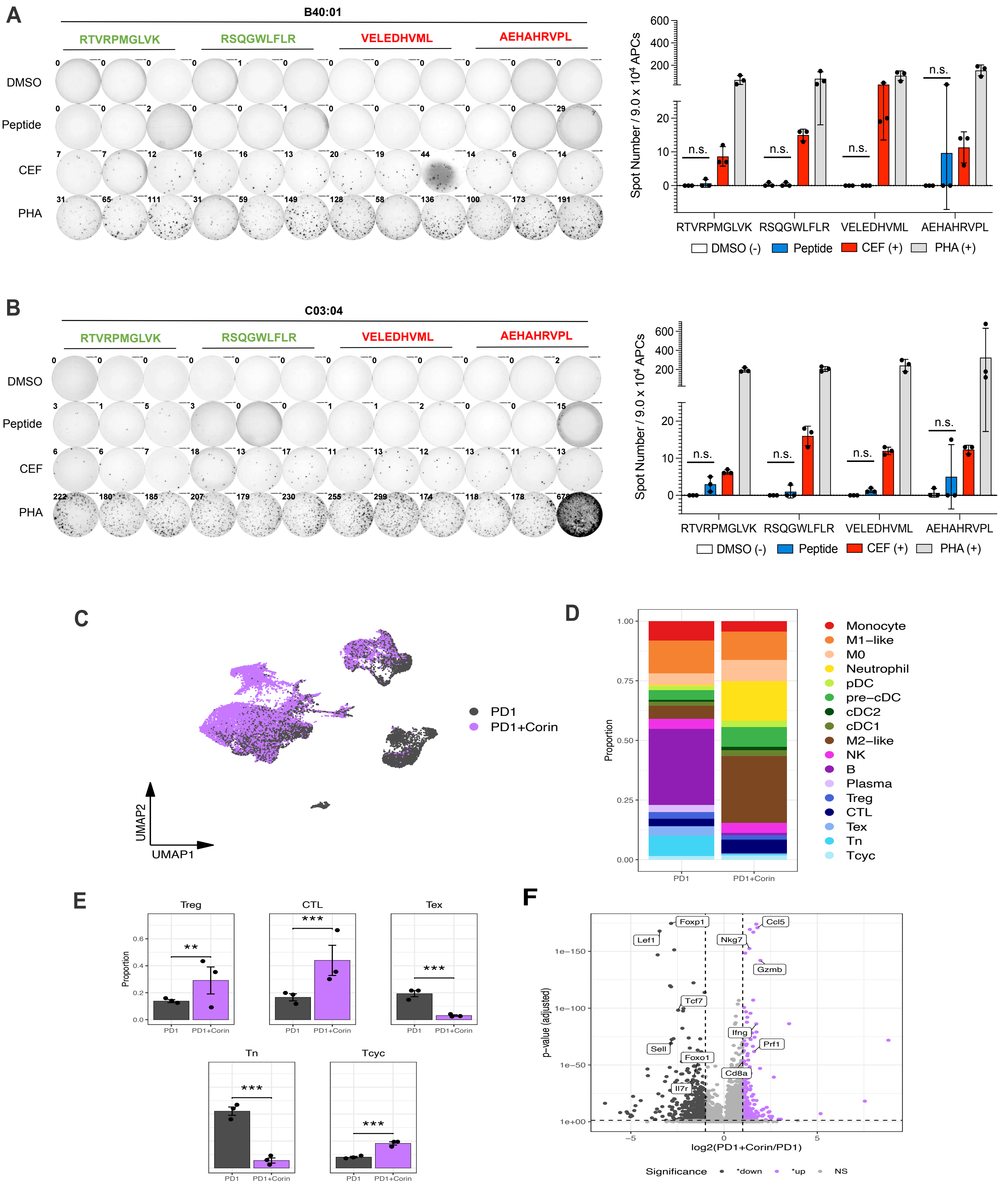
A
B40:01
RTVRPMGLVK
RSQGWLFLR
VELEDHVML
AEHAHRVPL
DMSO
Peptide
CEF
PHA
Spot Number / 9.0 x 10^4 APCs
n.s.
DMSO (-)
Peptide
CEF (+)
PHA (+)
B
C03:04
C
PD1
PD1+Corin
UMAP1
UMAP2
D
Proportion
Monocyte
M1−like
M0
Neutrophil
pDC
pre−cDC
cDC2
cDC1
M2−like
NK
B
Plasma
Treg
CTL
Tex
Tn
Tcyc
E
Treg
CTL
Tex
Tn
Tcyc
F
Foxp1
Lef1
Ccl5
Nkg7
Gzmb
Tcf7
Ifng
Prf1
Sell
Foxo1
Cd8a
Il7r
p−value (adjusted)
log2(PD1+Corin/PD1)
Significance
*down
*up
NS

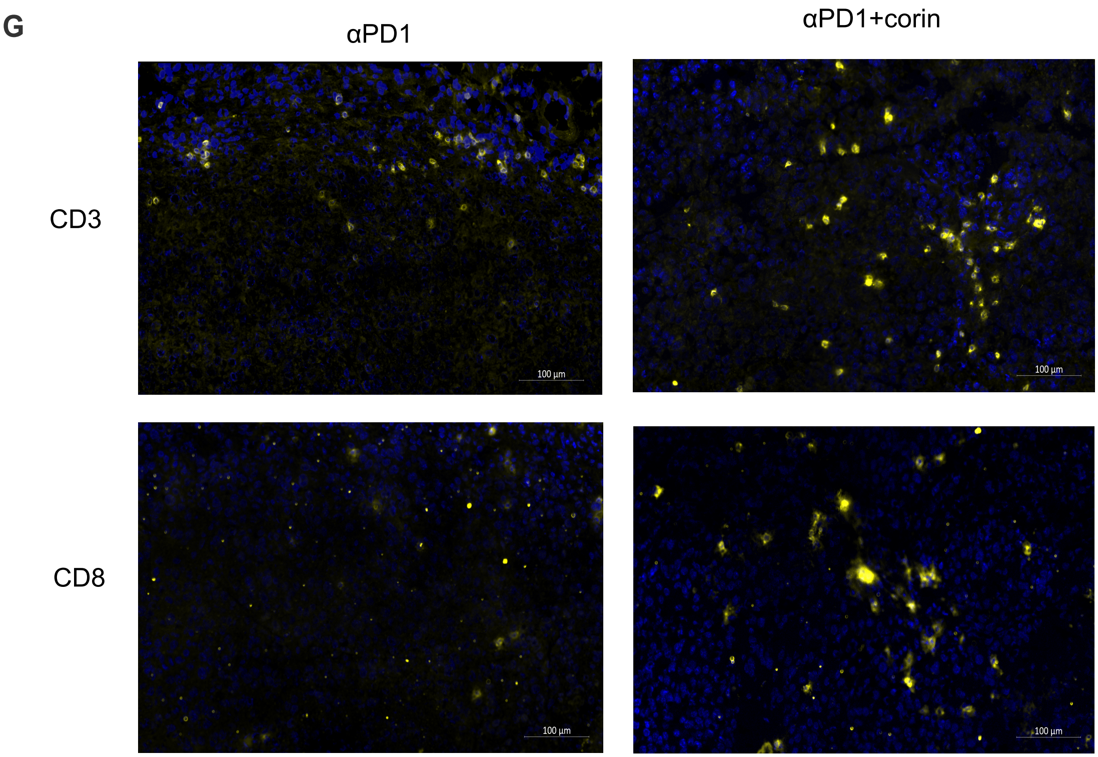
G
αPD1
αPD1+corin
CD3
CD8
100 µm
100 µm
100 µm
100 µm

Supplementary Figure 11. Additional immunological analyses to validate neoantigen immunogenicity and corin's impact on the tumor microenvironment. ELISpot images and quantification for HLA B40:01 **(A)** and HLA C03:04 **(B)**. Statistical analysis was performed using multiple two-tailed unpaired t-tests. Error bars represent the standard deviation (SD). No peptides reached statistical significance. **(C)** scRNA-seq UMAP comparing the immune populations between anti-PD1 and anti-PD1+corin treatment. **(D)** Proportion analysis comparing changes in the immune populations between treatments. **(E)** Barplots and statistical analysis comparing changes in T cell proportions (*p.adj < 0.05, **p.adj < 0.01, ***p.adj < 0.001). **(G)** Volcano plot of differential gene expression in the T cell subset. (G) Representative immunofluorescent images of anti-PD1 and anti-PD1+corin treated B16-F10 melanomas stained for CD3+ and CD8+ T cells.

Supplementary Table 1. Results from the IP-MS using the LSD1 and RCOR1 pulldowns comparing IgG to DMSO. Log2(Fold change) and -Log2(p) values were calculated by comparing signal to IgG control.

Supplementary Table 2. Results from the IP-MS using the LSD1 and RCOR1 pulldowns comparing corin to DMSO. Log2(Fold change) and -Log2(p) values calculated by comparing DMSO signal to corin signal (24h, 2.5uM).

Supplementary Table 3. Significant differential splicing events for melanoma cell lines. Differential events are defined by $q < 0.05$, deltaPSI ≥ |0.1|.

Supplementary Table 4. Pausing Index values calculated from PRO-seq data. PI values from DMSO and corin treated samples.

Supplementary Table 5. CoREST-regulated splicing factors. List of splicing factors significantly downregulated in all melanoma, ATRT, and breast cancer cell lines.

Supplementary Table 6. Significant differential splicing events for ATRT and breast cancer cell lines. Differential events are defined by $q < 0.05$, deltaPSI ≥ |0.1|.

Supplementary Table 7. Summary of significant predicted neopeptides. Neopeptide candidates were selected based on the product of the -$Log10$(%Rank), junction count, and |deltaPSI| value ($q < 0.05$, deltaPSI ≥ |0.1|, $Log2$(TPM) ≥ 3, IJC + SJC ≥ 20, % Rank $\leq$ 2).

Supplementary Table 8. Peptides identified from MHCI IP-MS. Peptides were identified using MSFlagger and MaxQuant (FDR < 0.05).

Supplementary Table 9. List of antibodies.

Supplementary Table 10. List of primers.
