# Supplementary material for: CoREST complex inhibition alters RNA splicing to promote neoantigen expression and enhance tumor immunity
Source: JCI Insight. 2025 Dec 9;11(2):e190287. doi: 10.1172/jci.insight.190287 (PMC12892918; doi:10.1172/jci.insight.190287)

Raw Western Blots & Gels

Figure 1E

Western blots – SKMEL5 treated with DMSO or corin (24h, 2.5uM). Probed for LSD1, RCOR1, U2AF2, SRSF1, GAPDH

Replicate 1: LSD1

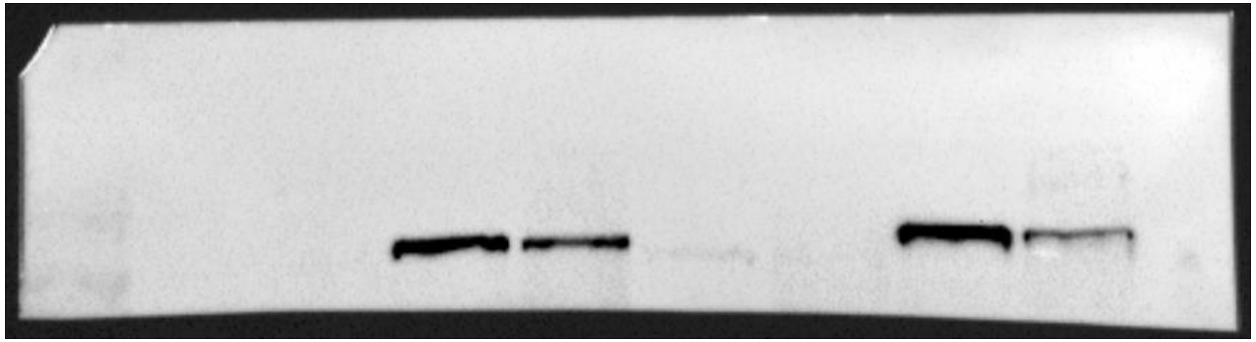

Replicate 1: RCOR1

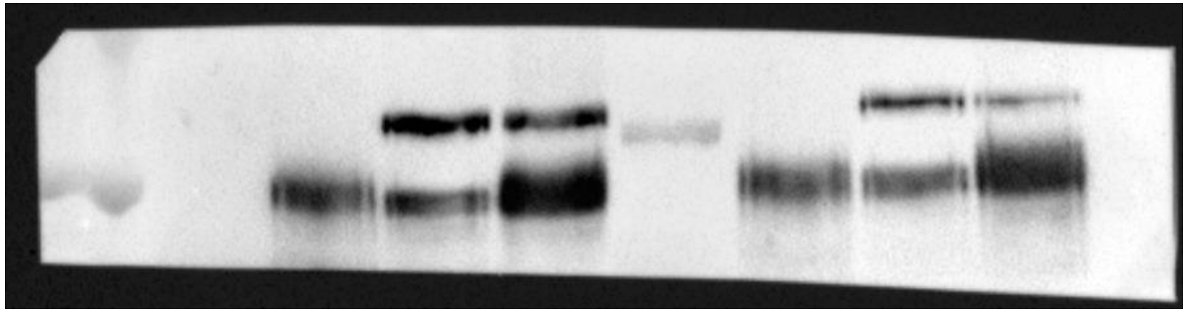

Replicate 1: U2AF2

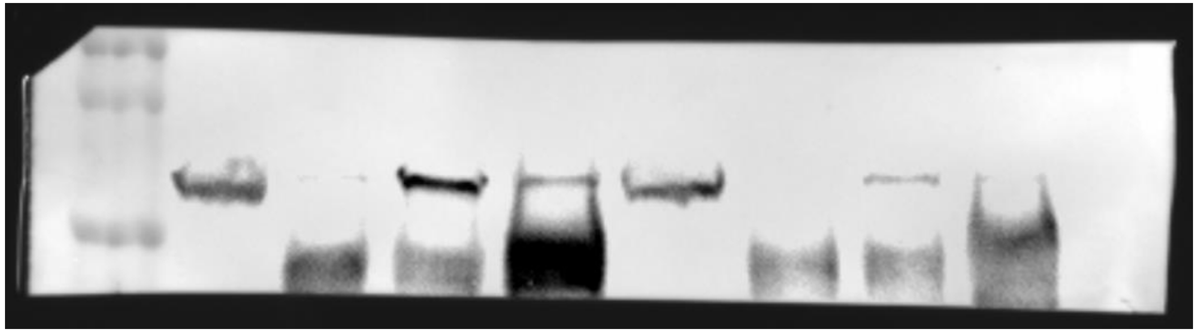

Replicate 1: SRSF1

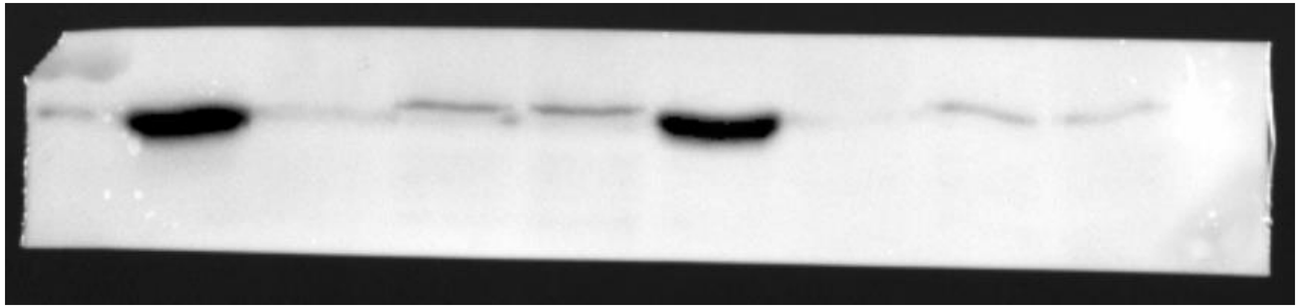

Replicate 1: GAPDH

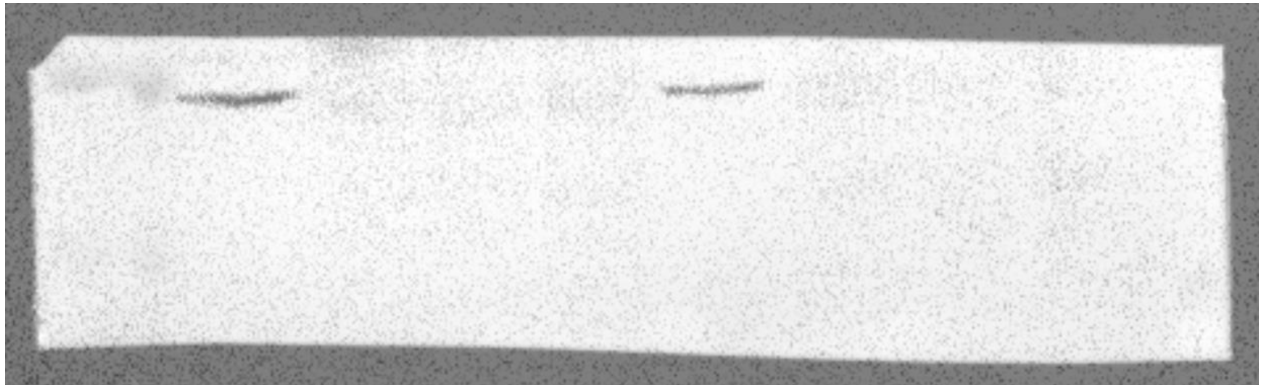

Replicate 2: LSD1

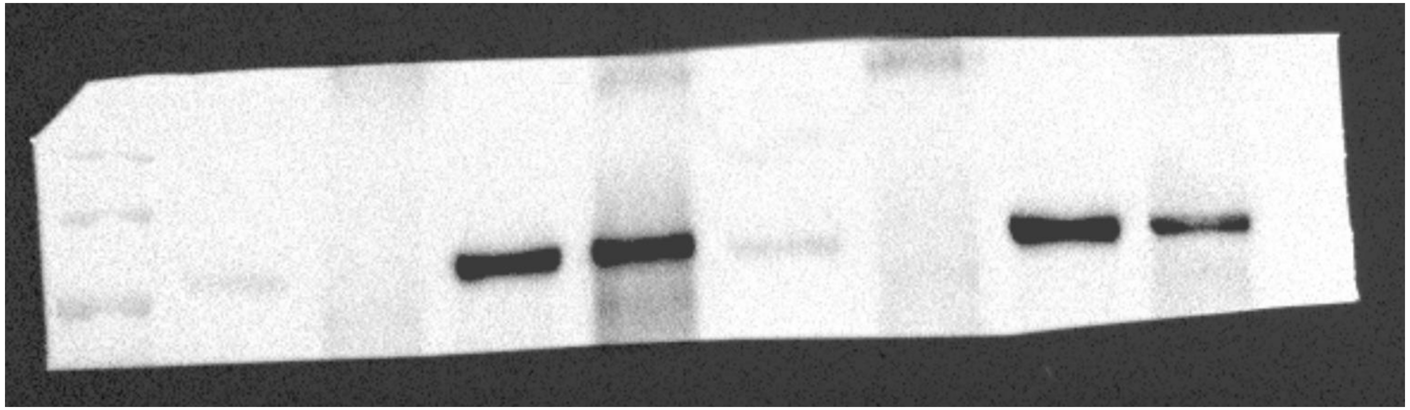

Replicate 2: RCOR1

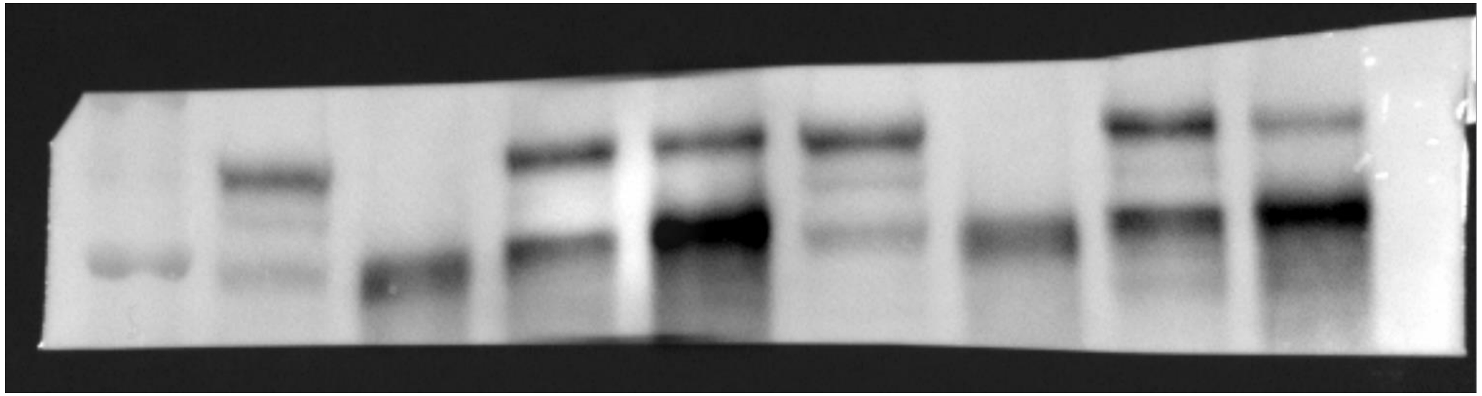

Replicate 2: U2AF2

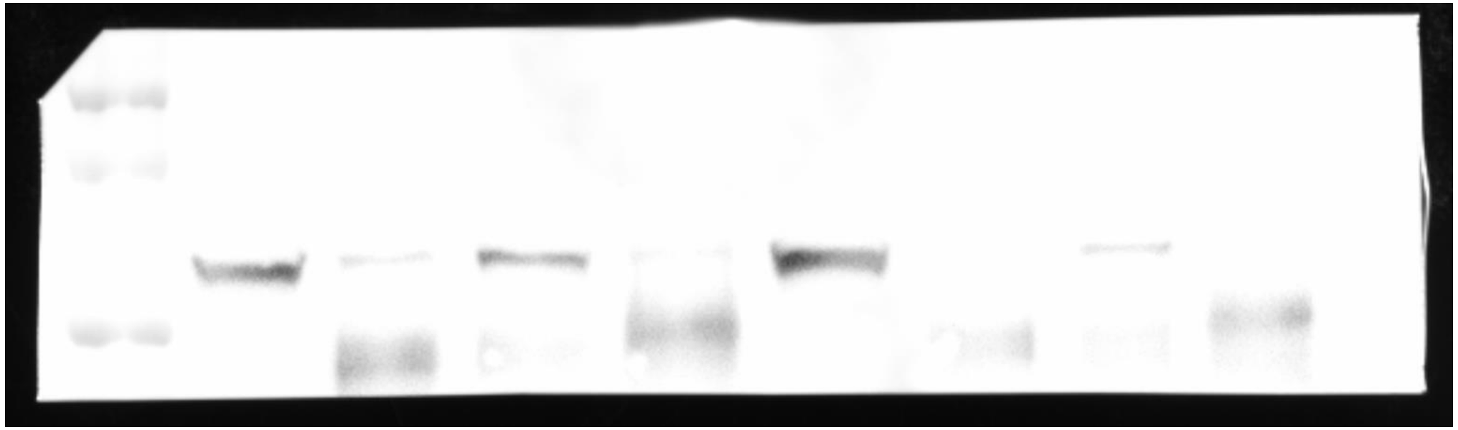

Replicate 2: SRSF1

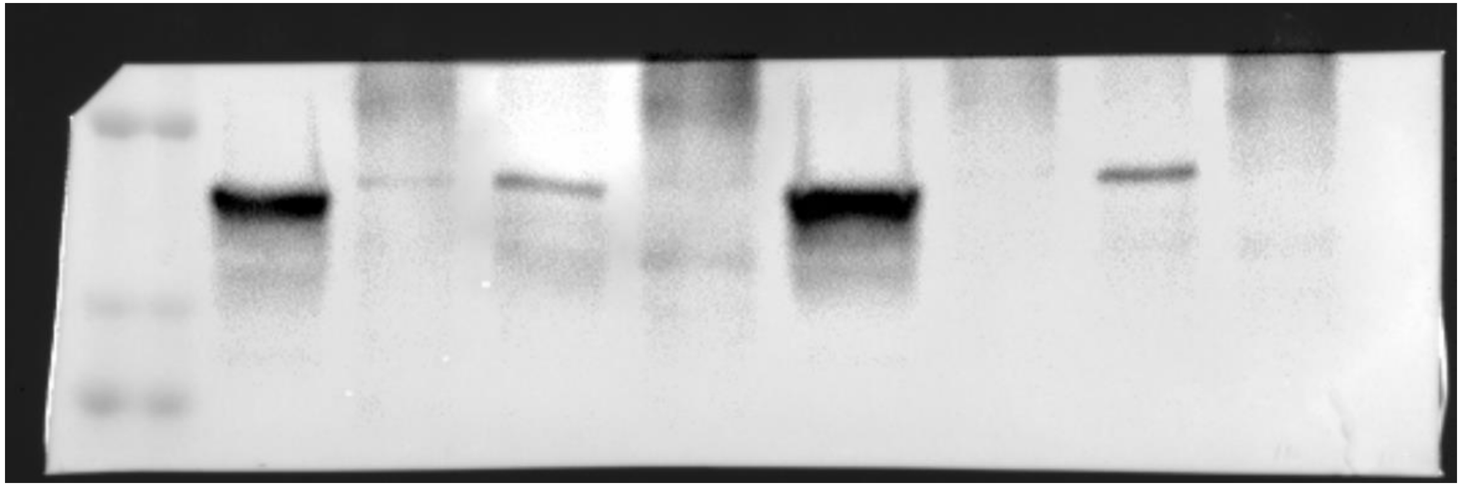

Figure 1F

Coomassie staining– Purified proteins

1. Full-length LSD1, LSD1-HDAC1-RCOR1 complex

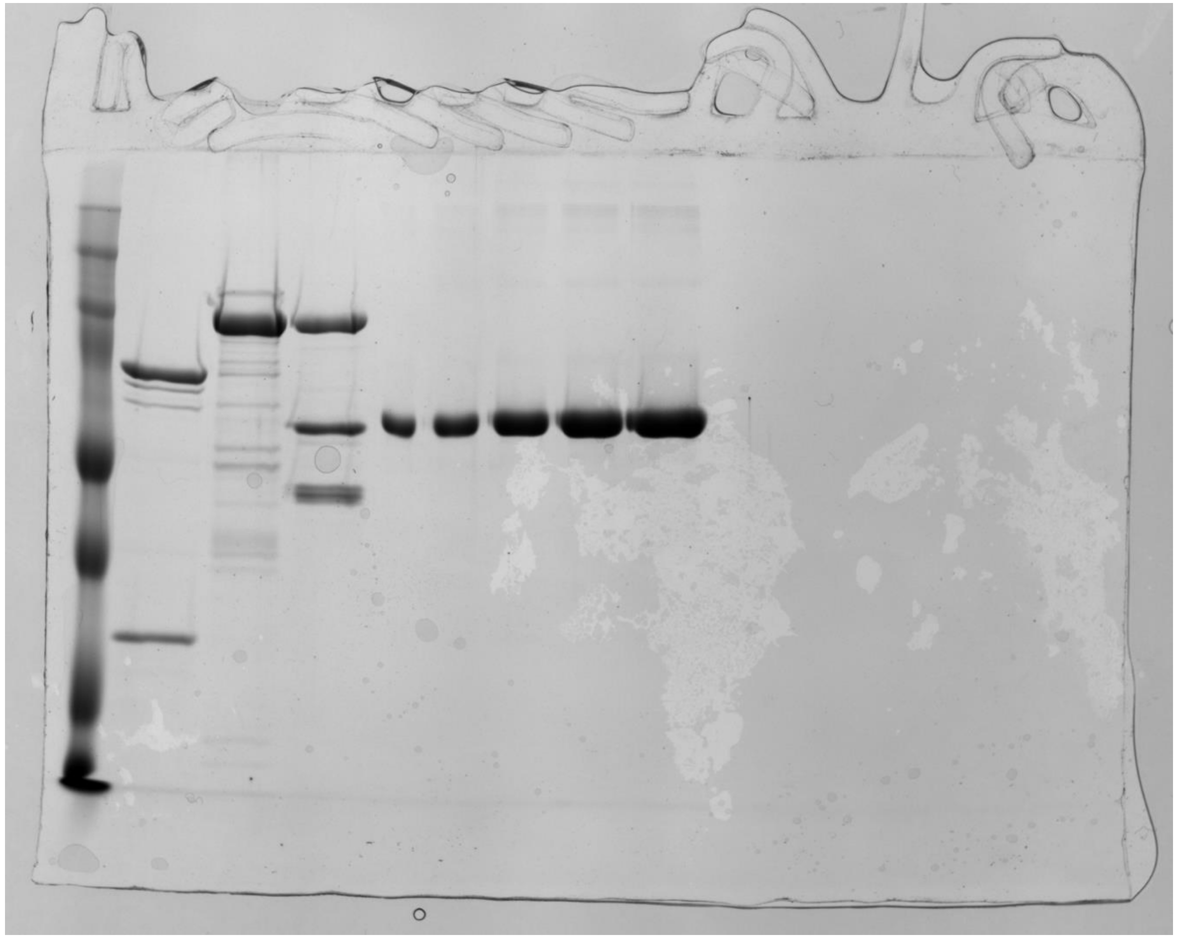

2. GST, GST-U2AF2 85-471, GST-SRSF1
3. His-LSD1 171-852/His-RCOR1 289-485, His-U2AF2 241-471

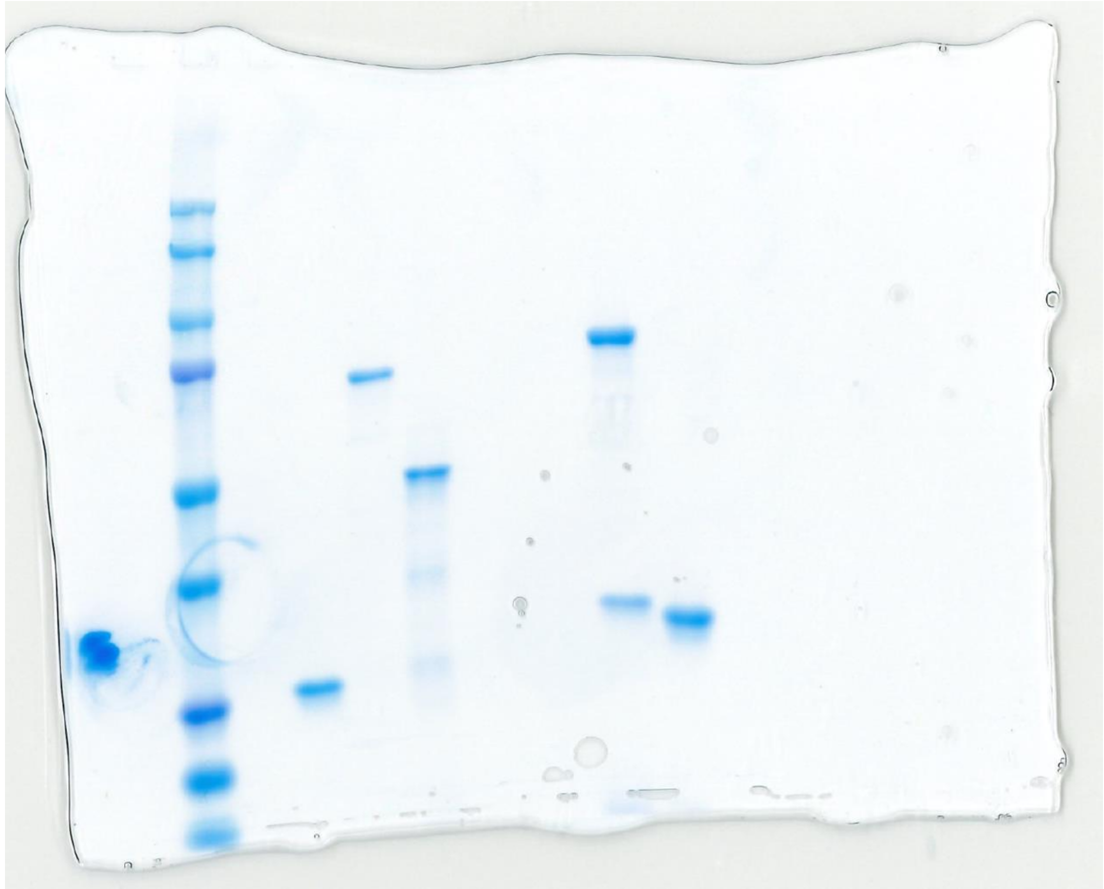

Figure 1G

Western blots – WT and U2AF2-V5 O/E SKMEL5 treated with DMSO or corin (24h, 2.5uM). Probed for LSD1, RCOR1, U2AF2, SRSF1, GAPDH

Replicate 1: LSD1

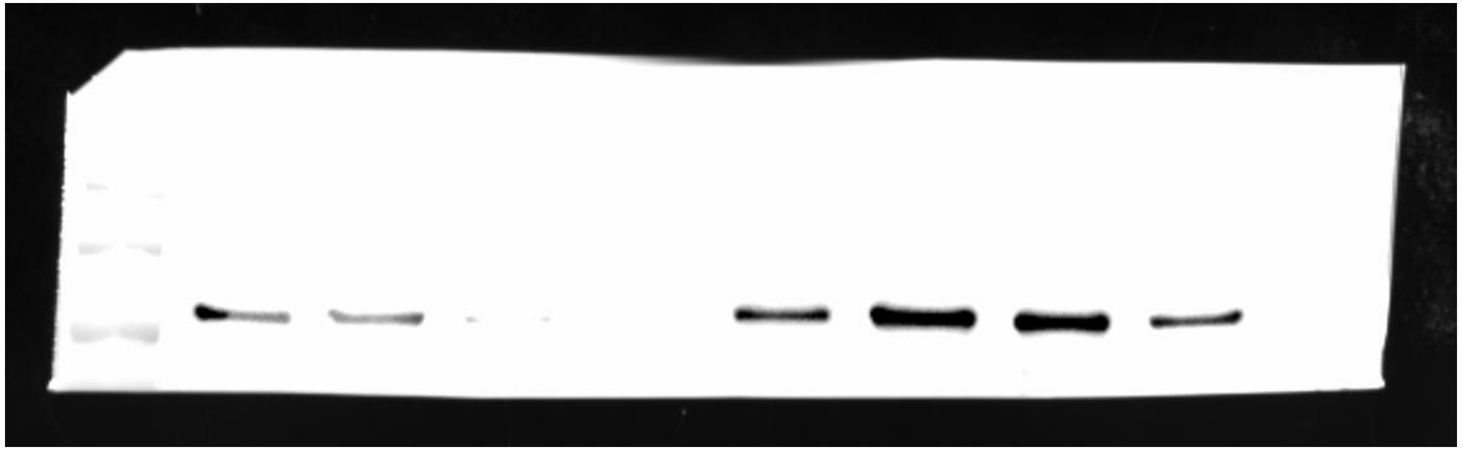

Replicate 1: HDAC1

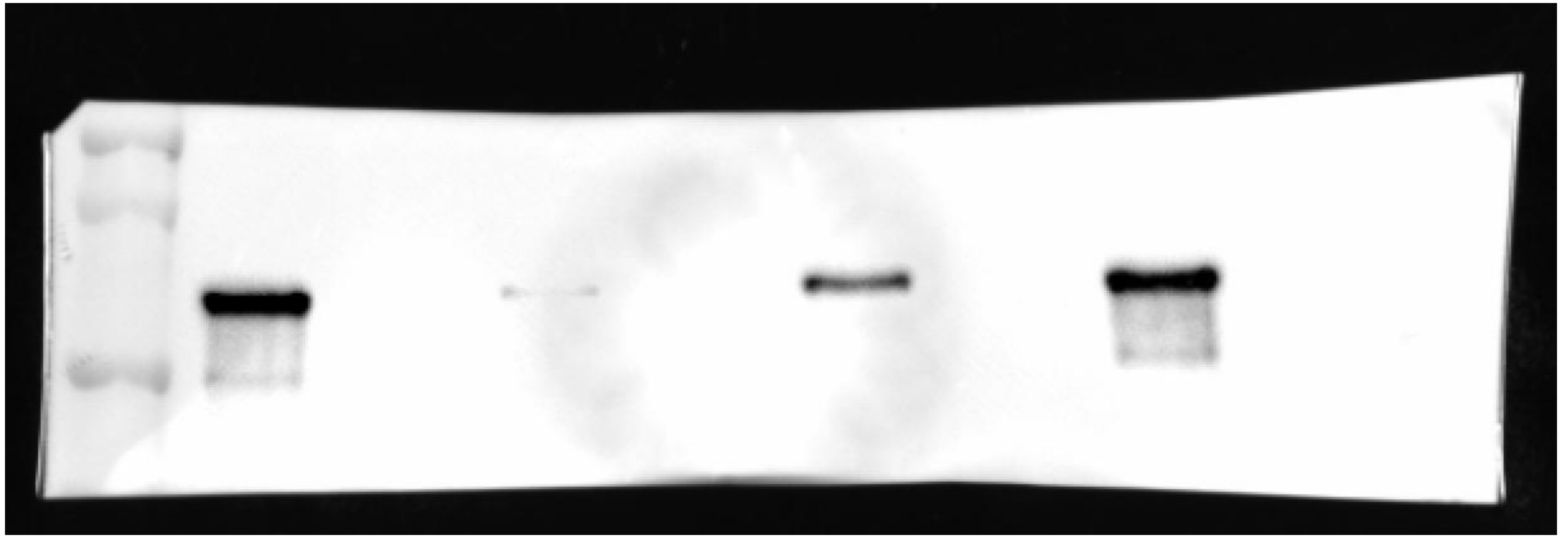

Replicate 1: GST

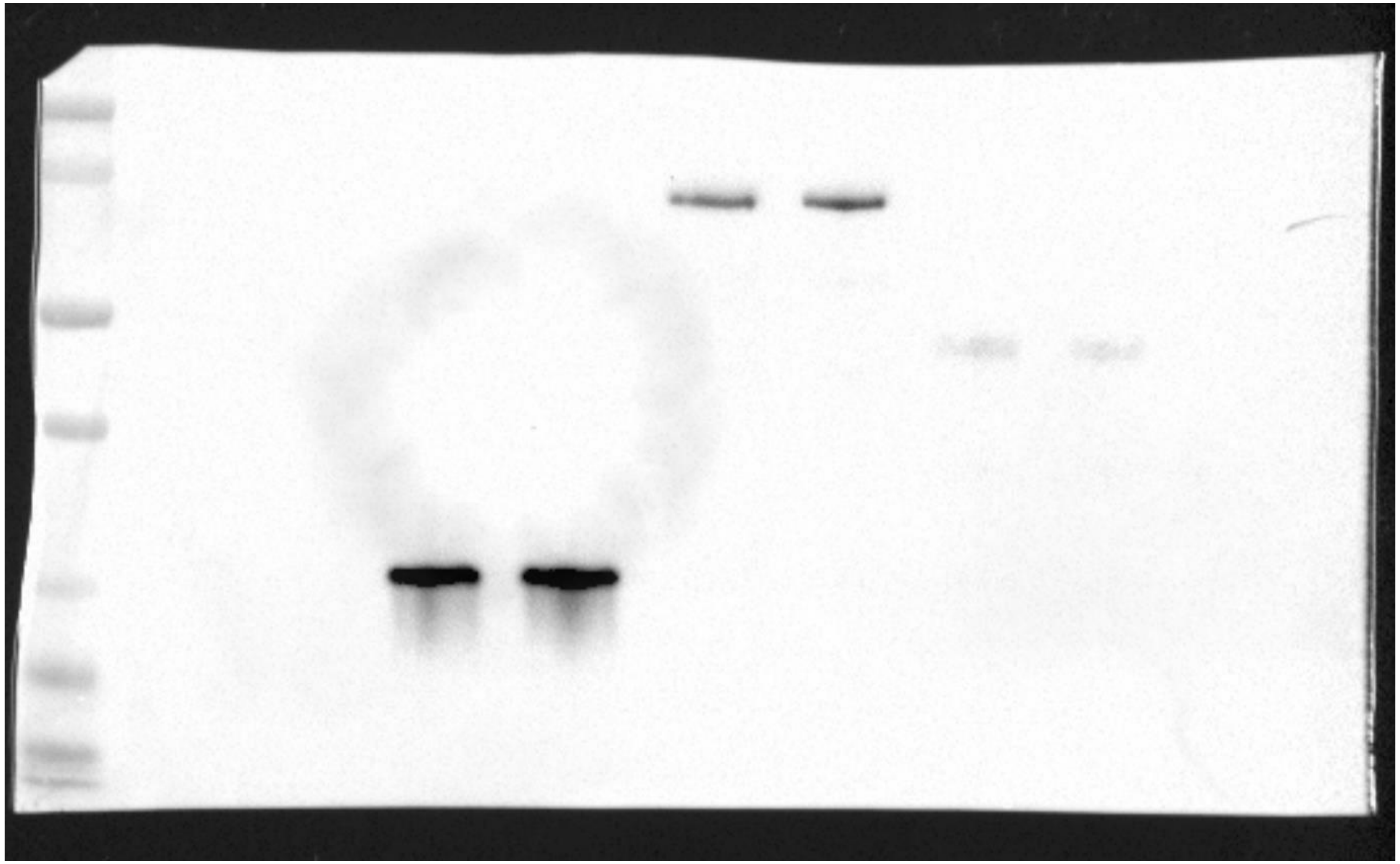

Replicate 2: LSD1

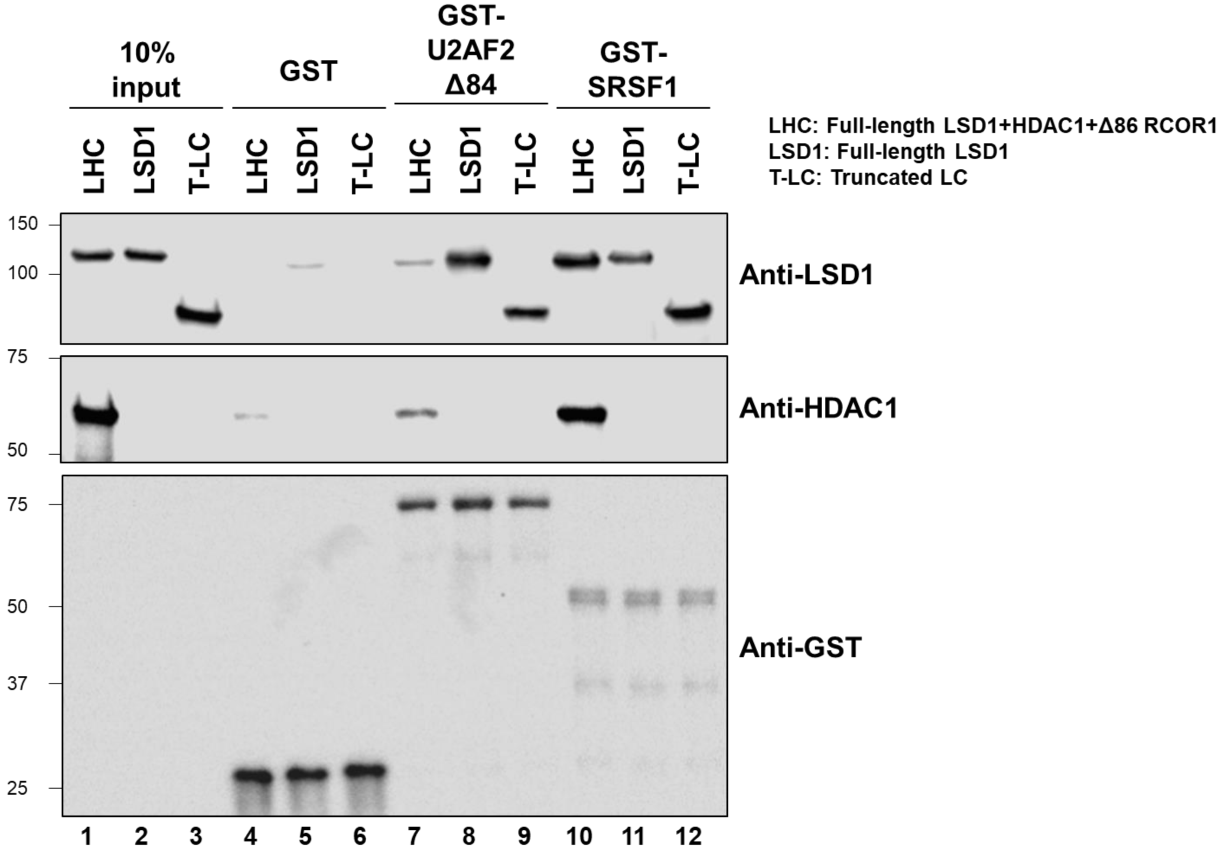

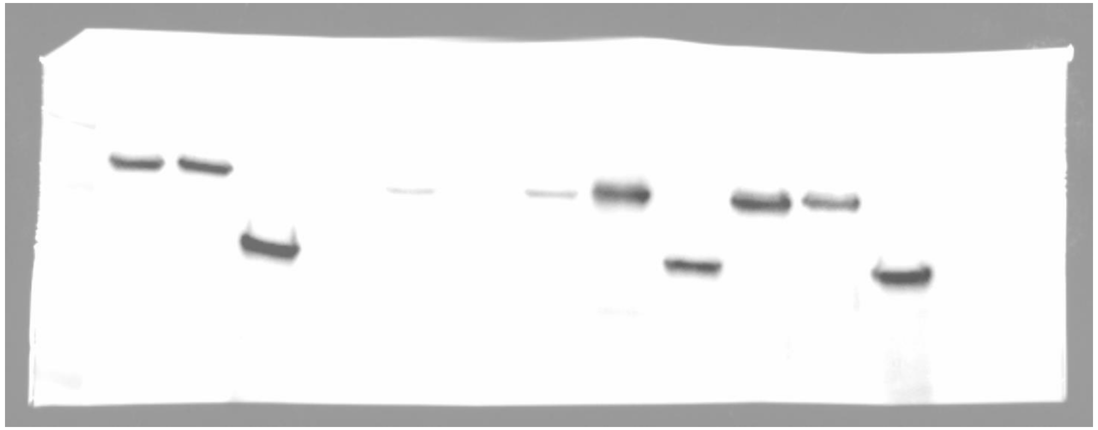

- In addition to LHS and LSD1, TR-LC is also included in this experiment

Replicate 2: HDAC1

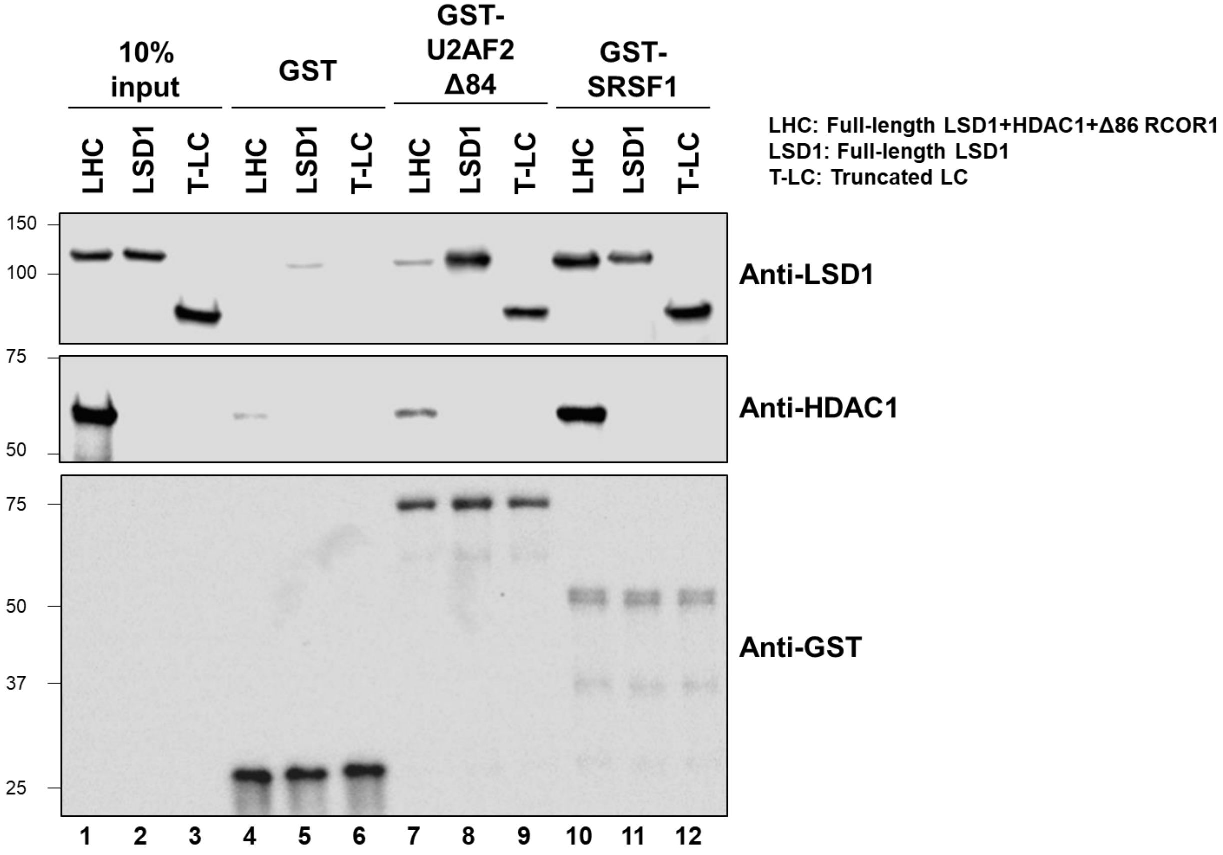

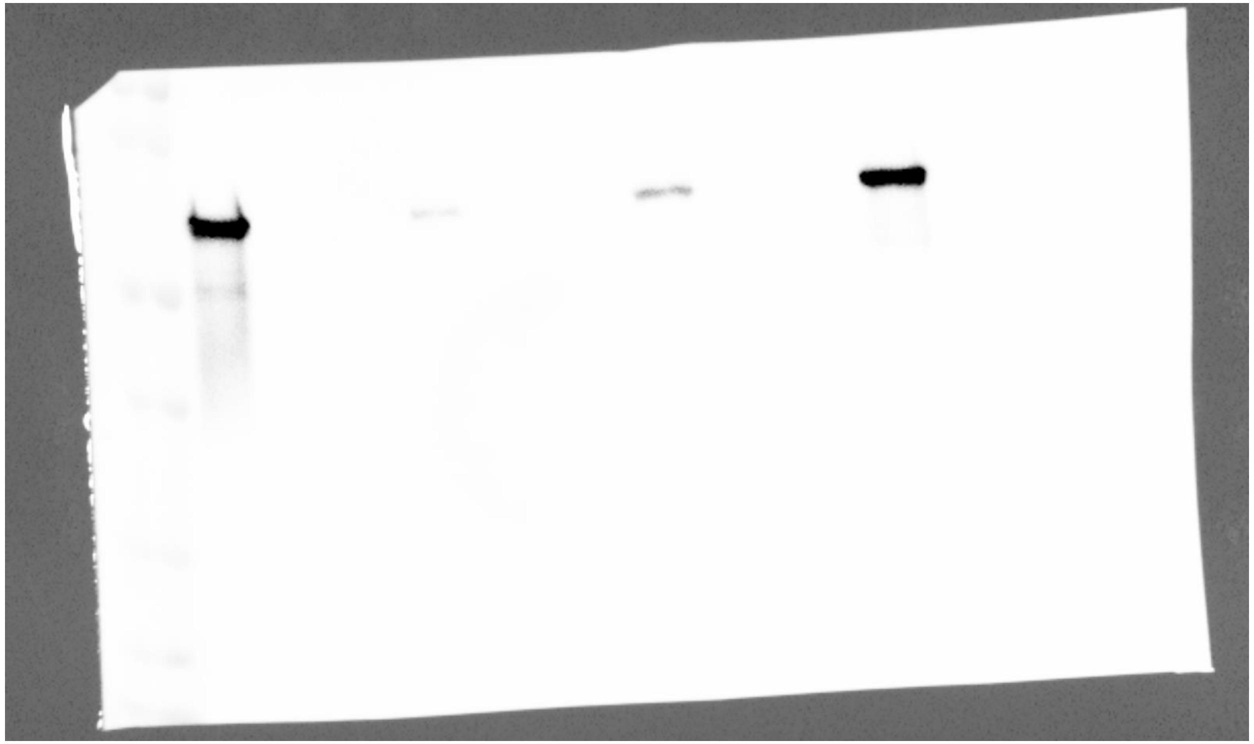

- In addition to LHS and LSD1, TR-LC is also included in this experiment

Replicate 2: GST

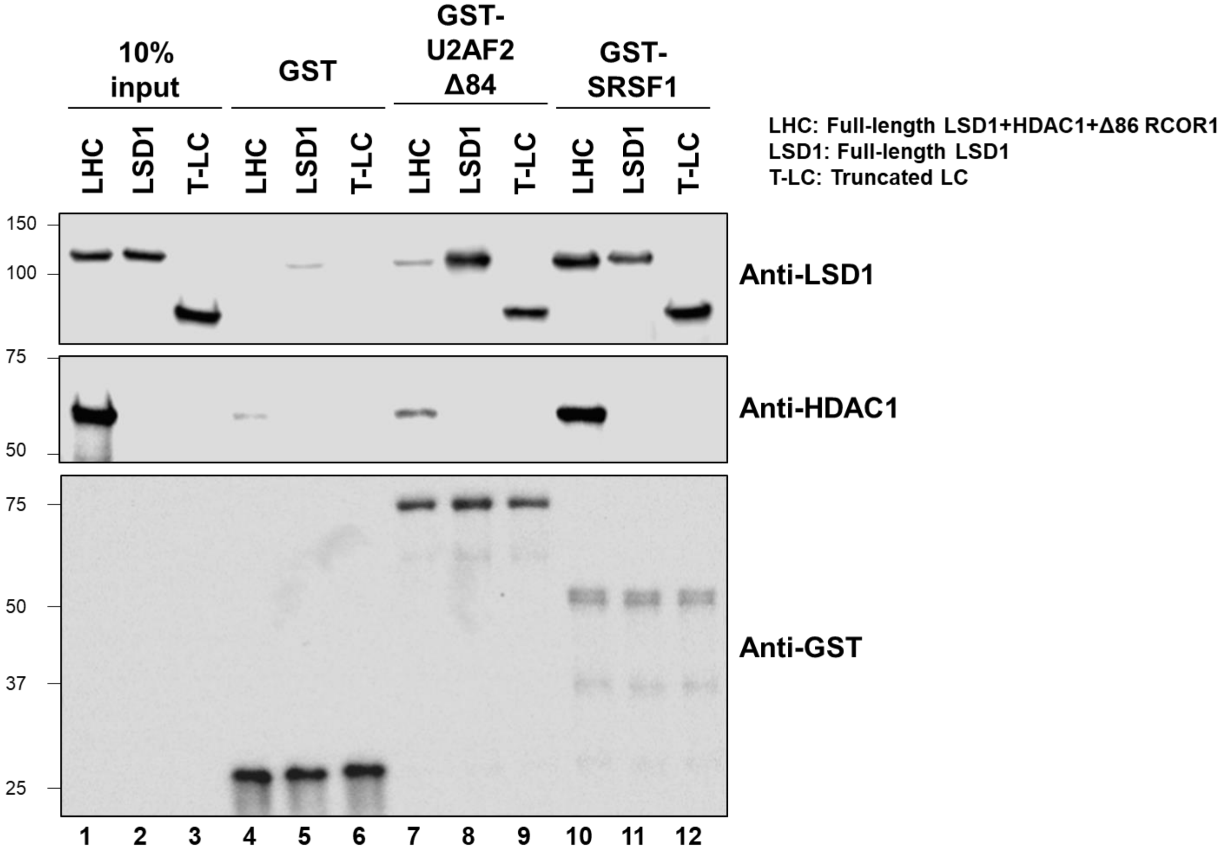

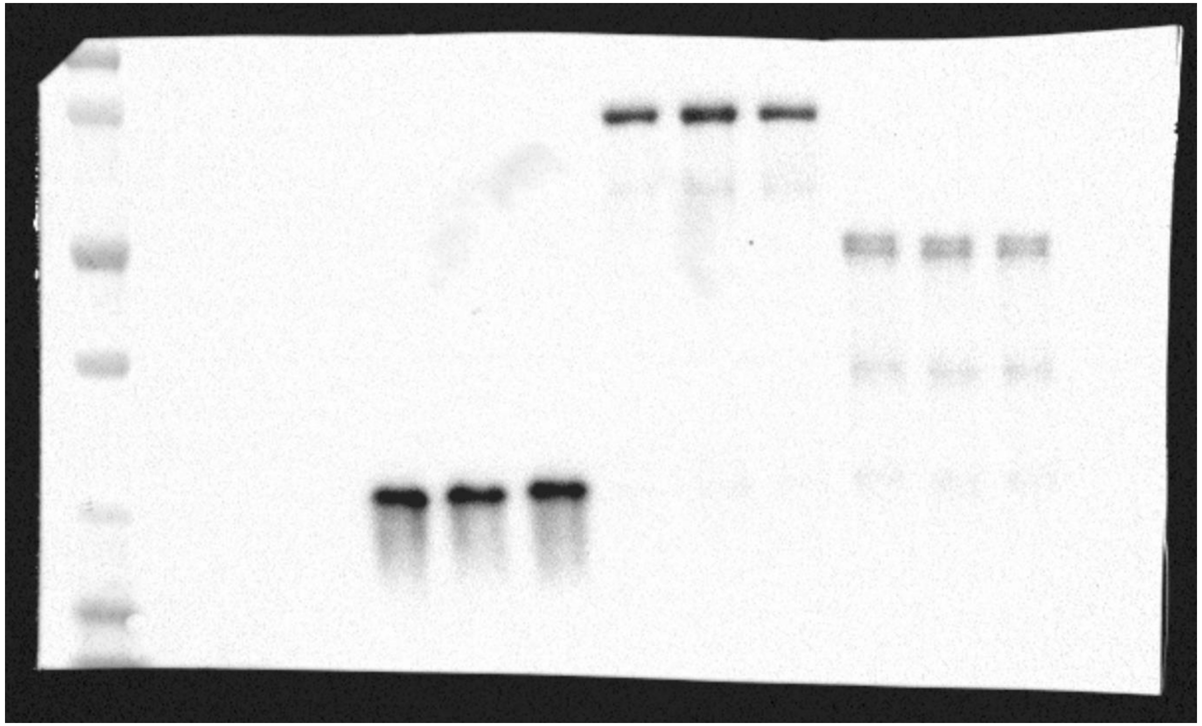

- In addition to LHS and LSD1, TR-LC is also included in this experiment

Figure 1H

Coomassie staining-Size exclusion of LSD1-RCOR1-U2AF2 for Cryo-EM sample preparation

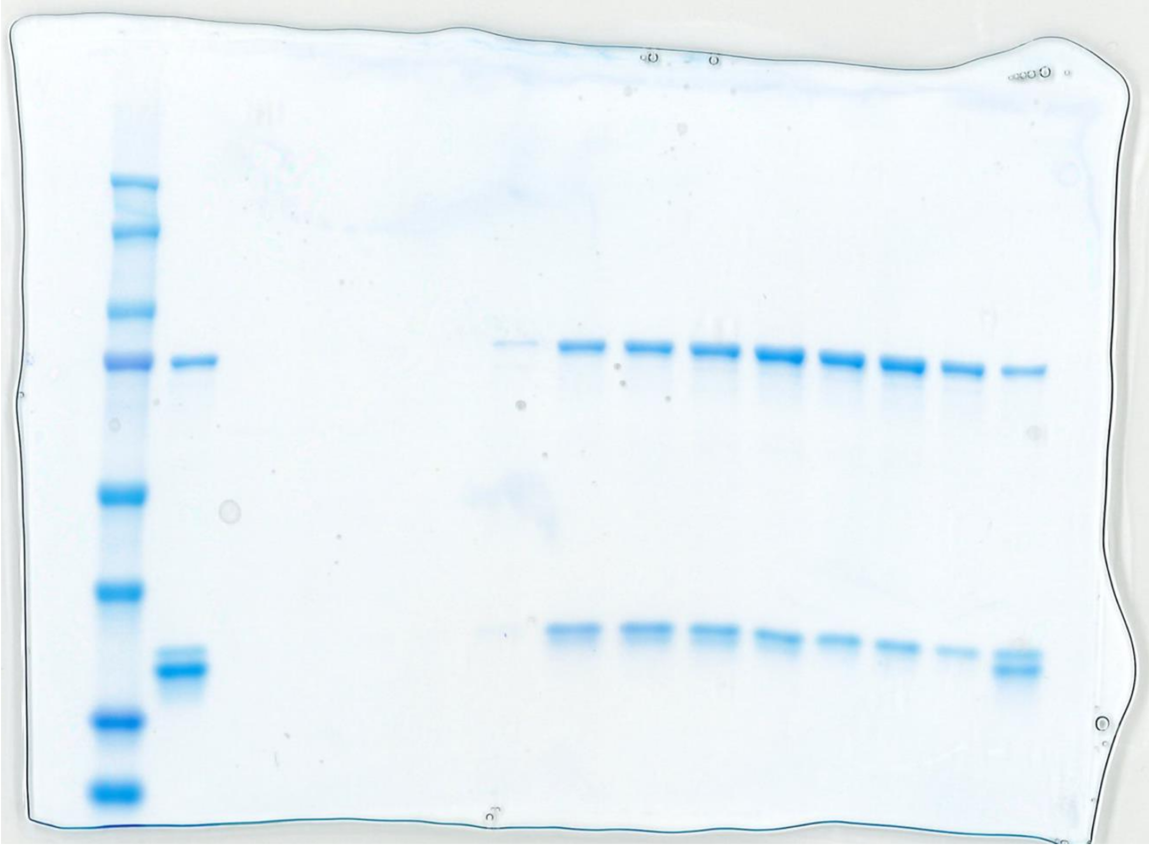
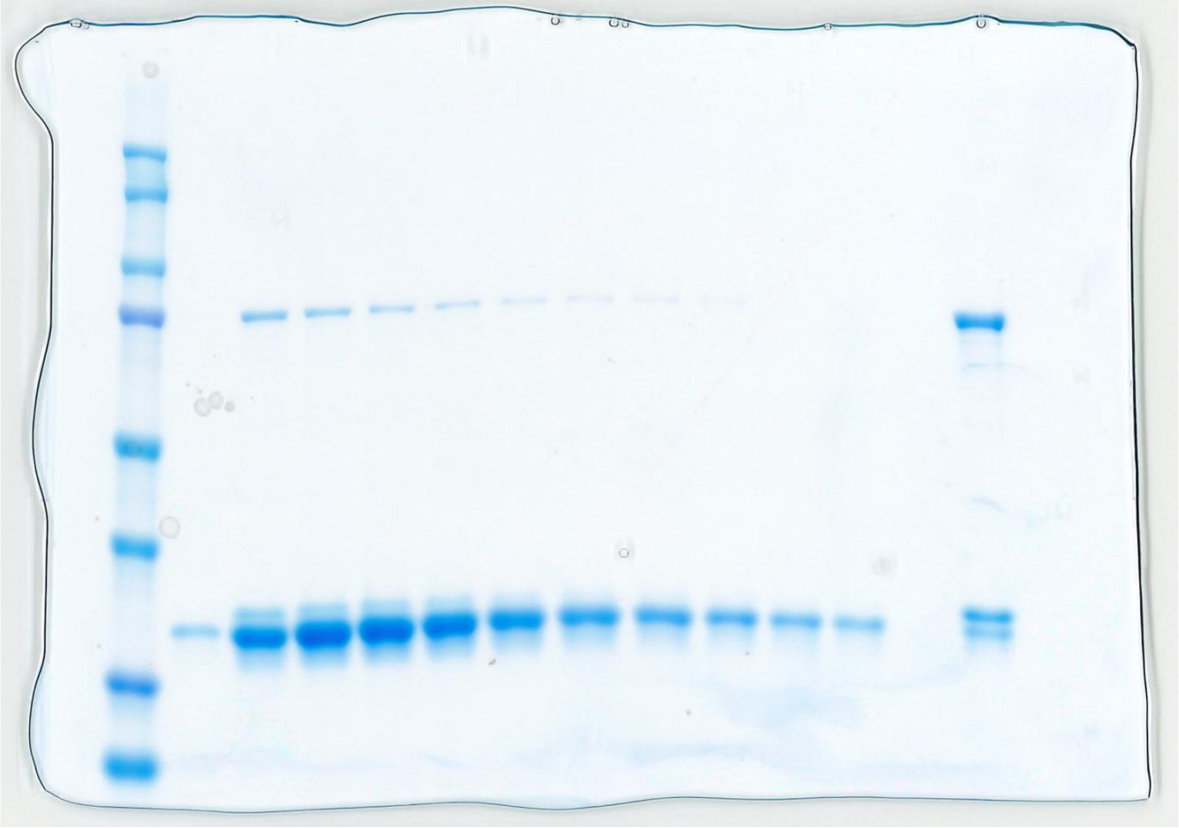

Figure 2D, Extended Figure 2B

Western blots – 6 cell lines treated with DMSO or corin (24h, 2.5uM). Probed for U2AF2, ALYREF, RBMX, and GAPDH

Replicate 1: ALYREF

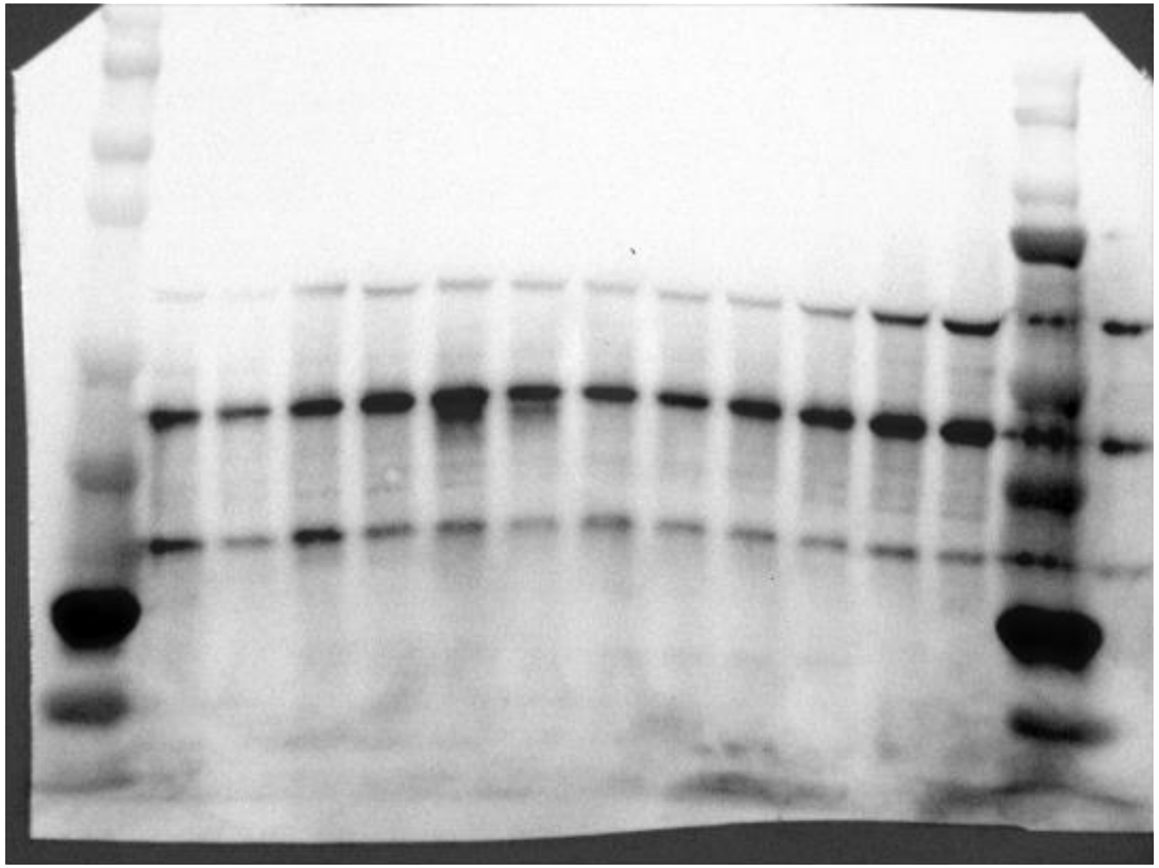

Replicate 1: U2AF2

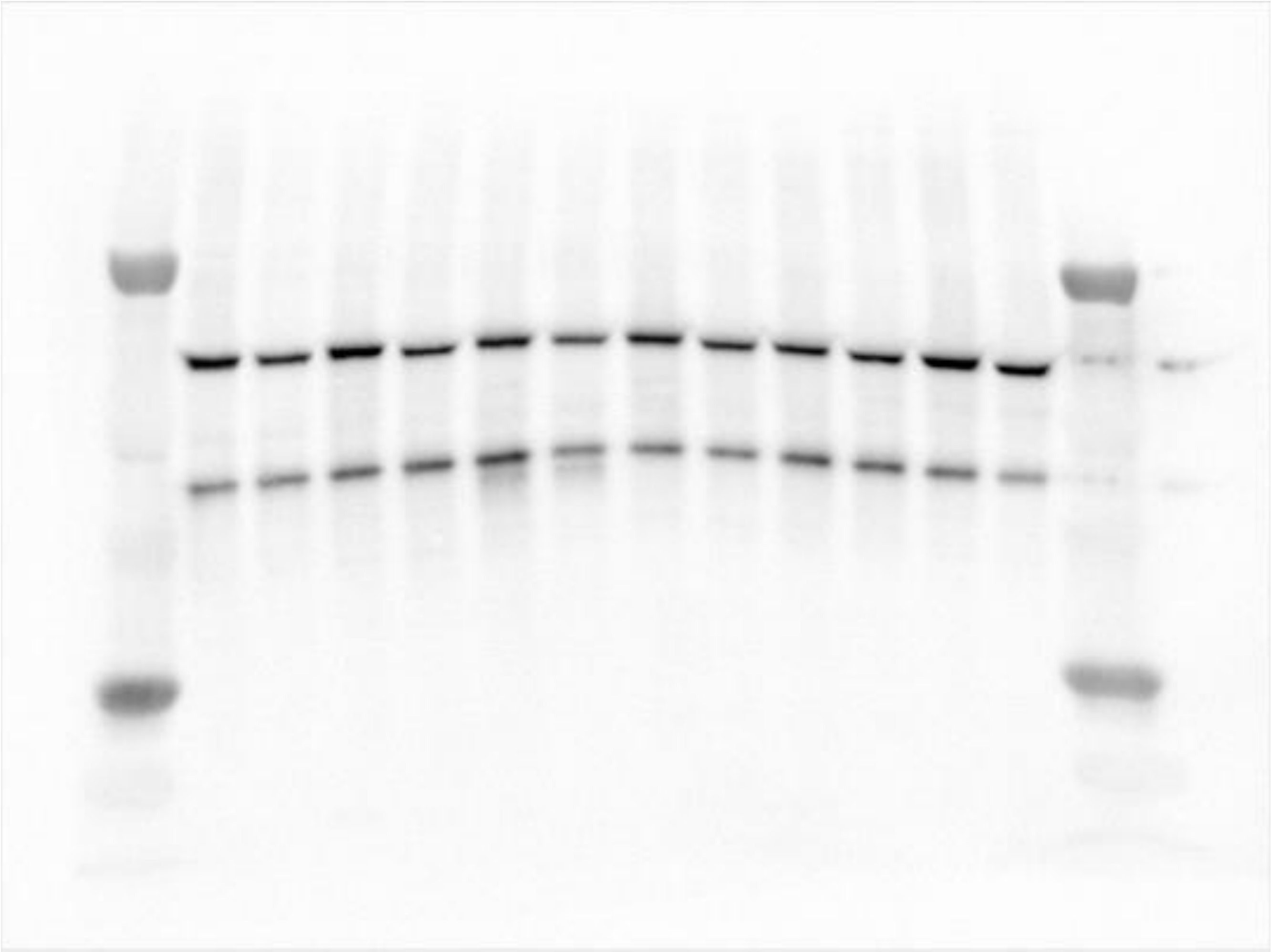

Replicate 1: RBMX

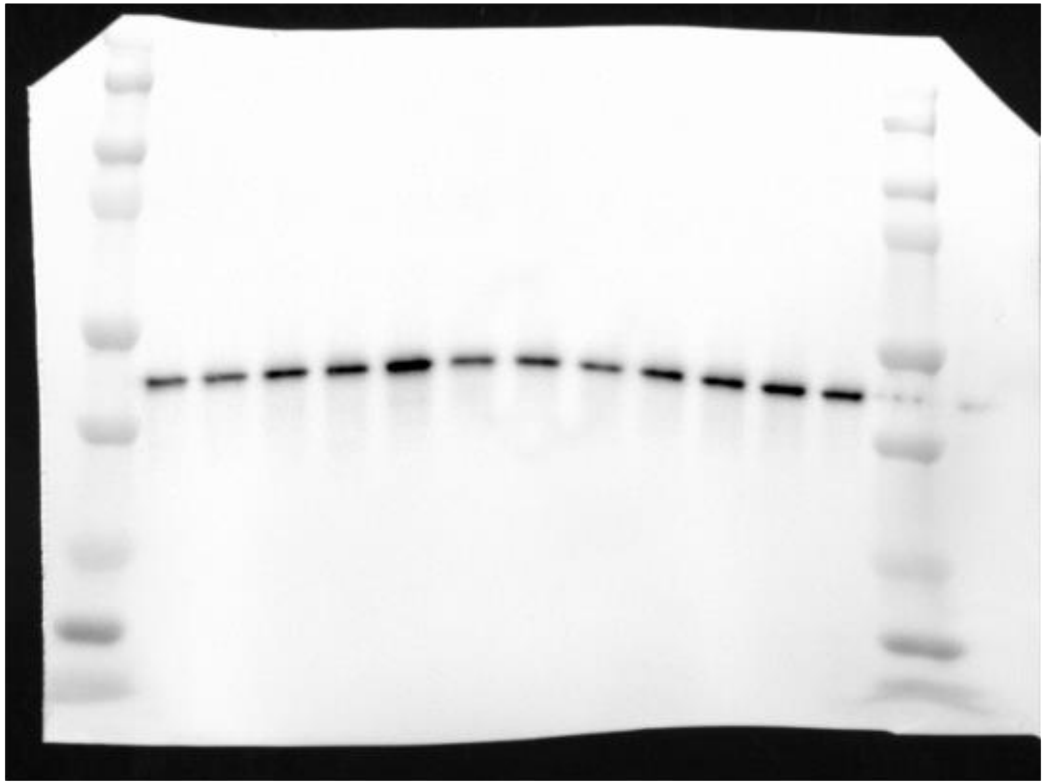

Replicate 1: GAPDH

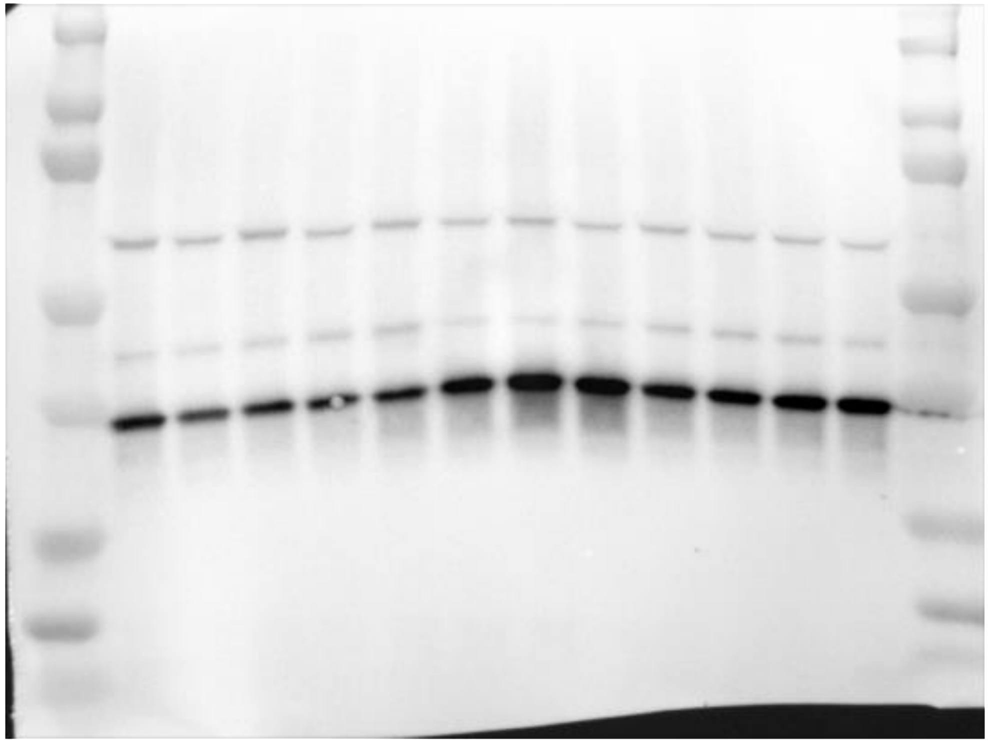

Replicate 2: ALYREF

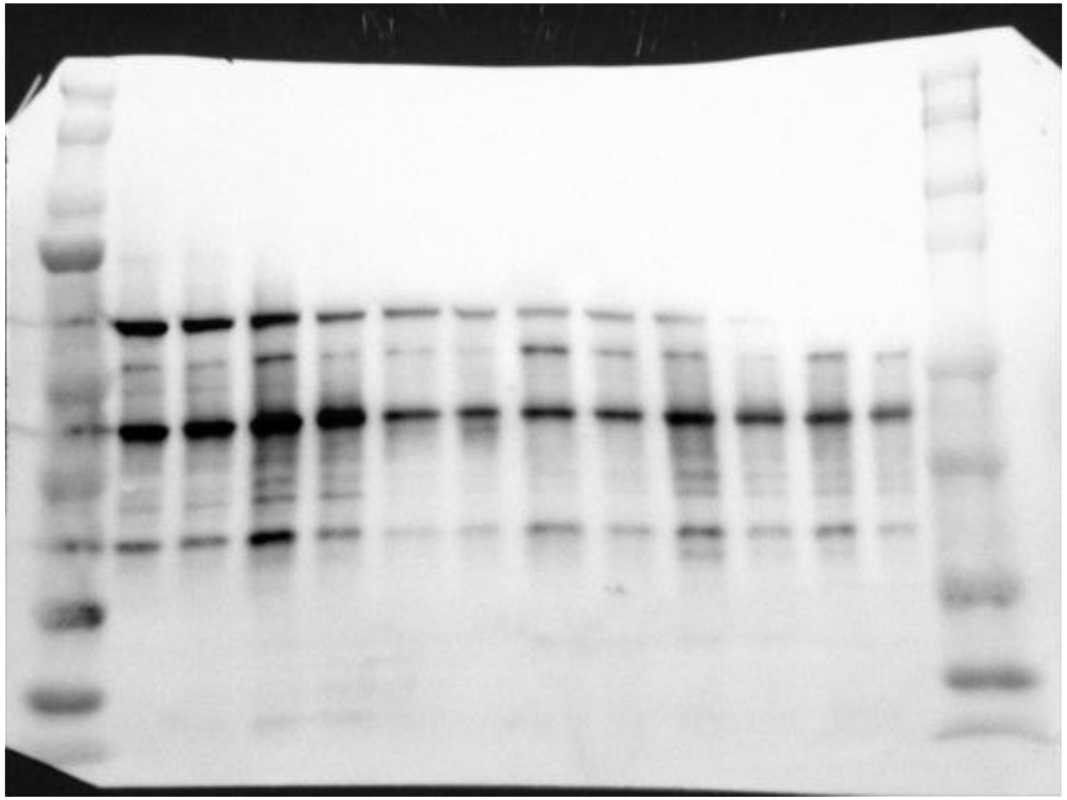

Replicate 2: U2AF2

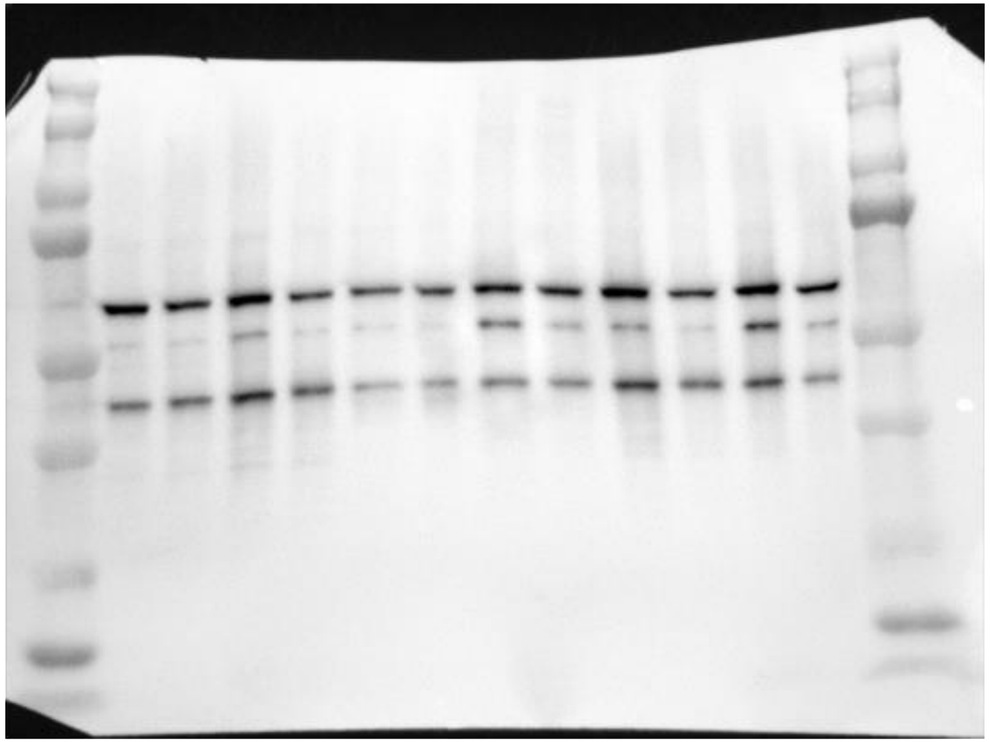

Replicate 2: RBMX

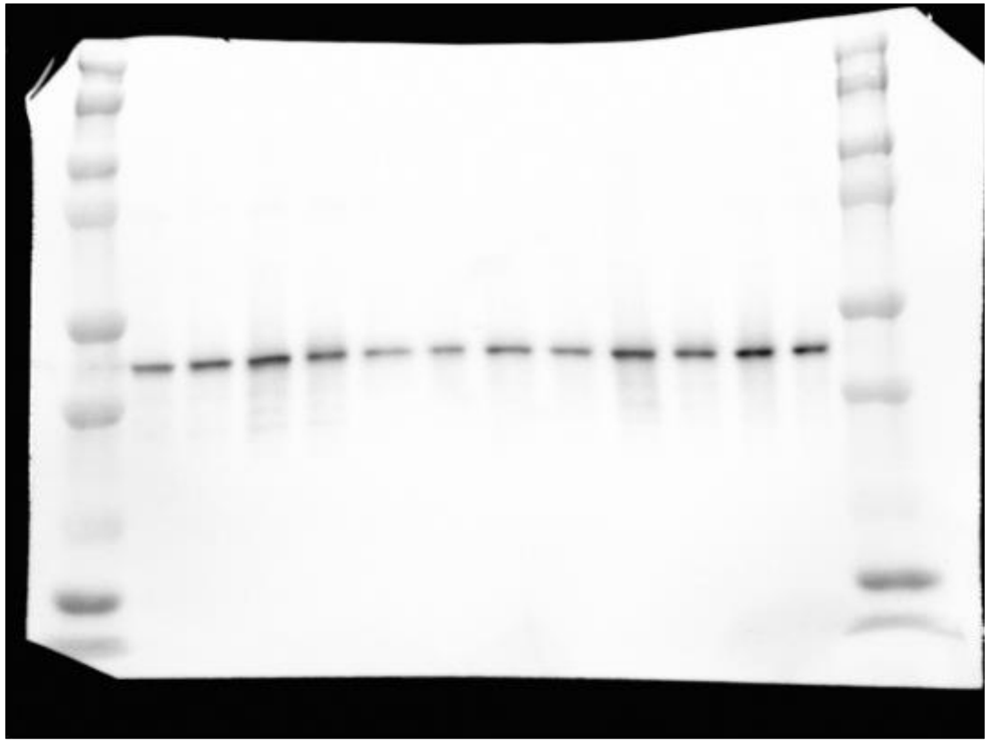

Replicate 2: GAPDH

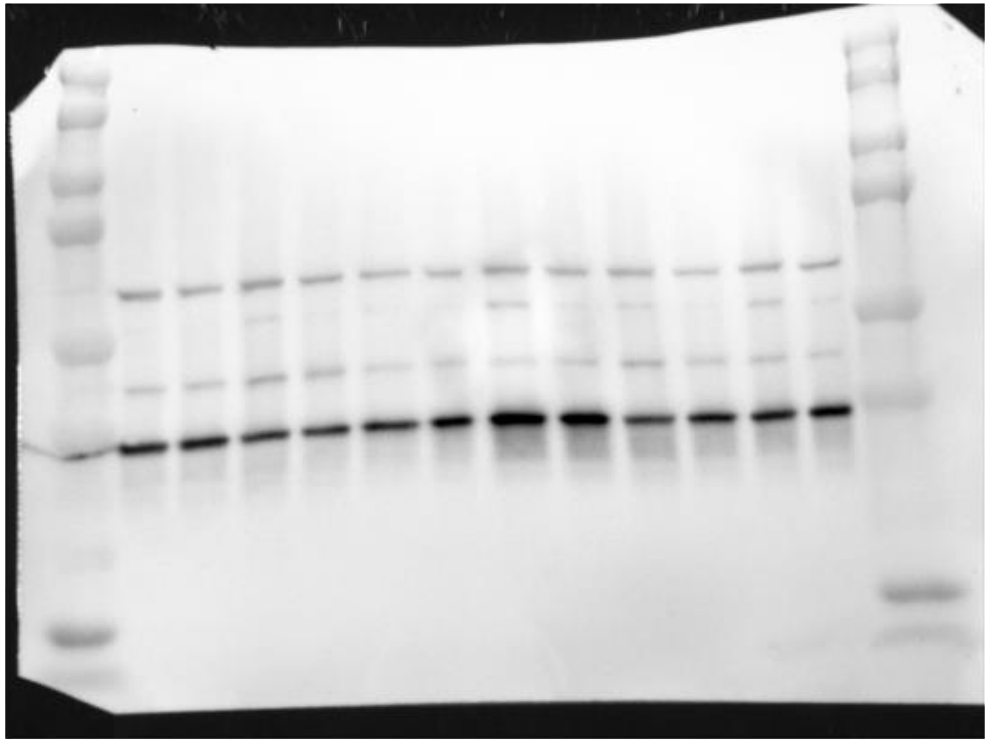

Replicate 3: ALYREF

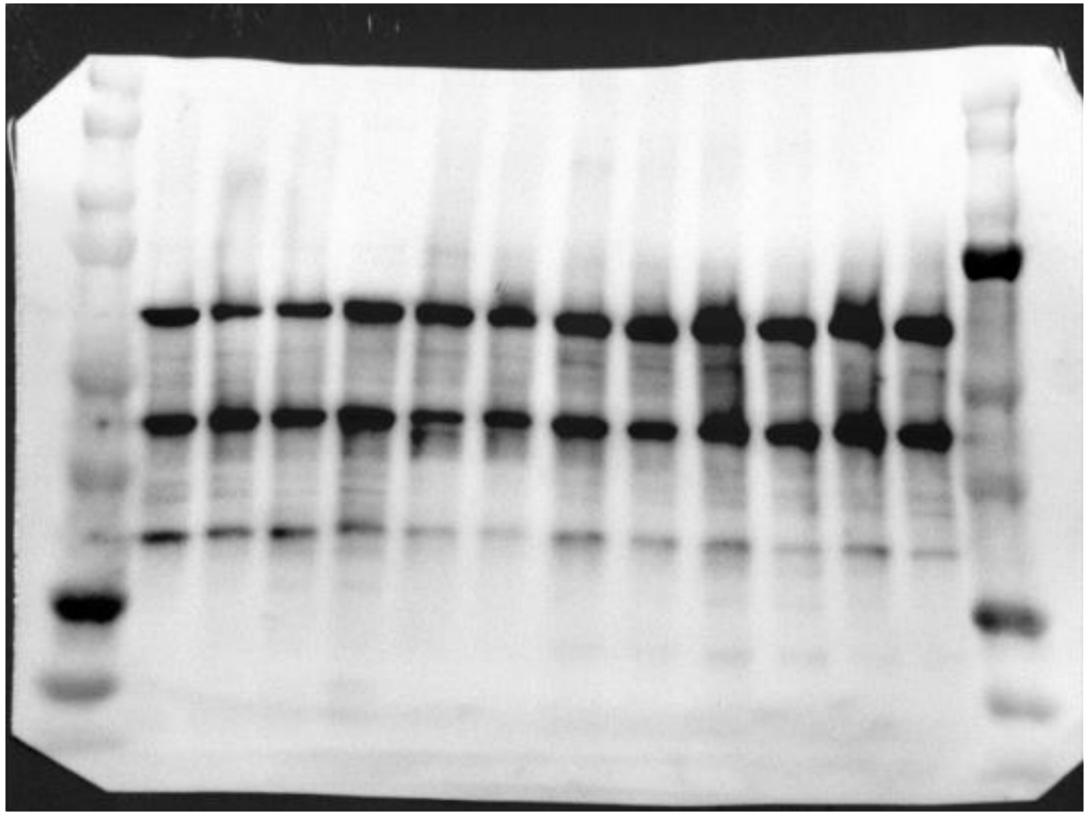

Replicate 3: U2AF2

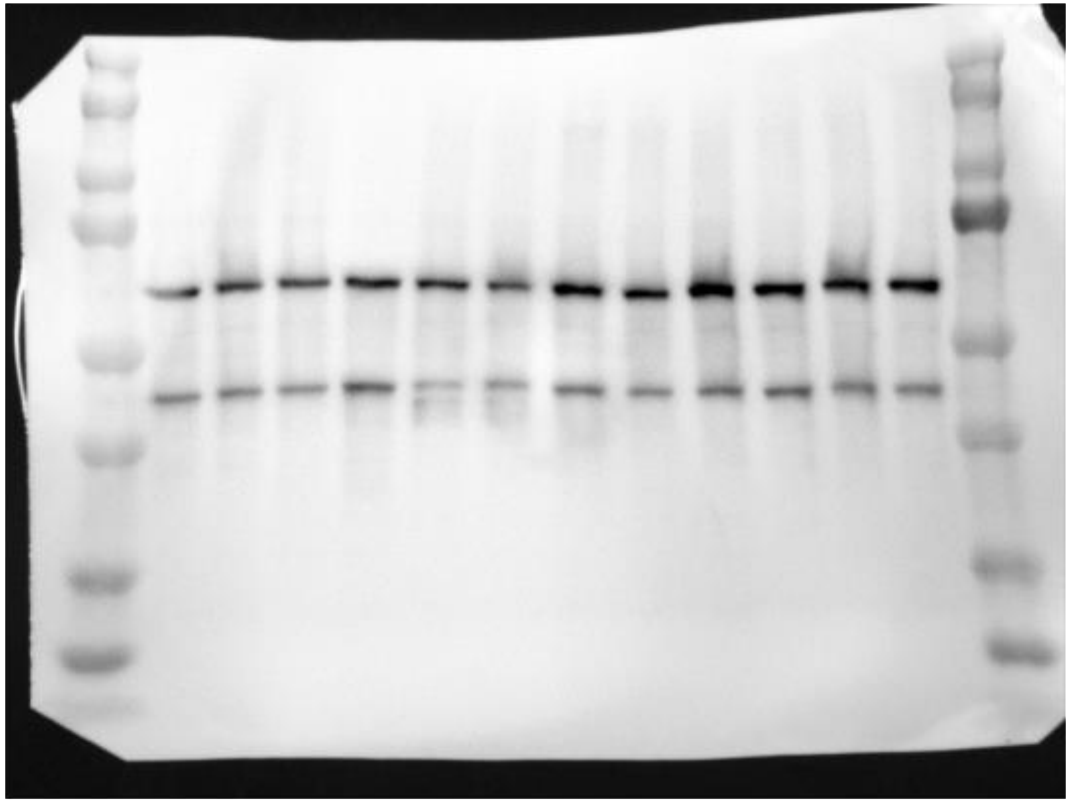

Replicate 3: RBMX

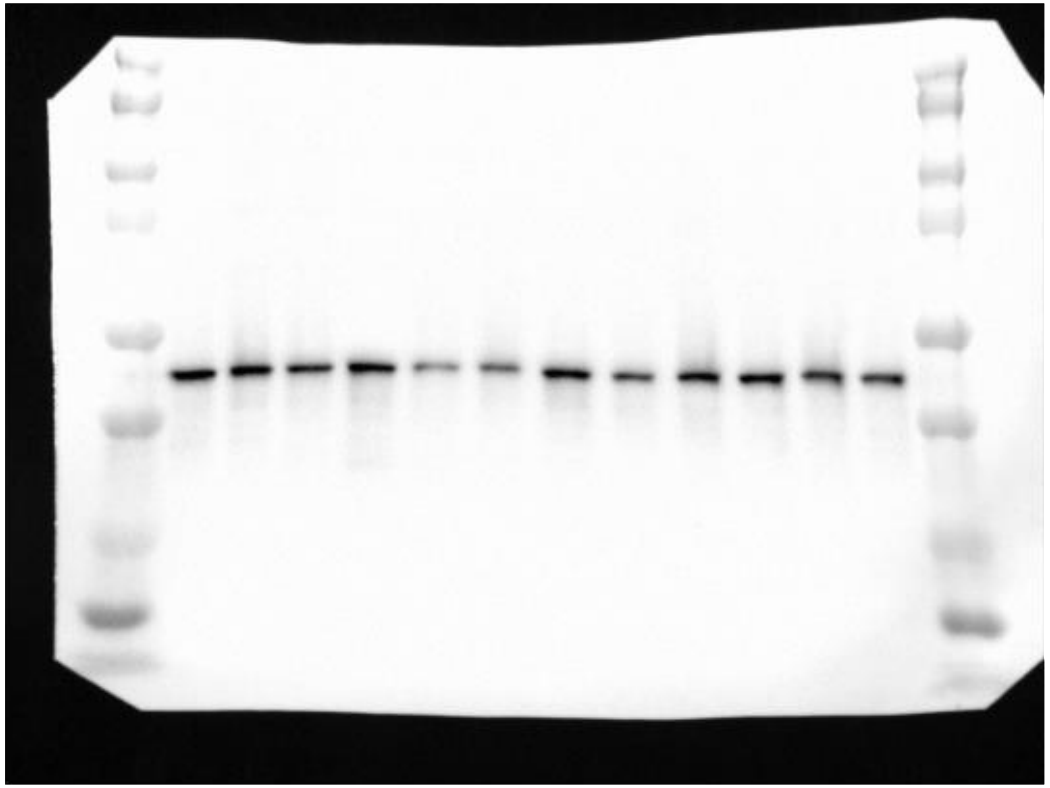

Replicate 3: GAPDH

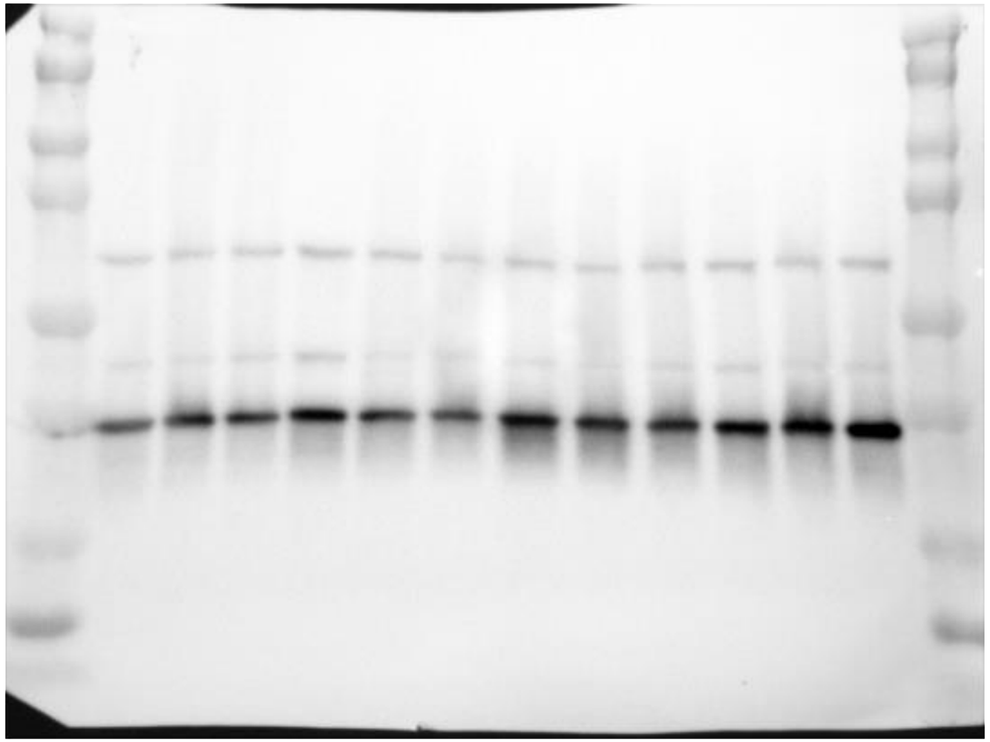

Figure 2G

Pull-down assay – Purified proteins. Probed for LSD1, RCOR1, V5, U2AF2

Replicate 1: LSD1

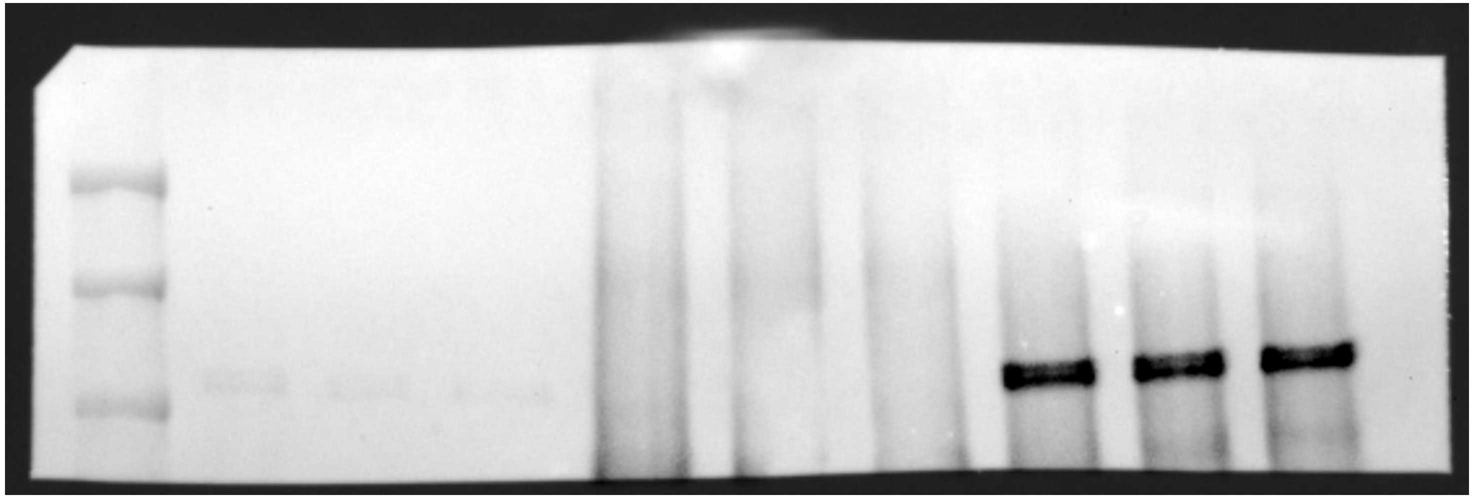

Replicate 1: RCOR1

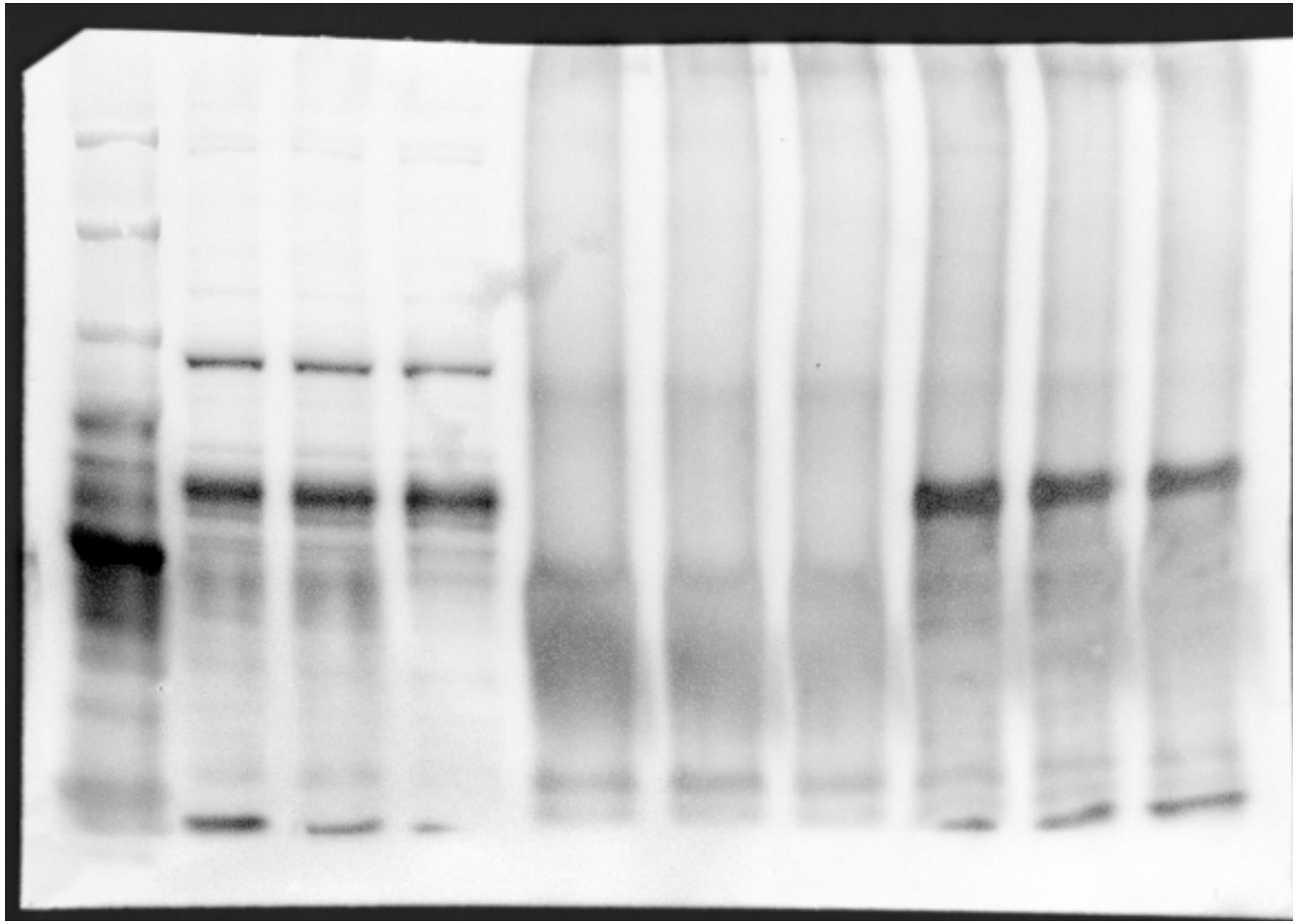

Replicate 1: V5

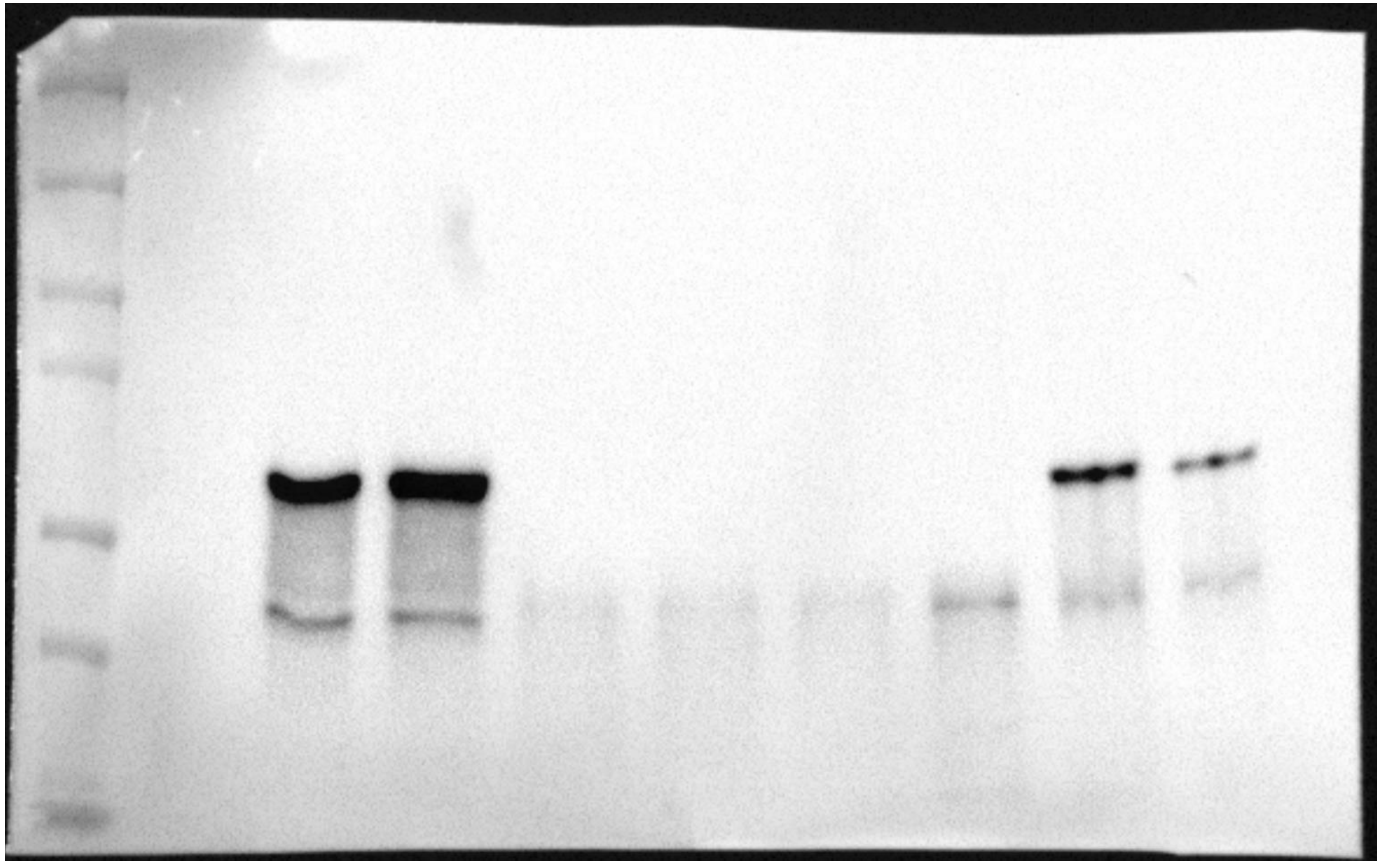

Replicate 1: U2AF2

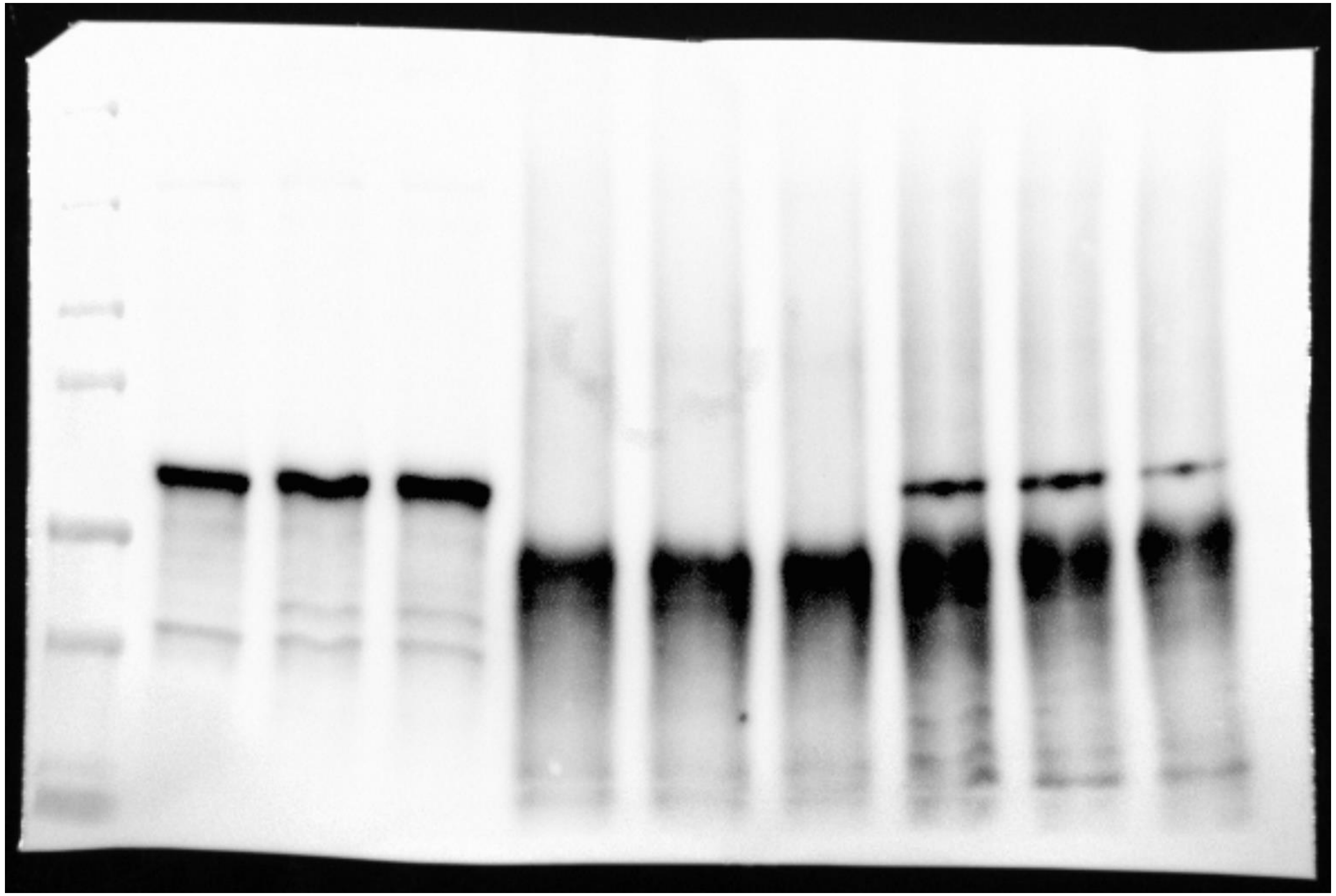

Replicate 2: RCOR1

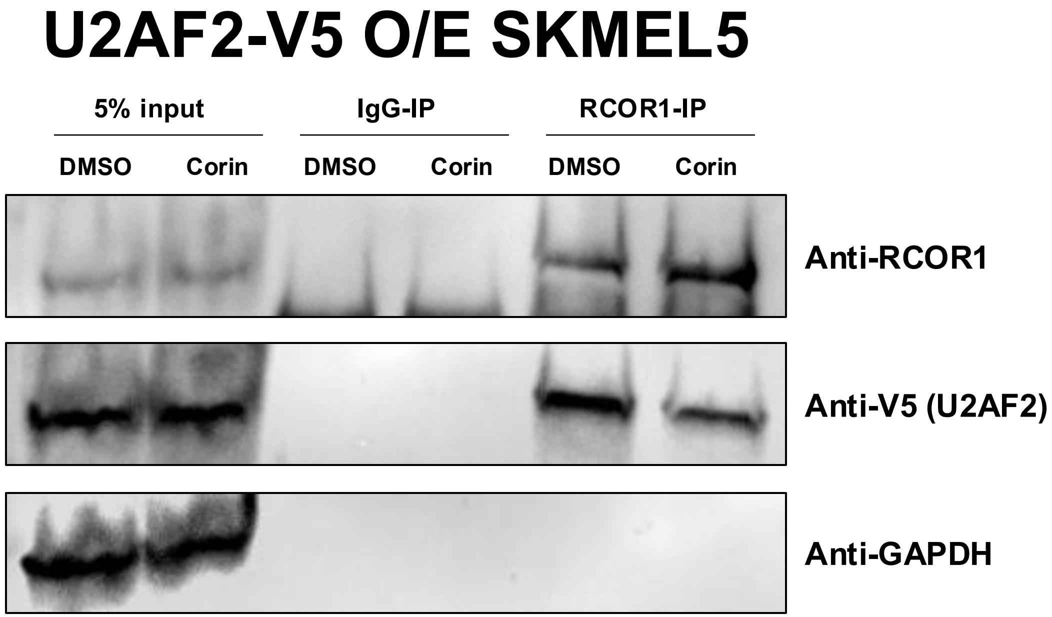

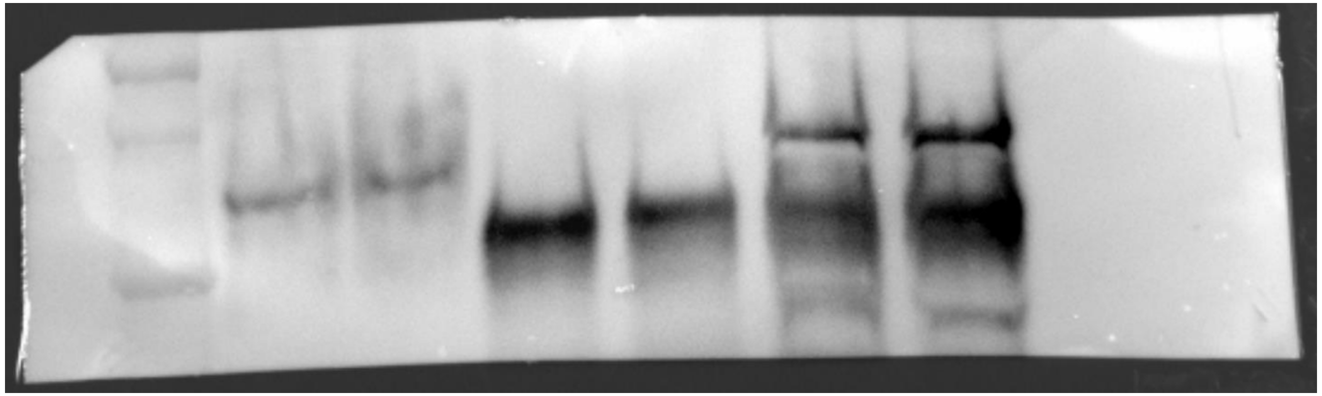

- In this experiment, only U2AF2-V5 O/E SKMEL5 cells are used, excluding WT SKMEL5.

Replicate 2: V5

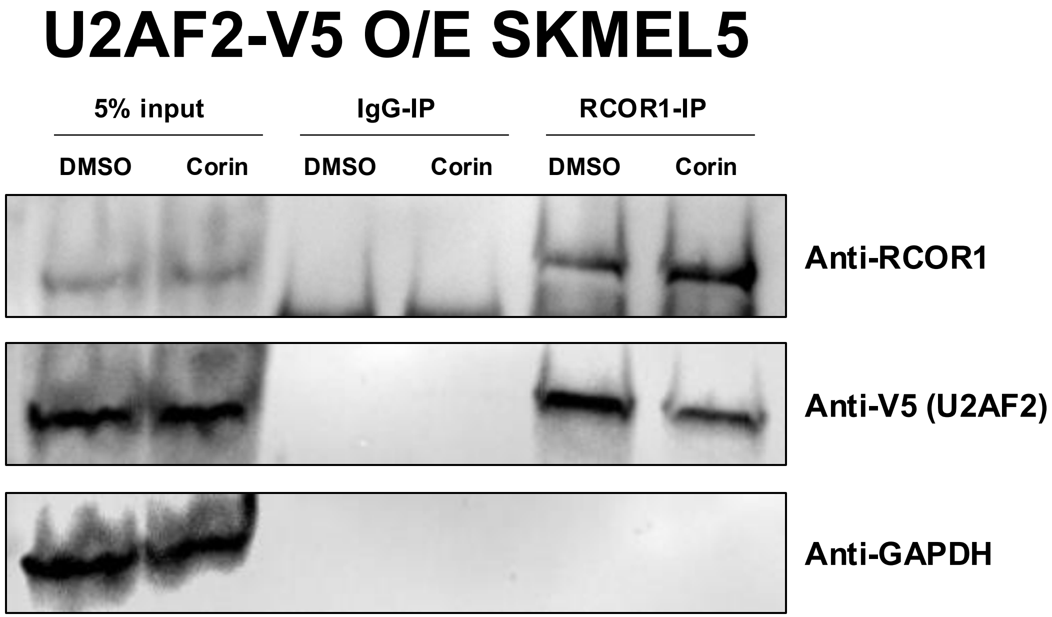

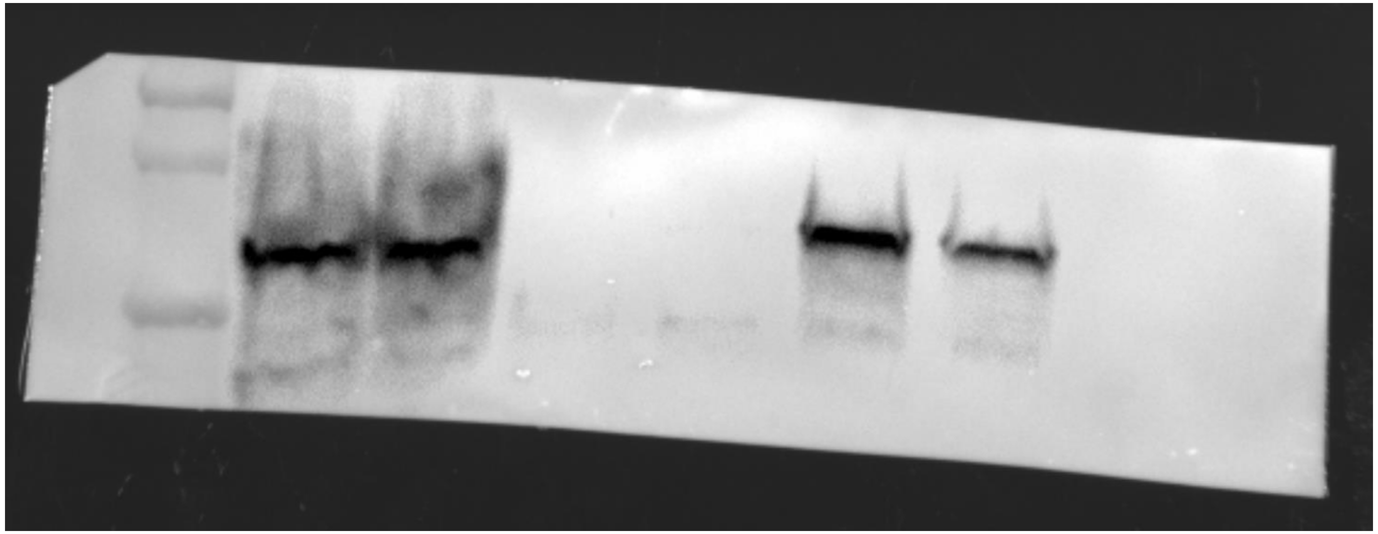

- In this experiment, only U2AF2-V5 O/E SKMEL5 cells are used, excluding WT SKMEL5.

Replicate 2: GAPDH

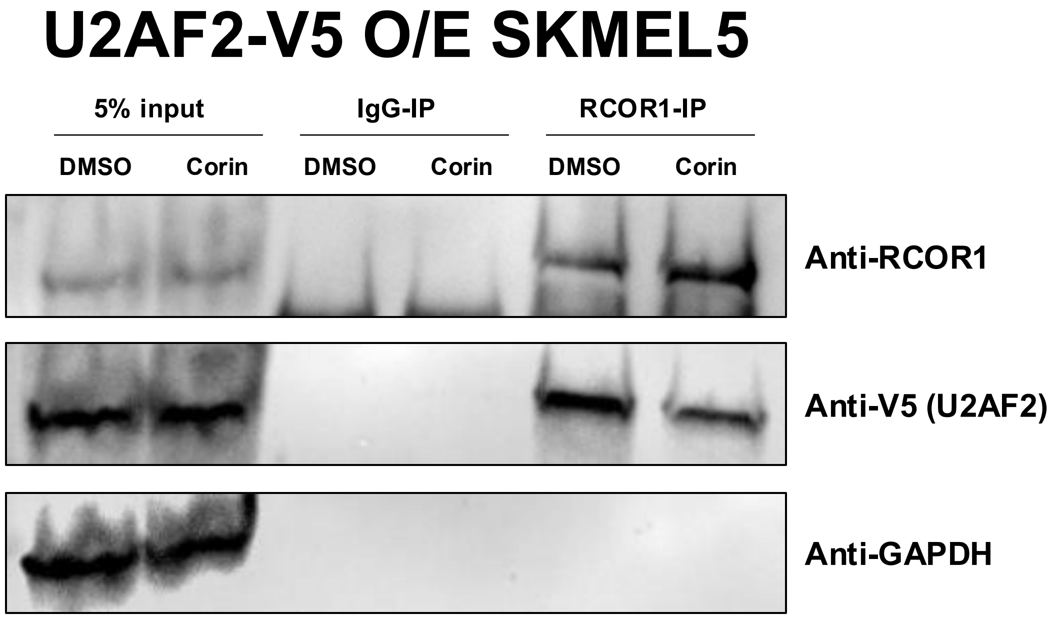

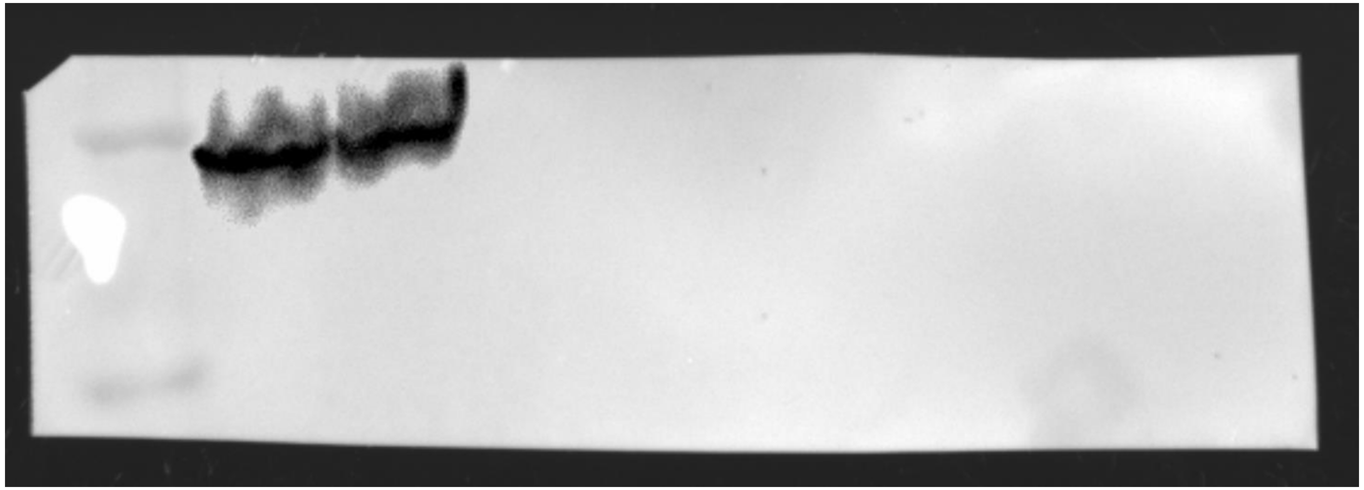

- In this experiment, only U2AF2-V5 O/E SKMEL5 cells are used, excluding WT SKMEL5.

Figure 3F, Extended Figure 5C

Gels – SKMEL5 cell lines treated with DMSO, GSK-LSD1, MS275, GSK-LSD1 + MS275, or corin (24h, 2.5uM). PCR products for TJP1 exon 20, MYO1B exon 23, FN1 exon 33

Replicate 1&2: FN1

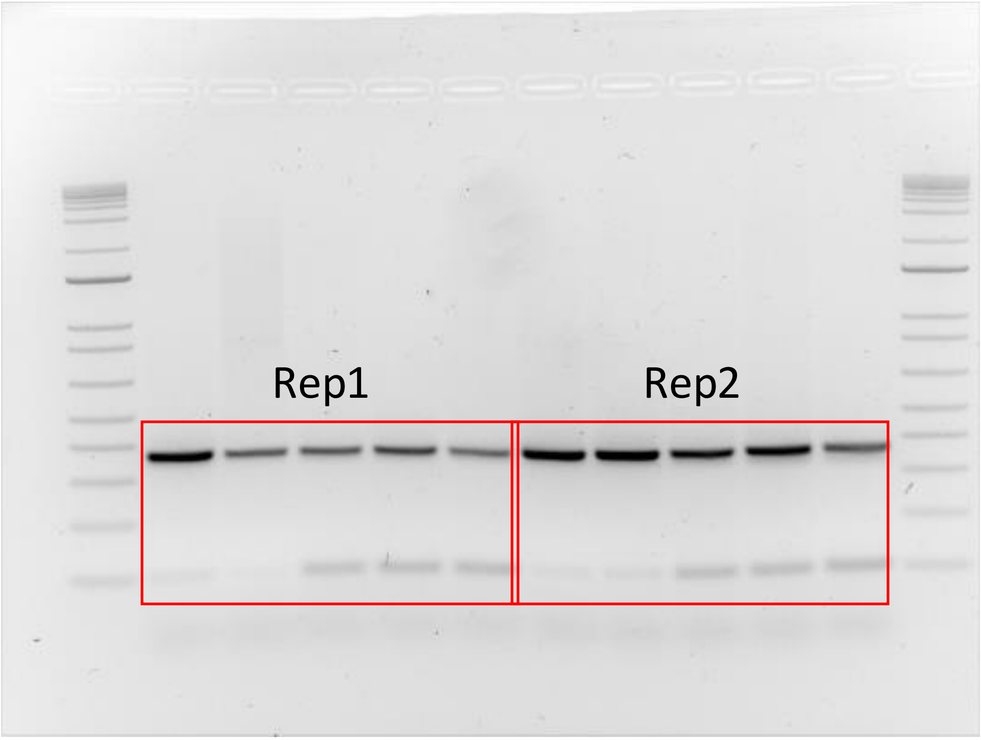

Replicate 3: FN1

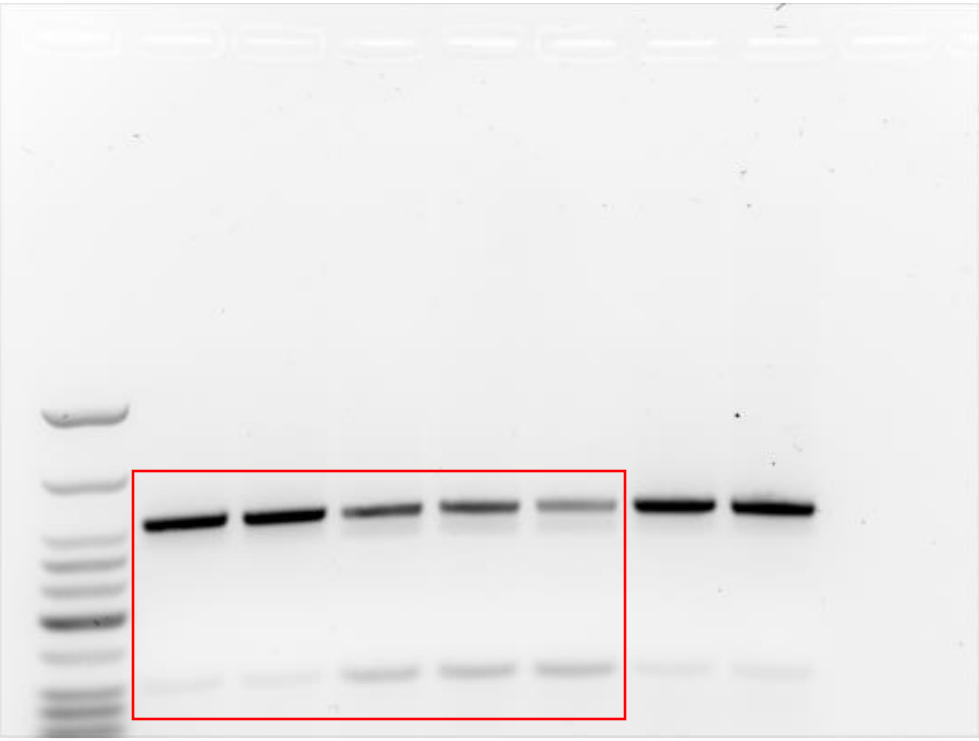

Replicate 1&2: TJP1

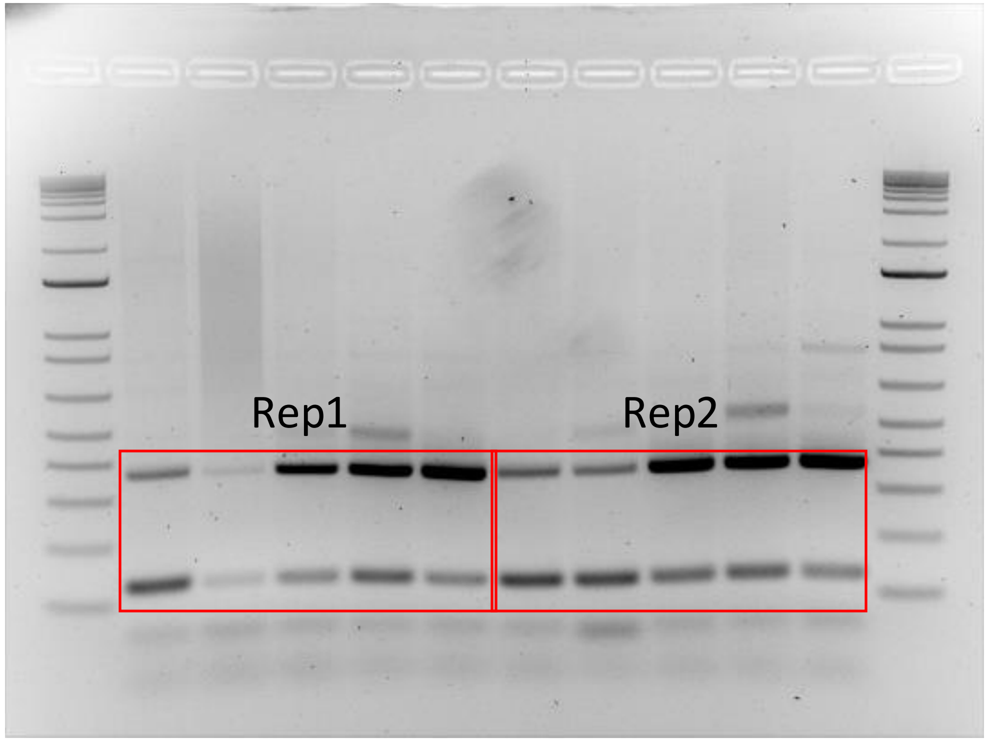

Replicate 3: TJP1

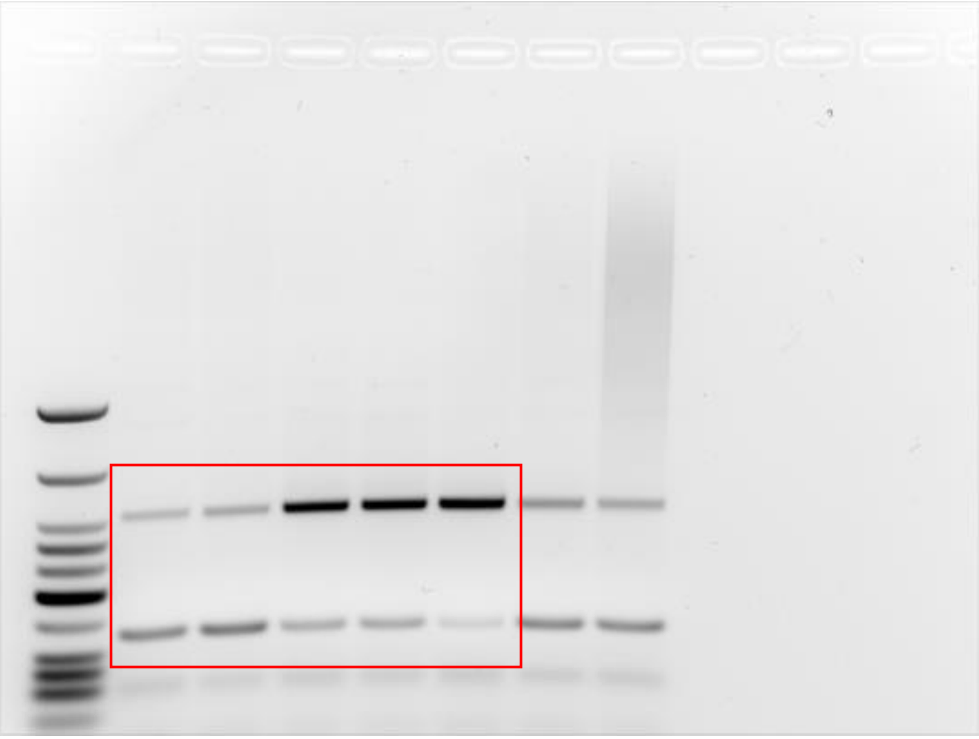

Replicate 1&2: MYO1B

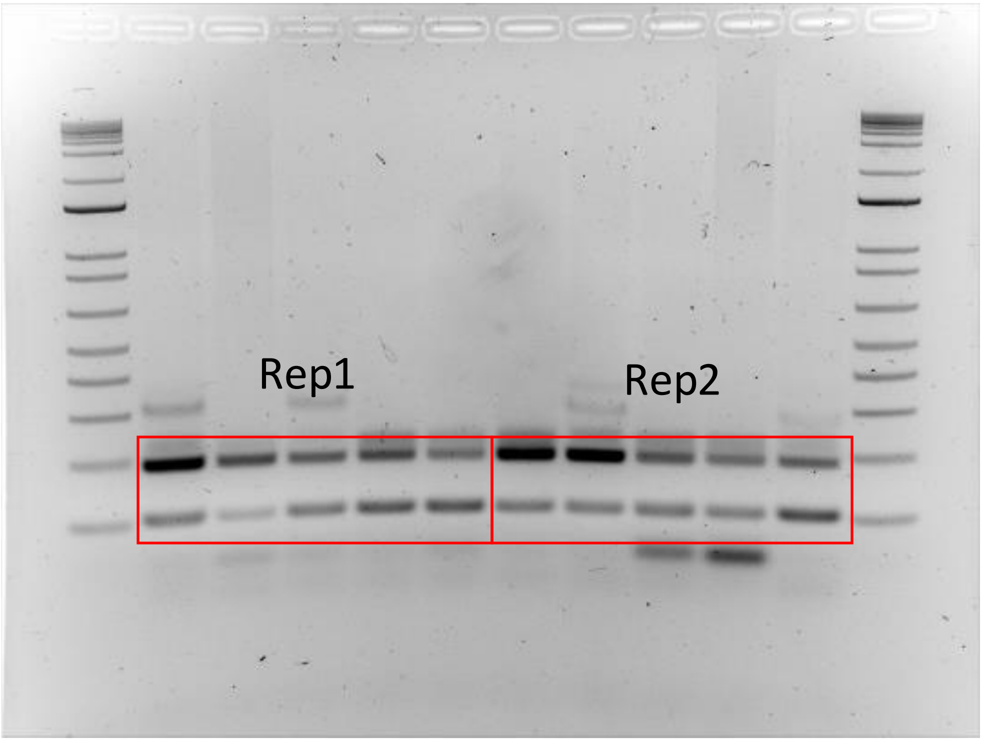

Replicate 3: MYO1B

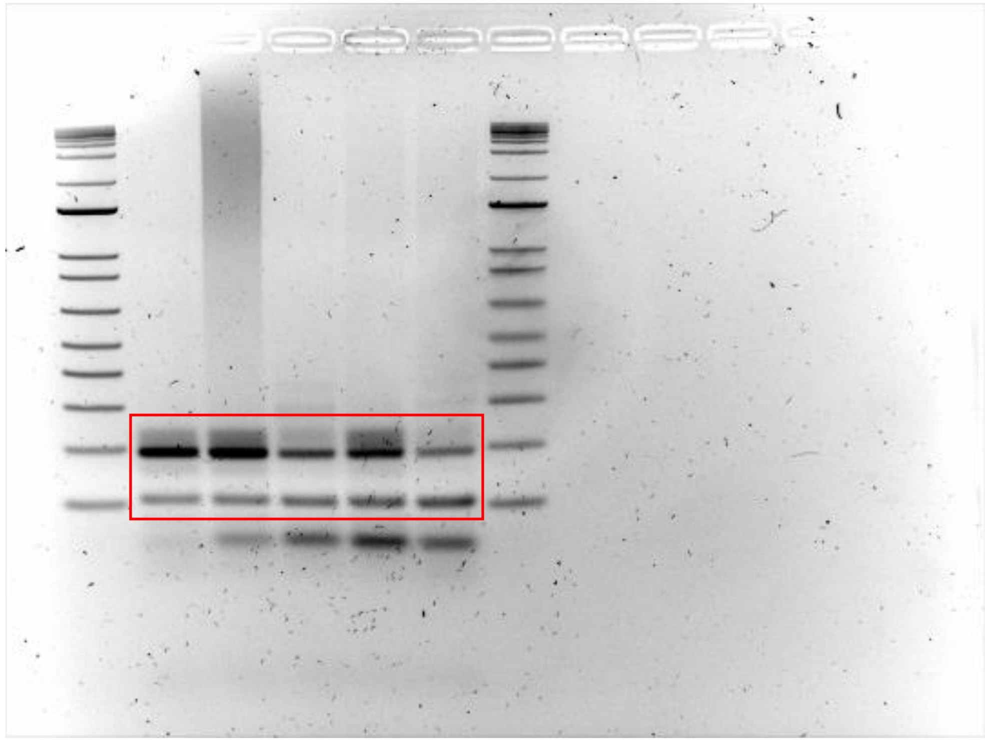

Supplement: Unedited blot and gel images [file jciinsight-11-190287-s074.pdf]
